# Detection Theory in Identification of RNA-DNA Sequence Differences Using RNA-Sequencing

**DOI:** 10.1371/journal.pone.0112040

**Published:** 2014-11-14

**Authors:** Jonathan M. Toung, Nicholas Lahens, John B. Hogenesch, Gregory Grant

**Affiliations:** 1 Genomics and Computational Biology Graduate Program, University of Pennsylvania School of Medicine, Philadelphia, PA, United States of America; 2 Institute for Biomedical Informatics, University of Pennsylvania School of Medicine, Philadelphia, PA, United States of America; 3 Institute for Translational Medicine and Therapeutics, University of Pennsylvania School of Medicine, Philadelphia, PA, United States of America; 4 Department of Genetics, University of Pennsylvania School of Medicine, Philadelphia, PA, United States of America; 5 Department of Pharmacology, University of Pennsylvania School of Medicine, Philadelphia, PA, United States of America; University of California, Los Angeles, United States of America

## Abstract

Advances in sequencing technology have allowed for detailed analyses of the transcriptome at single-nucleotide resolution, facilitating the study of RNA editing or sequence differences between RNA and DNA genome-wide. In humans, two types of post-transcriptional RNA editing processes are known to occur: A-to-I deamination by ADAR and C-to-U deamination by APOBEC1. In addition to these sequence differences, researchers have reported the existence of all 12 types of RNA-DNA sequence differences (RDDs); however, the validity of these claims is debated, as many studies claim that technical artifacts account for the majority of these non-canonical sequence differences. In this study, we used a detection theory approach to evaluate the performance of RNA-Sequencing (RNA-Seq) and associated aligners in accurately identifying RNA-DNA sequence differences. By generating simulated RNA-Seq datasets containing RDDs, we assessed the effect of alignment artifacts and sequencing error on the sensitivity and false discovery rate of RDD detection. Overall, we found that even in the presence of sequencing errors, false negative and false discovery rates of RDD detection can be contained below 10% with relatively lenient thresholds. We also assessed the ability of various filters to target false positive RDDs and found them to be effective in discriminating between true and false positives. Lastly, we used the optimal thresholds we identified from our simulated analyses to identify RDDs in a human lymphoblastoid cell line. We found approximately 6,000 RDDs, the majority of which are A-to-G edits and likely to be mediated by ADAR. Moreover, we found the majority of non A-to-G RDDs to be associated with poorer alignments and conclude from these results that the evidence for widespread non-canonical RDDs in humans is weak. Overall, we found RNA-Seq to be a powerful technique for surveying RDDs genome-wide when coupled with the appropriate thresholds and filters.

## Introduction

Next-generation sequencing technology provides comprehensive sequence information. The precision afforded by RNA-Seq is useful for studying various aspects of the transcriptome such as alternative splicing [1], [2], RNA editing [3], [4], and differential allelic expression [5]–[7]. RNA editing refers to co- or post-transcriptional modification of RNA, resulting in a transcript that is different from the underlying genomic template. In humans, two types of RNA editing processes are known to occur: adenosine deamination by ADAR results in A-to-G edits [8], [9] and cytidine deamination by APOBEC1 results in C-to-U changes [10], [11].

In recent years, many genome-wide surveys of RNA editing in humans have been performed using next-generation sequencing technology [3], [12]–[15]. In addition to the known A-to-G and C-to-U alterations introduced by RNA editing, researchers have reported the existence of RNA-DNA sequence differences (RDDs) that cannot be explained by known mechanisms [14], [16], [17]. However, the validity of these results is contested, as many reports cite experimental and technical artifacts as the main determinants of such systematic sequence differences between RNA and DNA [15], [18]–[21].

Current methods for the accurate identification of RDDs mainly involve ad hoc filters aimed at removing false positives [14], [15], [22]. In this study, we used a detection theory approach to evaluate the relative effect of misalignment and sequencing error on RDD analysis. In particular, we generated simulated RNA-Seq datasets containing simulated RDDs and assessed the performance of various RNA-Seq aligners in accurately identifying RDDs. We also analyzed filtering methods for their efficacy in achieving low false discovery rates of RDD detection and high sensitivity values. Lastly, after determining the optimal thresholds and parameters for sequence difference analysis, we searched for the presence of RDDs in an experimental human RNA-Seq dataset for which deep DNA and RNA sequence information is publicly available.

Overall, our report aims to explore the phenomenon of RDDs in humans as well as provide a framework for those interested in the study of RNA editing, RDDs, or differential allelic expression by elucidating the appropriate thresholds and parameters for accurate detection of allele-specific differences in RNA-Seq data. The simulated datasets generated in this study are publicly available for download (see Methods).

## Results

### Simulated RNA-Seq datasets

To evaluate the performance of various alignment algorithms and filtering methods in detecting RDDs, we generated simulated RNA-Seq datasets containing RDDs (see Methods). First, we created a “clean” dataset (dataset 1) with no sequencing errors or intronic reads in order to evaluate the degree of bias introduced by alignment error alone. Next, in order to capture the effect of sequencing error on RDD identification, we generated a more realistic RNA-Seq dataset containing substitutional sequencing errors, indel polymorphisms, intronic signal, and lower quality bases at the tail end of reads. We used a simplistic error model in which sequencing errors occur randomly and independently. We considered the effect of non-random sequencing errors and found their presence to be minimal in Illumina Hi-Seq datasets (see Methods). Both datasets contain 50 million pairs of non strand-specific reads of length 100 base pairs (bp) and were generated in triplicates to allow for assessment of variation in our various metrics.

Datasets were aligned using GSNAP, MapSplice, RUM, and Tophat2 (see Methods). For both dataset 1 and 2, GSNAP and MapSplice performed the best in terms of the number of reads mapped in total and uniquely (Table S1 in File S1), aligning approximately 99% of the 50 million read pairs. In contrast, RUM and Tophat2 aligned approximately 98% of the read pairs in dataset 1, but only roughly 95% in dataset 2, which contains sequencing errors. Overall, between 97 to 99% of the read pairs are aligned uniquely with GSNAP, MapSplice, and RUM, whereas only approximately 89% of the read pairs are aligned uniquely with Tophat2.

### Simulated RNA-DNA sequence differences

For each of the two datasets, we randomly introduced RDDs throughout the genome (see Methods). Namely, at positions containing RDDs, a percentage of the total reads at the site bear a randomly chosen non-reference allele representing the sequence difference. Furthermore, we define the percentage of reads containing the non-reference base to be the RDD level.

For each dataset, we generated approximately 600,000 total RDDs each in order to obtain reasonable sample sizes for making statistical inferences. Our motivation in choosing this number was not to simulate known frequencies or features of RNA editing events, but rather to accurately probe the ability of next-generation sequencing technology to detect hypothetical sequence differences in the human genome. Each RDD type is equally represented, with sequence differences that originate from cytosine and guanosine (C>A, C>G, C>T, G>A, G>C, G>T) slightly overrepresented than other types. This variation results from differing base compositions throughout the genome, with the effect more pronounced in dataset 2, which contains reads from intronic regions of the genome (Figure S1).

The coverage, or the total number of reads, at a given site is important in the analysis of RDDs as the presence and levels of RDDs at sites that are deeply sequenced are more likely to be robustly assessed (Figure S2). In order to assess the effect of sequencing depth on RDD detection, we stratified sites in the genome according to coverage and simulated equal numbers of RDDs in each group (see Methods).

For each RDD, we chose the level, or proportion of reads carrying the non-reference base, from a standard uniform random distribution excluding 0. However, because of the discrete nature of coverage, the distribution of RDD levels is not uniform at sites with low coverage; for sites with coverage greater than 100x, the distribution of levels is uniform across all levels with the exception of boundary values (Figure S3).

To understand the effect of hyperediting by ADAR and the observation that non-canonical RDDs often cluster [14], we modeled a subset of RDDs to occur in close proximity of one another (see Methods). Hyperediting refers to a type of editing by members of the ADAR family whereby approximately 50% of the adenosines on each strand of an RNA duplex is edited in a promiscuous fashion [23]. For each dataset, we generated approximately 2,000 clusters of length 100 bp within which approximately 50% of all positions with the same reference base bear the same type of RDD (see Methods).

Overall, in dataset 1, the average distance between neighboring RDDs is 10 bp (median 3 bp) for sites belonging to hyperedited clusters and 815 bp (median of 58 bp) for those that do not. For dataset 2, which contains intronic reads, the average distance between RDDs is also 10 bp (median 3 bp) for sites belonging to hyperedited clusters and 1565 bp (median 225 bp) for those that do not (Table S2 in File S1).

### Sensitivity of RNA-DNA sequence difference detection

We began our assessment of the performance of next-generation sequencing technology in identifying RDDs by analyzing the sensitivity or true positive rate of sequence difference identification. We will address the false negative and false positives rates of RDD detection in a subsequent section. We start by defining a simulated RDD as being properly identified by the aligner if at least one read bearing the non-reference base is observed. For dataset 1, we found that overall, GSNAP detected 96.32±6.19E-2% of the simulated RDDs, whereas MapSplice, RUM, and Tophat2 correctly identified 95.30±1.61E-1%, 95.36±1.63E-1%, and 95.04±1.38E-1%, respectively. For dataset 2, which contains sequencing errors and intronic reads, GSNAP identified 93.54±1.04E-1% of all simulated sites, whereas MapSplice, RUM, and Tophat2 found 92.34±1.16E-1%, 91.12±1.33E-1%, and 90.84±1.54E-1%, respectively.

**Figure 1:**
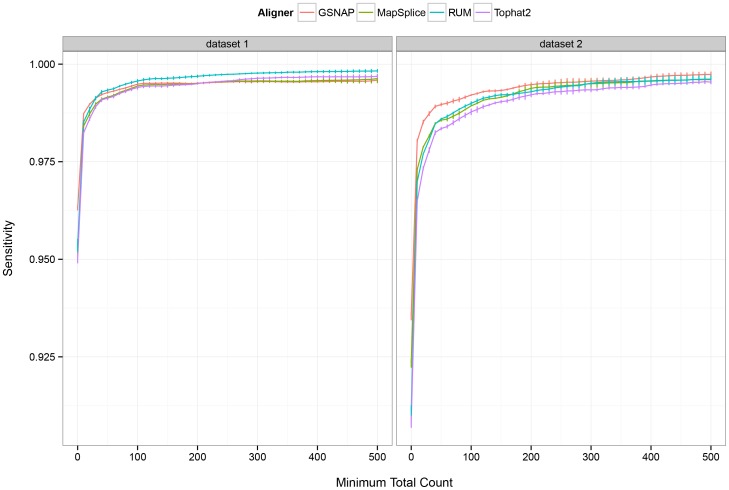
Sensitivity of RNA-DNA sequence difference detection versus coverage. The sensitivity or true positive rate of RNA-DNA sequence difference identification is shown versus various thresholds on the minimum depth of coverage required at the site of the simulated difference. For all four aligners, the true positive rate increases sharply upon raising the minimum depth of coverage required for detection from 0x to approximately 50x, after which it plateaus.

Next, we investigated the effect of sequencing depth or coverage on the detection of RDDs. We observed that for both datasets, the sensitivity of detection increases with higher thresholds on the minimum depth of coverage (Figure 1). For example, the sensitivity of sequence difference detection using GSNAP increases approximately 2 to 4% in datasets 1 and 2 when sites with coverage lower than 10x are removed from consideration. The sensitivity of RDD detection using MapSplice, RUM and Tophat2 increases in a similar fashion with higher coverage (Table S3 in File S1). Given the relatively low true positive rate or high false negative rate of RDD detection for locations with low coverage, we restrict subsequent analyses to sites with a minimum of 10 total reads in the simulated dataset and the corresponding aligned datasets per GSNAP, MapSplice, RUM, or Tophat2.

**Figure 2:**
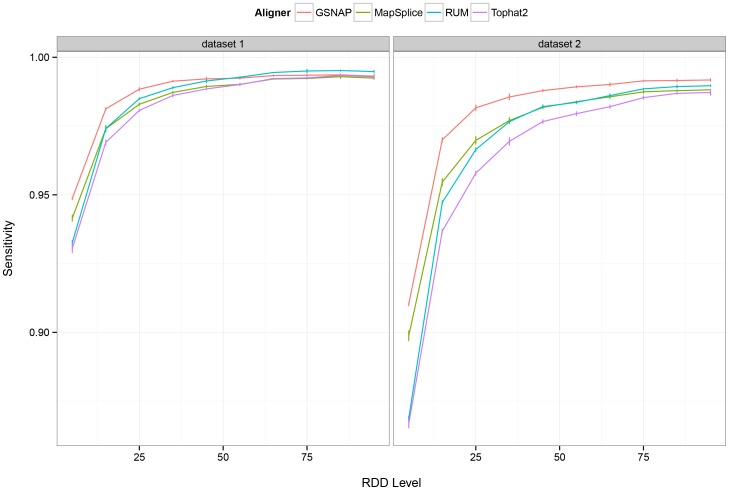
Sensitivity of RDD detection versus the simulated RDD level. Here we depict the true positive rate of RDD detection versus the simulated RDD level, or the percentage of reads at the site bearing the sequence difference allele. A minimum of 1 read bearing the RNA-DNA sequence difference is sufficient for a site to be deemed correctly identified. Sites with coverage less than 10x per the simulated RNA-Seq dataset are removed from consideration.

Next, we analyzed the effect of RDD level on the sensitivity of RDD detection. We binned the simulated sequence differences into 10 groups by RDD levels and evaluated the true positive rate for each group. For both datasets, we found the sensitivity of RDD detection to increase with higher RDD levels (Figure 2; Table S4 in File S1). Furthermore, GSNAP had the highest sensitivity values across all levels among the four aligners. Given the lower recall rates for sequence differences with low levels, we restrict our downstream analyses to sites with a minimum level of 10%.

Next, we analyzed whether the repetitive nature of the genomic sequence flanking the RDD site affects the detection of RDDs. We investigated this question by evaluating the sensitivity of detection in regions of the genome that are deemed non-unique by BLAT (see Methods). We observed that the true positive rate of RDD detection using GSNAP in non-unique regions according to BLAT is lower than that in unique regions by approximately 5% (Figure S4). For GSNAP, the average sensitivity in non-unique versus unique regions is 94.98±10.73E-2% versus 99.53±28.42E-4% for dataset 1 and 94.74±14.35E-2% versus 99.25±2.58E-2% for dataset 2. For GSNAP, MapSplice, and Tophat2, the difference in sensitivity of detection between RDDs within non-unique versus unique regions is roughly 4 to 5%, while for RUM, it is interestingly less than 1% (Table S5 in File S1). Upon further investigation, we attributed this difference in sensitivity patterns between the four aligners to different reporting procedures: for reads that align to multiple locations of the genome, GSNAP, MapSplice and Tophat2 will distinguish between primary and secondary alignments, whereas RUM does not. We also examined the sensitivity of RDD identification for sites lying within versus outside of RepeatMasker regions [24] and observed that for all four aligners, the sensitivity of detection is approximately 1 to 2% higher for sites lying outside of RepeatMasker regions (Table S6 in File S1).

Lastly, we analyzed the effect of proximity to neighboring RDDs on sensitivity of detection. Short-read aligners typically have a limit on the number of mismatches relative to the reference permitted in a reported alignment, and thus sites with many neighboring sequence differences may be harder to identify. We observed that the sensitivity of sequence difference detection for sites that are greater than 10 bp in distance away from a neighboring sequence difference is roughly 1 to 3% higher for dataset 1 and 3 to 6% higher for dataset 2 (Table S7 in File S1).

### Correlation between simulated versus observed levels of RNA-DNA sequence differences

**Figure 3:**
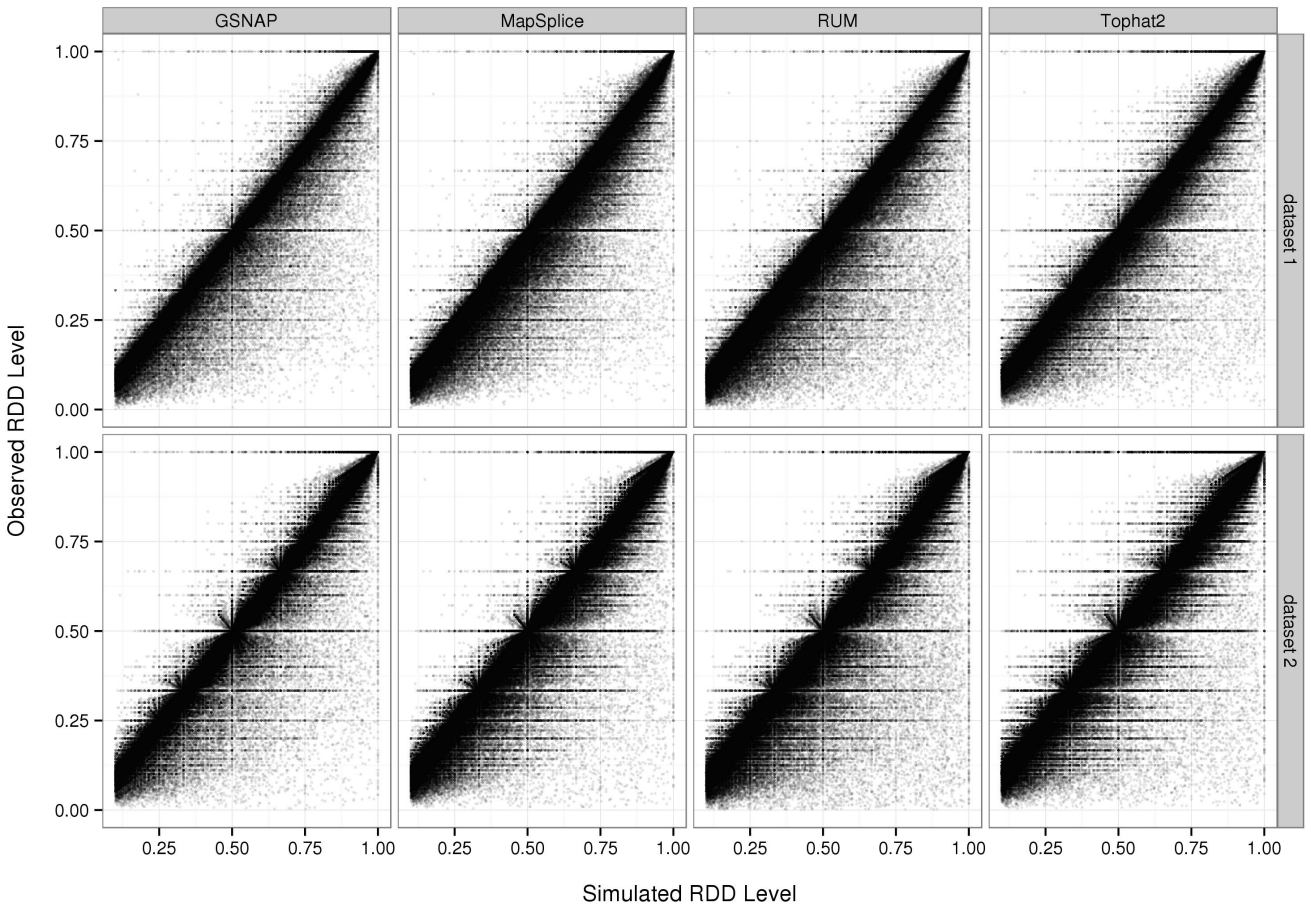
Simulated versus observed levels of RNA-DNA sequence differences. Here we plot the simulated RDD level versus the observed level as determined by GSNAP, MapSplice, RUM, or Tophat for replicate 1. Sites with coverage less than 10x or a RDD level less than 10% per the simulated dataset are removed from consideration. Overall, we observed the correlation between simulated and observed levels to be approximately 98% in both datasets and across the various aligners and replicates.

In many studies, the mere detection of RDDs is not sufficient. For example, in studies on RNA editing or differential allelic expression, information about the degree or level of difference is important. Here we analyzed the correlation between simulated and observed RDD levels. Based on our previous analyses, we restricted our study to sites with a minimum coverage of 10x, minimum level of 10%, and minimum of 1 read bearing the sequence difference base. Using this threshold, we calculated the correlation between observed and simulated RDD levels to be relatively high, at approximately 98 on average across all three replicates for all four aligners and both datasets (Figure 3; Table S8 in File S1). Although the simulated and observed levels correspond well, we found that roughly 20 to 40% of sites in each dataset for any aligner have observed levels that deviate from the simulated values by more than 5% (Figure S5). In particular, we found that in the majority (75 to 90%) of cases in which the observed and simulated levels deviate by at least 10%, the observed level underestimates the simulated level.

We hypothesized that one contributing factor to the discrepancy in RDD levels is the uniqueness or the ability of the region surrounding the site to be aligned accurately. Indeed, we found that approximately 22 to 34% of sites in which the simulated versus observed RDD levels differ by more than 30% are found in non-unique regions of the genome as determined by BLAT versus roughly 7 to 12% for those where the levels do not differ by 30% or more (Table S9 in File S1).

### Receiver operating characteristic and false positive analysis of RDD detection

Next, we analyzed the false positive and false discovery rates of RDD detection by evaluating the presence of differences at sites that were not simulated to represent RDDs. Using parameters we identified from our sensitivity analysis, we performed a receiver operating characteristic analysis on RDD detection genome-wide in each of the datasets (Table S10 in File S1). Overall, we observed that using a ‘minimum coverage of 10x, minimum level of 10%, and minimum of 1 read bearing the RDD base’ cutoff, the false positive rate of sequence difference detection is low, averaging 3.39E-2% and 6.47E-1% across the different aligners for datasets 1 and 2, respectively. However, these low false positive rates are not unexpected, as the vast majority of sites in the genome do not contain simulated RDDs.

**Figure 4:**
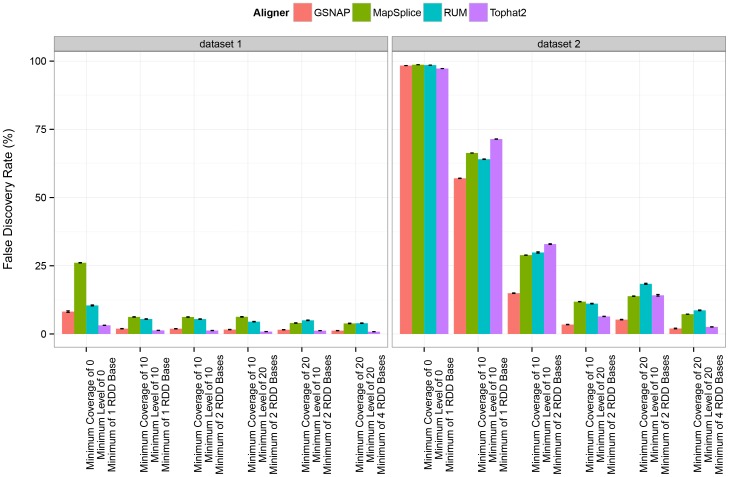
False discovery rate of RNA-DNA sequence difference detection. Here we depict the false discovery rate of RNA-DNA sequence difference detection under various thresholds on the coverage, level of sequence difference, and number of reads bearing the sequence difference base per the aligner. Calculations are averaged across the three replicates and error bars represent standard deviation values.

For a better understanding of how false positives affect the analysis of RDDs, we evaluated the false discovery rate (FDR), or the percentage of sites identified as having sequence differences that were not simulated to represent RDDs. For dataset 1, we found the FDR to range from 1.31±4.06E-2% in Tophat2 to 6.24±8.26E-2% in MapSplice when using a ‘minimum coverage of 10x, minimum level of 10%, and minimum of 1 one read bearing the RDD base’ threshold. These relatively low false discovery rates indicate that in the absence of sequencing error, misalignment issues do not contribute significantly to the incidence of false positives. With the introduction of sequencing error in dataset 2, we found that the false discovery rates are much higher, ranging from approximately 57% in GSNAP to 71% in Tophat2. These results are not surprising, as a threshold requiring only one read to bear the RDD base introduces false positives at sites with sequencing errors. With stricter thresholds on RDD detection, such as requiring a minimum coverage of 20x, a minimum RDD level of 20%, and a minimum of 4 RDD bases observed, we found that the false discovery rate decreases dramatically (Figure 4).

### Evaluation of filters in reducing false positives

Many previous studies on RNA editing and RDDs attempt to remove false positive sites using various filters [3], [14], [15], [17], [22]. We investigated the effectiveness of some of these measures in eliminating false positives.

The first filter we analyzed requires RDDs to be identified concordantly by other aligners in order to be considered valid. We hypothesize that this condition will minimize the contribution of aligner-specific biases to the problem of false positive RDDs. For each aligner, we calculated the number of true and false positives remaining after RDDs that were not found by other aligners were removed (Table S11 in File S1). Using a ‘minimum coverage of 20x, minimum level of 20%, and a minimum of 4 reads bearing the sequence difference’ to identify RDDs, we observed the false discovery rates of RDD detection decreased with increasing numbers of other aligners required to concordantly identify the RDD (Figures S6-S9). In both datasets, requiring concordance among GSNAP, MapSplice, and RUM removed approximately 30% to 50% of false positives in GSNAP to 60 to 90% in MapSplice and RUM, whereas approximately only 2 to 12% of true positives were removed (Table S11 in File S1). We observed that while requiring concordance with Tophat2 led to the largest reductions in the number of false positives, it also led to large (over 50%) decreases in the number of true positives; this is expected as we previously observed Tophat2 to identify the fewest number of RDDs among the four aligners (Table S10 in File S1).

The second filter we analyzed involves using BLAT to determine whether the sequence surrounding the RDD site can be aligned to other homologous regions of the genome (see Methods). Using a ‘minimum coverage of 20x, minimum level of 20%, and minimum of 4 reads bearing the sequence difference’ to identify RDDs, we observed that the BLAT method removes approximately 14% and 28% of false positives found by GSNAP in datasets 1 and 2 respectively, but only filters out roughly 1 to 5% of true positives in either datasets (Table S12 in File S1). As expected, we observed that the performance of the BLAT filter varies depending on the repetitive nature of the underlying flanking sequence. For example, within RepeatMasker regions, approximately 22% of false positives and 19% of true positives within the dataset 1 for GSNAP are filtered out, whereas outside of RepeatMasker regions, roughly 14% of false positives are removed compared to less than 1% of true positives (Table S12 in File S1). Interestingly, the difference between the percentage of false versus true positives removed by the BLAT method is largest for RUM, followed by MapSplice, GSNAP, and Tophat2 (Figure S13). Overall, we found that the BLAT filtering approach decreased the FDR of RDD detection for GSNAP by approximately 13% in dataset 1 and 24% in dataset 2 (Figure S14; Table S13 in File S1).

Pseudogenes are non-functioning homologs of genes that are either not expressed or unable to be translated into protein product, and their high sequence similarity to functioning genes can result in false positive sequence difference calls. We observed that the removal of all sequence differences lying within pseudogenes as annotated by Gencode version 13 [25] decreases the FDR of RDD detection using GSNAP by approximately 45 to 50% in both datasets (Table S14 in File S1).

Misalignments near exon-exon junctions can commonly lead to the identification of false positive sequence differences. We evaluated the effect of such incorrectly spliced alignments on sequence difference detection and found that roughly 2% of the false positives identified by GSNAP in dataset 1 and 5% of those found in dataset 2 are in intronic sequences within 6 bp of exon-exon junctions. Removal of all sites in introns within 6 bp of splice junctions leads to a roughly 2 to 4% decrease in the false discovery rate for GSNAP. RUM and Tophat2 are more robust to misalignments near splice junctions, as less than 1 to 2% of false positives detected by either aligner are in introns near exon-exon junctions (Table S15 in File S1), whereas 40 to 50% of false positives identified by MapSplice are found near junctions.

Finally, we analyzed the effect of implementing the various bioinformatics filters in concert on the false positive rates. In analyzing the RDDs obtained using the ‘minimum coverage of 20x, minimum level of 20%, and minimum of 4 reads containing the sequence difference allele’, we observed that requiring concordance with at least one other aligner, the BLAT filtering method, removal of differences in pseudogenes, and elimination of intronic sites within 6 bp of exon junctions in combination removed roughly 50 to 90% of false positives depending on the aligner versus roughly 3 to 11% of true positives (Table S16 in File S1). Across the different aligners, these various filters led to a decrease of approximately 50 to 90% in the FDR of RDD detection (Table S17 in File S1), whereas sensitivity changed by approximately 2 to 11%.

### Evaluation of RNA-DNA sequence differences in human lymphoblastoid cell line

Lastly, to evaluate the performance of our pipeline on a real experimental dataset, we analyzed the human lymphoblastoid cell line GM12878, for which deep DNA and RNA sequence is readily available [26]. We used the parameters and thresholds as determined from our previous simulated data analyses to identify RDDs. In particular, we aligned two replicates of RNA-Seq data, each containing approximately 120 million 76 bp paired-end reads, using GSNAP (Table S18 in File S1) and identified RDDs using a ‘minimum coverage of 20x, minimum level of 20%, and minimum of 4 reads containing the sequence difference base’ threshold. Sequence differences found in dbSNP137 [27] were removed from consideration. Furthermore, to minimize the detection of sequence differences resulting from sequencing error, we focused our analysis on those differences that are observed in both replicates (Table S19 in File S1).

**Figure 5:**
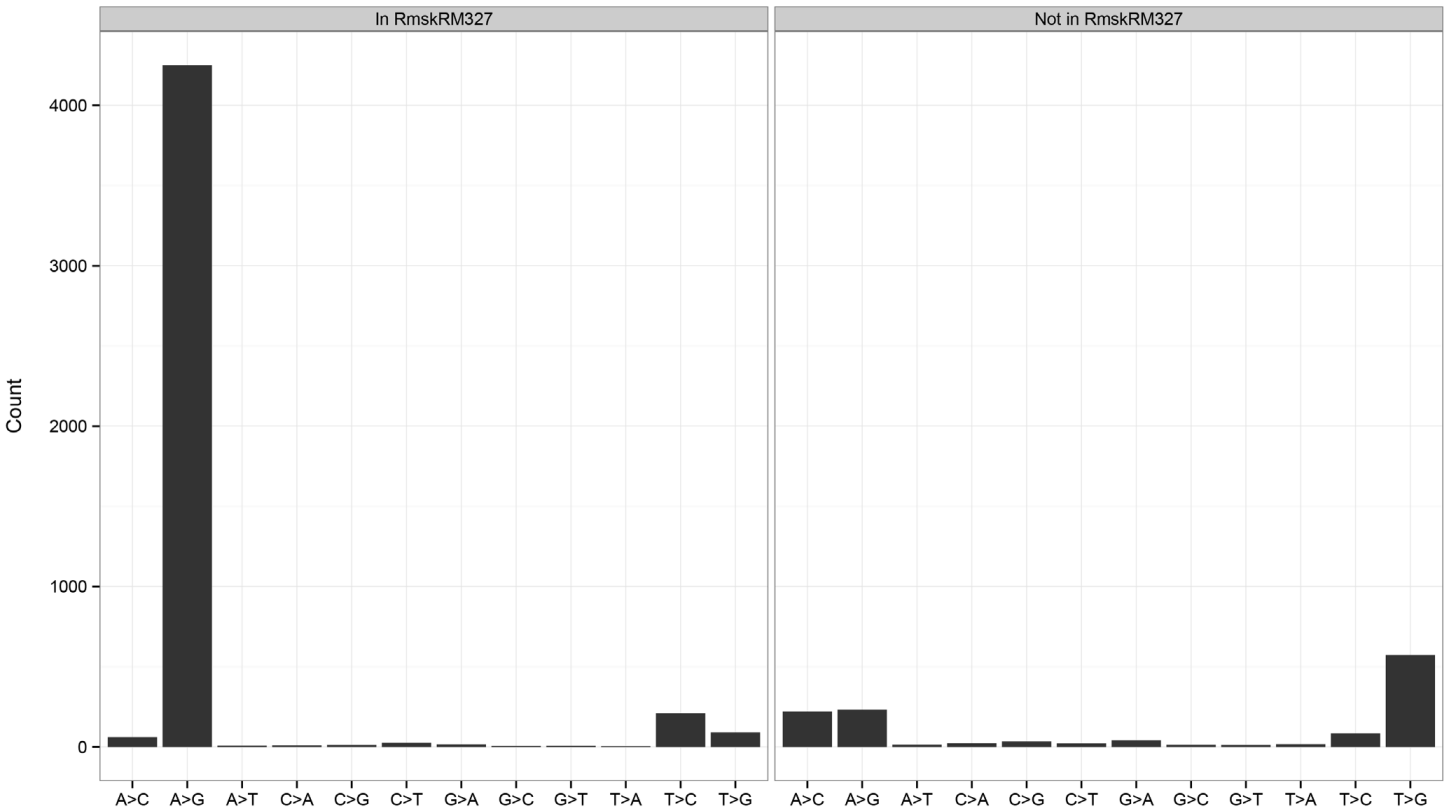
Distribution of RNA-DNA sequence differences in GM12878. Here we depict the distribution of RNA-DNA sequence differences in GM12878 after removing sites using various filters.

Next, we investigated the percentage of observed RDDs that are removed by filters we previously identified as effective in reducing the amount of false positive RDDs. Other researchers have used these filters in their pipelines to accurately identify RDDs [3], [15], [22]. The filters we implemented include requiring concordance with at least one other aligner, searching with BLAT for regions homologous to the sequence flanking the sequence difference (see Methods), removing intronic sites near exon-exon junctions, and eliminating differences in annotated pseudogenes or adjacent to homopolymer sequences. We applied the BLAT filter to sequence differences found outside of RepeatMasker regions, as we previously showed that this filter is not as effective in discriminating between true and false positives within repetitive sequences. We separated the differences by type into two groups: A-to-G sequence differences and non-canonical sequence differences, or changes that cannot be explained by known mechanisms. We note that although C-to-T differences can be mediated by APOBEC, APOBEC1 is not expressed in this B-cell cell line, with an FPKM value [28] of 0 in both replicates; thus we classify C-to-T changes as non-canonical. We observed that approximately 72% of non-canonical differences are removed by one or more of these filters, whereas only roughly 36% of A-to-G sites are eliminated (Table 1). The filtering steps that filtered out the greatest percentage of sites are the requirement of concordance with at least one other aligner and the pseudogene and BLAT filters, as nearly 30% to 45% of non-canonical sites are removed by each filter independently. After taking into consideration all of the filters we used, a total of 5,997 sequence differences remained (Figure 5), 75% of which are A-to-G edits and are likely to be mediated by RNA editing via ADAR. Of these 5,997 differences, the majority (78%) are located within RepeatMasker regions. Within RepeatMasker regions, 90% of the differences are A-to-G, as is expected due to the phenomenon of editing in human *Alu* elements [29]. In contrast, the majority (82%) of sites outside of RepeatMasker are noncanonical differences. The distribution of sequence differences we observed are highly concordant with other studies (Table S20 in File S1), and the most common noncanonical RDD types we observed were A-to-C (23%) and its complement T-to-G (54%), as previously seen by others [15], [17], [22].

**Table 1:** Number of RNA-DNA sequence differences removed by various bioinformatics filters.

	A>G	Non-canonical	Total
**Total before filters**	**7,036**	**5,444**	**12,480**
Concordance with at least one other aligner filter (removed)	1,937 (27.53%)	2,399 (44.07%)	4,336 (34.74%)
Pseudogene filter (removed)	847 (12.04%)	1,959 (35.98%)	2,806
BLAT filter (removed)	545 (7.75%)	1,722 (31.63%)	2,267
Homopolymer filter (removed)	32 (0.45%)	205 (3.77%)	237
Exon junction filter (removed)	30 (0.43%)	88 (1.62%)	118
**Total after filters (remaining)**	**4,484 (63.73%)**	**1,513 (27.79%)**	**5,997 (48.05%)**
Total after filters - in RmskRM327	4,251	452	4,703
Total after filters - not in RmskRM327	233	1,061	1,294

For the remaining RDDs that are not removed by our filtering methods, we asked whether features indicative of sequencing error or low-quality mapping are more common in non-canonical versus A-to-G sequence differences. Specifically, we noticed that many non-canonical sequence differences occur within regions where many of the reads overlapping the sequence difference site are either partially mapped via a local alignment with clipped bases or mapped with many mismatches (Figure S9). To investigate the mapping quality near sites of RDDs globally, we calculated for each read that overlaps an RDD the number of bases (out of the total 76 bp sequence) that are neither clipped nor aligned with a mismatch or indel; we refer to this figure as the number of bases aligned properly. We observed that in both replicates, for sequence differences in RepeatMasker, the overall number of bases that are aligned properly is higher for A-to-G changes than for the most of the non-canonical types, particularly the C-to-G type; for sites lying outside of RepeatMasker regions, the number of bases aligned properly for C-to-G and G-to-C are generally much lower than that for other types (Figure S10).

## Discussion

RNA-Sequencing is a powerful technology for genome-wide analyses of transcriptome information at the single-nucleotide level. The resolution afforded by next-generation sequencing technology has allowed for genome-wide studies on RNA editing in humans [3], [13] and led to the identification of all 12 types of sequence differences [16]. There are, however, limitations to high-throughput sequencing, as difficulties lie in the alignment of short sequencing reads and errors introduced by sequencing and library preparation among other challenges. The relative effect of these various misalignment and sequencing errors on the identification of RDDs is debated, although many reports assert that the majority of the non-canonical sequence differences observed result from technical artifacts [18]–[21]. In this study, we dissect the various sources of error leading to false positive RDDs and evaluate their relative contribution. Using a detection theory approach, we generated simulated RNA-Seq datasets containing known RDDs to evaluate the effect of alignment and sequencing error on RDD analysis. In the absence of sequencing error, we found that minimal thresholds are sufficient for sensitivity values above 95% and false discovery rates below 5%. Moreover, we found that the RDD levels reported by the various aligners correlation well (*R*∼98%) with the true levels per our simulation. Upon introduction of sequencing errors following a random and independent distribution, we found that a threshold requiring a ‘minimum coverage of 20x, minimum level of 20%, and minimum of 4 reads bearing the RDD base’ is necessary for false discovery rates below 10% across the various aligners.

Currently, most pipelines use ad hoc filtering methods to minimize the presence of false positives in sequence difference studies without a full understanding of the efficacy of these methods or the trade-off between sensitivity and false discovery rates. We found that overall while the various filters used in the literature for removal of false positive RDDs are effective in discriminating between true and false positives, a sizeable percentage (roughly 10 to 50% depending on the aligner) of false positives remain even after all filtering methods are implemented.

Lastly, we used our pipeline for identification of RDDs to evaluate the presence of sequence differences in humans. Using parameters and thresholds we deemed as optimal, we identified approximately 6,000 RDDs, the majority (75%) of which are A-to-G changes and likely to be mediated by ADAR. Of the non-canonical RNA-DNA sequence differences that remained after our filtering processes, we found A-to-C and its complement T-to-G to be most common. Notably, A-to-C changes have been found by others to be the most common sequencing error [30], [31]. Furthermore, we found that the alignments of reads overlapping non-canonical RNA-DNA sequence differences, with the exception of A-to-C and T-to-G types, contain many more mismatches or clipped bases than those of A-to-G differences. The distribution of sequence differences we observed is highly concordant with previous studies, and like others [15], [32], we conclude that there is little evidence for widespread non-canonical editing.

Overall, we observed that next-generation sequencing technology and current bioinformatics tools are a reliable and powerful technique for studying RDDs genome-wide. Furthermore, we found that computational biology methods are an effective means for evaluating the various thresholds and filtering techniques used to accurately identify sequence differences. Our results demonstrate that while RNA-Sequencing allows for precise detection and measurement of RDDs, current bioinformatics filters do not completely remove false positive calls. We aim for this study to provide a general framework for those interested in site-specific allelic differences in humans using RNA-Sequencing, and hope in particular that our work may shed light on the appropriate thresholds and necessary caution to employ for RDD analyses.

## Methods

### Simulation of RNA-Seq datasets

Simulated datasets were generated using the BEERS simulator [33]. Data are based on human build hg19 and RefSeq transcript models [34], as aligned to the genome by UCSC [35] using BLAT [36]. The expression intensities are Poisson distributed with probabilities estimated from roughly 300 million reads of human retina RNA-Seq data, as described previously [33]. Default settings result in 36,467 transcripts, of which approximately 70% are expressed. We simulated two types of RNA-Seq datasets. Dataset 1 was “clean” and designed to contain no intron signal or sequencing error. Dataset 2 was “realistic” and constructed with intron retention and sequencing error. We used a substitutional error rate of 1 in 200 (0.5%), a value comparable to sequencing error rates observed in Illumina Genome Analyzer IIx and HiSeq machines [37]. Furthermore, we simulated poorer quality bases at the 3′ ends of reads by increasing the substitutional error rate to 20% in the last 10 bases for 25% of the reads. Approximately 30% of the signal in the dataset originates from introns. These parameters are consistent with real data observations. Lastly, we also included indel polymorphisms at a rate of 1 in 1000 (0.1%). Both datasets 1 and 2 were generated in triplicate, with each replicate containing 50 million pairs of reads of length 100 base pairs (bp). The mean fragment length of each read pair is 330 bp. The simulated datasets are available at http://itmat-public.s3.amazonaws.com/toung_rdd-study_simulated.dataset1.tar and http://itmat-public.s3.amazonaws.com/toung_rdd-study_simulated.dataset2.tar for download.

### Analysis of non-random sequencing errors in Illumina HiSeq RNA-Seq datasets

To evaluate the presence of non-random sequencing errors in Illumina HiSeq datasets, we analyzed two replicates of a dataset (denoted IVT-Seq dataset replicates 1 and 2) comprising 1,062 cDNAs from the Mammalian Genome Collection (MGC) that were expressed *in vitro* and sequenced using Illumina HiSeq 2000 technology [38] to obtain approximately 41 million and 32 million 100-bp paired-end reads. In addition, we also sequenced the same 1,062 cDNA plasmids that served as the template for the IVT-Seq datasets using Illumina HiSeq 2000 technology (denoted plasmid dataset). We aligned all three datasets using GSNAP (see ‘Alignment of RNA-Seq datasets’ in Materials and Methods) to an index containing the non-spliced reference sequence of the 1,062 cDNAs. For the IVT-Seq datasets, approximately 82% of the total reads were aligned in the correct orientation and with the expected inner distance between read pairs. For the plasmid dataset, approximately 20% of the total reads align properly; this relatively low percentage is expected given the presence of plasmid backbone in the dataset. The coverage distribution in the three datasets is fairly uniform, with an average of approximately 2,600x and 3,000x in the IVT-Seq replicates 1 and 2, respectively (median of 2,300x in replicate 1 and 1,800x in replicate 2) and a mean of roughly 770x in the plasmid dataset (median of 700x). To be confident of the sequencing and alignment results, we restricted our analyses to sites with a minimum coverage of 1,000x in the IVT-Seq datasets and 250x in the plasmid dataset. In total, we obtained 4,923,509,994 and 4,195,153,516 bases of sequence at 1,209,658 and 1,111,552 sites and observed a total of 1,877,330 and 4,902,349 sequencing errors, giving an overall error rate of approximately 3.8×10^−4^ and 1.2×10^−3^ in IVT-Seq replicates 1 and 2, respectively. For the plasmid dataset, we obtained 1,340,601,515 bases of sequence at 1,377,516 sites and found 999,763 sequencing errors, giving an overall error rate of roughly 7.5×10^−4^. These sequencing error rates are all approximately 4x to 13x smaller than the rate of 0.5% we used in our simulated datasets.

For each site in a particular dataset, we calculated the sequencing error level to be the percentage of total reads at the site bearing an error. To test whether the observed sequencing errors occur randomly, we performed a Kolmogorov-Smirnov test, comparing the observed distribution of sequencing error levels to a null distribution derived from the overall error rate calculated previously. We found that for both of the IVT-Seq replicates as well as the plasmid dataset, the distribution of sequencing error levels deviates from that expected under the null distribution (*P*<0.001 for all three datasets). These results indicate that errors are not introduced randomly but occur at error levels that are higher than expected for particular sites. These observations are concordant with previous studies that demonstrate that sequencing errors introduced by Illumina next-generation sequencing platforms may occur in a sequence-specific or non-independent manner [37], [39]. These non-random sequencing errors are indistinguishable from RDD events if they occur at high error levels. We found the frequency of errors that (1) occur at levels of 20% or higher and (2) are reproducible across the three datasets to be 4.38×10^−5^ and 4.77×10^−5^ in the IVT-Seq replicates 1 and 2 respectively and 3.85×10^−5^ in the plasmid dataset. However, upon further Sanger sequencing validation, we found that the majority (71%) of these nonrandom sequencing errors were errors in the sequence of the clones and not errors introduced by Illumina sequencing. Correcting for the presence of such errors in the sequence, we estimate that the true frequency of nonrandom errors occurring at levels of 20% or greater to be approximately 4.77×10^−6^, which is one order of magnitude less than that of RDDs observed in our experimental datasets.

### Alignment of RNA-Seq datasets

RNA-Seq datasets were aligned using GSNAP version 2012-07-20 [40], MapSplice version 2.1.5 [41], RUM version 2.0.3-02 [33], or Tophat2 version 2.0.6 [42] to the human genome (build hg19). GSNAP was run with default options. A maximum number of 10 alignments were permitted for each read. Alignments to novel exon-exon junctions (per GSNAP option -N 1) and known junctions as defined by RefSeq (downloaded November 2, 2012) and Gencode version 13 [25] were accepted. Alignments with no more than the default maximum of ‘(read length +2)/12–2′ mismatches were retained. MapSplice and RUM were run with the default command line options. Tophat2 was run with the default options. A maximum edit distance and mismatch count of 6 was allowed for each read. Secondary alignments up to the default maximum of 20 were permitted. After alignment with GSNAP, MapSplice, RUM, or Tophat2, non-primary alignments and alignments placing read pairs in the incorrect orientation were removed.

### Simulation of RNA-DNA sequence differences

For each dataset, sites in the genome are first stratified by coverage to ensure the placement of RDDs at locations with varying depths of coverage. The distribution of coverage for dataset 1, which does not contain reads originating from intronic regions of the genome, is fairly uniform, while for dataset 2, the distribution is skewed right (Figure S2); approximately 82% of sites in dataset 2 have coverage of 10x or less compared to approximately 20% in dataset 1. For dataset 1, we grouped sites into quartiles, corresponding to coverage values of approximately 0x to 14x for quartile 1, 15x to 49x for quartile 2, 50x to 133x for quartile 3, and 134x and above for quartile 4. For dataset 2, the presence of introns results in a highly skewed right distribution for coverage. As such, we divided dataset 2 into one group containing sites with coverage below 10x and split the remaining sites into tertiles, corresponding to coverage values of approximately 11x to 19x for tertile 1, 20x to 48x for tertile 2, and 49x and above for tertile 3. After we grouped sites by coverage, we randomly inserted RDDs at different sites such that each coverage group contained approximately the same number of sequence difference sites.

The type of RDD difference (e.g. A-to-C, A-to-G, A-to-T, etc.) was determined randomly and independently for each site. The RDD level, or the proportion of reads containing the sequence difference, was chosen randomly from a random uniform distribution from 0 to 1, excluding 0.

A small subset (5%) of the simulated RDDs was randomly chosen to model hyperediting, or the clustering of many sequence differences in a small window. In particular, we designated all of the sites that are within 100 bp of the chosen site to have a 50% chance of having the same RDD type provided that the coverage belongs to the same coverage group as the initial site.

### Repetitive regions of the genome as defined by BLAT

As one measure of the repetitive nature of a region surrounding a sequence difference site, we used BLAT [36] to search for homologous sequences in the genome. In particular, we extracted flanking sequences of length 51 bp, 101 bp, and 151 bp around a given site and queried for alignments in the genome with BLAT (v.35x1). The settings —stepSize = 5 and —repMatch = 2253 were used to increase sensitivity. A maximum of (read length +2)/12 – 2 mismatches per alignment, the same amount permitted by GSNAP, was tolerated. Sites for which more than one alignment is found for one of the three flanking sequences are deemed “non-unique by BLAT”.

### Filtering of RNA-DNA sequence differences using BLAT

To ensure that an RDD identified by the various aligners cannot be explained by homologous sequences in the genome, sequences of length 25, 50, and 75 bp upstream and downstream of each sequence difference site were aligned to the genome using BLAT (v. 35x1). The settings —stepSize = 5 and —repMatch = 2253 were used to increase sensitivity. A maximum of (read length +2)/12 – 2 mismatches per alignment, the same amount allowed by GSNAP, was tolerated. An RDD was filtered out if any of the flanking sequences aligned to a region other than the RDD site and if that alignment explained the sequence difference.

### Analysis of BWA performance on RDD detection

We evaluated the performance of the Burrows-Wheeler Aligner (BWA) on RDD detection. We conducted these analyses separately from the other three aligners we used as BWA is not a RNA-Seq aligner capable of mapping across intron-size gaps. In particular, synthetic RNA-Seq dataset 1, which does not contain reads covering intronic sequences, was aligned using BWA version 0.7.9a-r786 [43] to a transcriptome index comprising exonic sequences as defined by a non-redundant union of several annotation efforts as published at the UCSC Genome Browser (RefSeq, UCSC Known, Vega, AceView, ENSEMBL). BWA was run with default options.

We defined a simulated RDD as being properly identified by BWA if at least one read bearing the non-reference base is observed. We found that the sensitivity of RDD detection by BWA is 92.96±1.94E-1% compared to approximately 95 to 96% for GSNAP, MapSplice, RUM and Tophat2. Sensitivity of detection increased by 4% when sites with coverage lower than 10x are removed from consideration. Restricting analysis to sites with a minimum coverage of 10x, minimum level of 10%, and minimum of 1 read bearing the sequence difference base, we found the correlation between observed and simulated RDD levels to be 97±1.57E-2%.

Next, we analyzed the false positive and false discovery rates of RDD detection by identifying sites that were not simulated to contain RDDs. Using a ‘minimum coverage of 10x, minimum level of 10%, and minimum of 1 read bearing the RDD base’ cutoff, we found the false positive rate and false discovery rate of RDD detection to be 1.54E-2±2.49E-4% and 1.81±4.22E-2%, respectively. Overall, our analyses on dataset 1 which comprises exonic regions of the genome showed that BWA provides comparable results to those reported by the other aligners we analyzed, namely GSNAP, MapSplice, RUM, and Tophat2.

## Supporting Information

Figure S1 **Total number of simulated RNA-DNA sequence differences.** For both datasets, approximately 600,000 RDDs were generated in each replicate. Differences in the number of each type of RDD reflect underlying variation in base composition throughout the genome, as dataset 2 contains reads originating from intronic regions whereas dataset 1 does not.
(TIF)

Figure S2 **Distribution of coverage for simulated RNA-Seq datasets.** The distribution of coverage, or the total number of reads at a given site, is relatively uniform for dataset 1. In contrast, the distribution of coverage for dataset 2 is skewed right mainly owing to the presence of intronic reads. Approximately 82% of the sites in dataset 2 have a depth of coverage lower or equal to 10x.
(TIF)

Figure S3 **Levels of simulated RNA-DNA sequence differences.** Here we depict the distribution of RDD levels, or the percentage of reads at the sequence difference site that bear the RNA-DNA sequence difference. Because of the discrete nature of RNA-Seq data, the levels of RDDs at sites with relatively low coverage is not uniform as shown by the blue area, which represents sites with coverage less than 10x. For sites with coverage greater than 100x (red area), the density curve of sequence difference levels is fairly uniform except at boundary conditions.
(TIF)

Figure S4 **Sensitivity of RDD detection versus uniqueness of flanking genomic sequence.** Here we show the sensitivity or true positive rate of RDD detection for regions in the genome that are unique (in blue) versus not unique (in red) as determined by BLAT (see Materials and Methods). Sites with fewer than 10 total reads per the simulated RNA-Seq dataset or a RDD level less than 10% per the simulated dataset are removed from consideration.
(TIF)

Figure S5 **Percentage of sites with observed levels that deviate from simulated RDD levels.** Here we calculate the percentage of total sites in each dataset (y-axis) with observed levels that deviate from the simulated RDD level by various degrees (x-axis).
(TIF)

Figure S6 **Effect of requiring RDDs to be identified by multiple aligners on FDR of RDD detection for GSNAP.** Here we depict the false discovery rate of RDD detection using GSNAP under a ‘minimum coverage of 20x, minimum level of 20%, and a minimum of 4 reads bearing the sequence difference’ threshold after requiring various numbers of other aligners to concordantly identify the RDD.
(TIF)

Figure S7 **Effect of requiring RDDs to be identified by multiple aligners on FDR of RDD detection for MapSplice.** Here we depict the false discovery rate of RDD detection using MapSplice under a ‘minimum coverage of 20x, minimum level of 20%, and a minimum of 4 reads bearing the sequence difference’ threshold after requiring various numbers of other aligners to concordantly identify the RDD.
(TIF)

Figure S8 **Effect of requiring RDDs to be identified by multiple aligners on FDR of RDD detection for RUM.** Here we depict the false discovery rate of RDD detection using RUM under a ‘minimum coverage of 20x, minimum level of 20%, and a minimum of 4 reads bearing the sequence difference’ threshold after requiring various numbers of other aligners to concordantly identify the RDD.
(TIF)

Figure S9 **Effect of requiring RDDs to be identified by multiple aligners on FDR of RDD detection for Tophat2.** Here we depict the false discovery rate of RDD detection using Tophat2 under a ‘minimum coverage of 20x, minimum level of 20%, and a minimum of 4 reads bearing the sequence difference’ threshold after requiring various numbers of other aligners to concordantly identify the RDD.
(TIF)

Figure S10 **Percentage of false versus true positives removed using BLAT filter for dataset 1.** Here we depict the percentage of false positives versus true positives that are removed when using the BLAT filter for dataset 1.
(TIF)

Figure S11 **Percentage of false versus true positives removed using BLAT filter for dataset 2.** Here we depict the percentage of false positives versus true positives that are removed when using the BLAT filter for dataset 2.
(TIF)

Figure S12 **Effect of BLAT filter on false discovery rate of RNA-DNA sequence difference detection.** Here we depict the effect of the BLAT filter on the FDR for various aligners and thresholds for identification of sequence differences.
(TIF)

Figure S13 **T-to-G RNA-DNA sequence difference at chr10:102046378.** Here we show an image in the IGV browser [1] of a T-to-G sequence differences at chr10:102046378 in the first replicate of the GM12878 dataset. Each grey bar represents an RNA-Seq read. Mismatches are depicted by colored letters. Black dashes within a read represent a clipped sequence; for reads in the bottom half, the string of colored bases depict clipped portions of the sequence. Clipped portions of alignments represent bases that are not aligned within a local alignment.
(TIF)

Figure S14 **Number of properly aligned bases in reads that overlap RNA-DNA sequences.** Here we depict the number of bases within each read that overlaps an RNA-DNA sequence difference site that are aligned properly. This number excludes bases that contain mismatches or those that are clipped or part of an insertion deletion (indel).
(TIF)

File S1 **Table S1,** Alignment statistics of simulated RNA-Seq datasets. **Table S2,** Summary statistics on distance between neighboring RNA-DNA sequence differences. **Table S3,** Sensitivity of RNA-DNA sequence difference detection versus coverage threshold. **Table S4,** Sensitivity of RDD detection versus the level of sequence difference. **Table S5,** Sensitivity of RNA-DNA sequence difference detection in unique versus non-unique regions as determined by BLAT. **Table S6,** Sensitivity of RDD detection within RepeatMasker regions. **Table S7,** Sensitivity of RDD detection versus proximity to nearby RDDs. **Table S8,** Correlation between observed and simulated levels of RDDs. **Table S9,** Percent of sites with levels where the observed and simulated levels deviate by more than 30% versus the uniqueness of the underlying site as determined by BLAT. **Table S10,** Receiver operating characteristic analysis of RNA-DNA sequence difference detection. **Table S11,** Effect of requiring RDDs to be concordantly identified by multiple aligners on FDR of RDD detection. **Table S12,** Percentage of true versus false positives removed by BLAT filter. **Table S13,** Effect of BLAT filter on false discovery rate of RDD detection. **Table S14,** Effect of removing RNA-DNA sequence differences in pseudogenes on the false discovery rate of sequence difference detection. **Table S15,** Effect of removing RDDs near exon junctions on the false discovery rate of sequence difference detection. **Table S16,** Percentage of true versus false positives removed by requiring concordance with at least one other aligner, BLAT filter, pseudogene filter, and removal of intronic sites within 6 bp of exon junctions used in conjunction. **Table S17,** Combined effect of requiring concordance with at least one other aligner, BLAT filter, pseudogene filter, and removal of intronic sites within 6 bp of exon junctions on the false discovery rate of sequence difference detection. **Table S18,** Alignment statistics for GM12878 RNA-Seq dataset. **Table S19,** RNA-DNA sequence differences found in GM12878. **Table S20,** Overlap of RNA-DNA sequence differences found in GM12878 with other published studies.
(DOCX)
