# Supplementary figures and images for: Detection Theory in Identification of RNA-DNA Sequence Differences Using RNA-Sequencing
Source: PLoS One. 2014 Nov 14;9(11):e112040. doi: 10.1371/journal.pone.0112040 (PMC4232354; doi:10.1371/journal.pone.0112040)

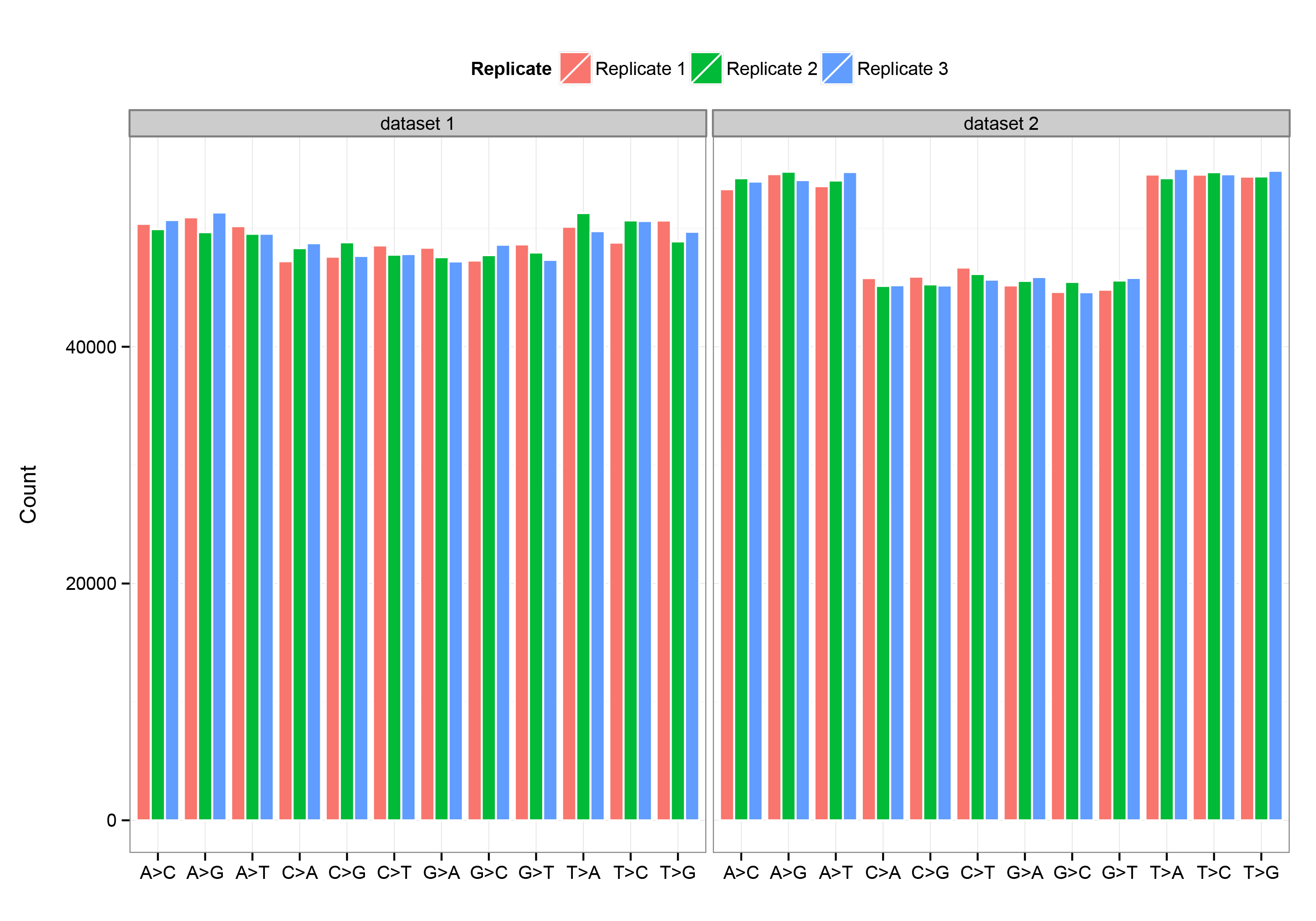

Supplement: Figure S1 — Total number of simulated RNA-DNA sequence differences. For both datasets, approximately 600,000 RDDs were generated in each replicate. Differences in the number of each type of RDD reflect underlying variation in base composition throughout the genome, as dataset 2 contains reads originating from intronic regions whereas dataset 1 does not. (TIF) [file pone.0112040.s001.tif]

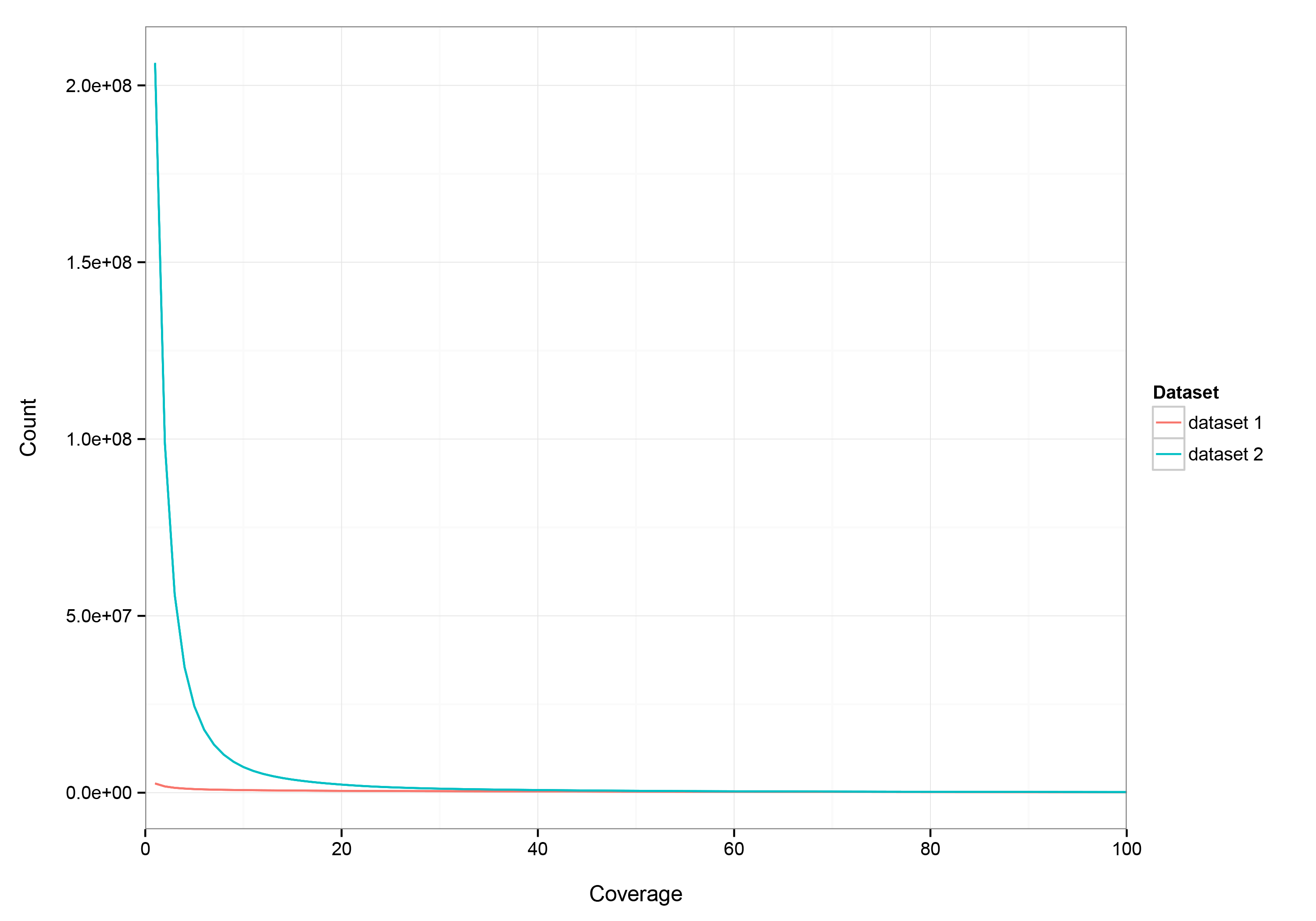

Supplement: Figure S2 — Distribution of coverage for simulated RNA-Seq datasets. The distribution of coverage, or the total number of reads at a given site, is relatively uniform for dataset 1. In contrast, the distribution of coverage for dataset 2 is skewed right mainly owing to the presence of intronic reads. Approximately 82% of the sites in dataset 2 have a depth of coverage lower or equal to 10x. (TIF) [file pone.0112040.s002.tif]

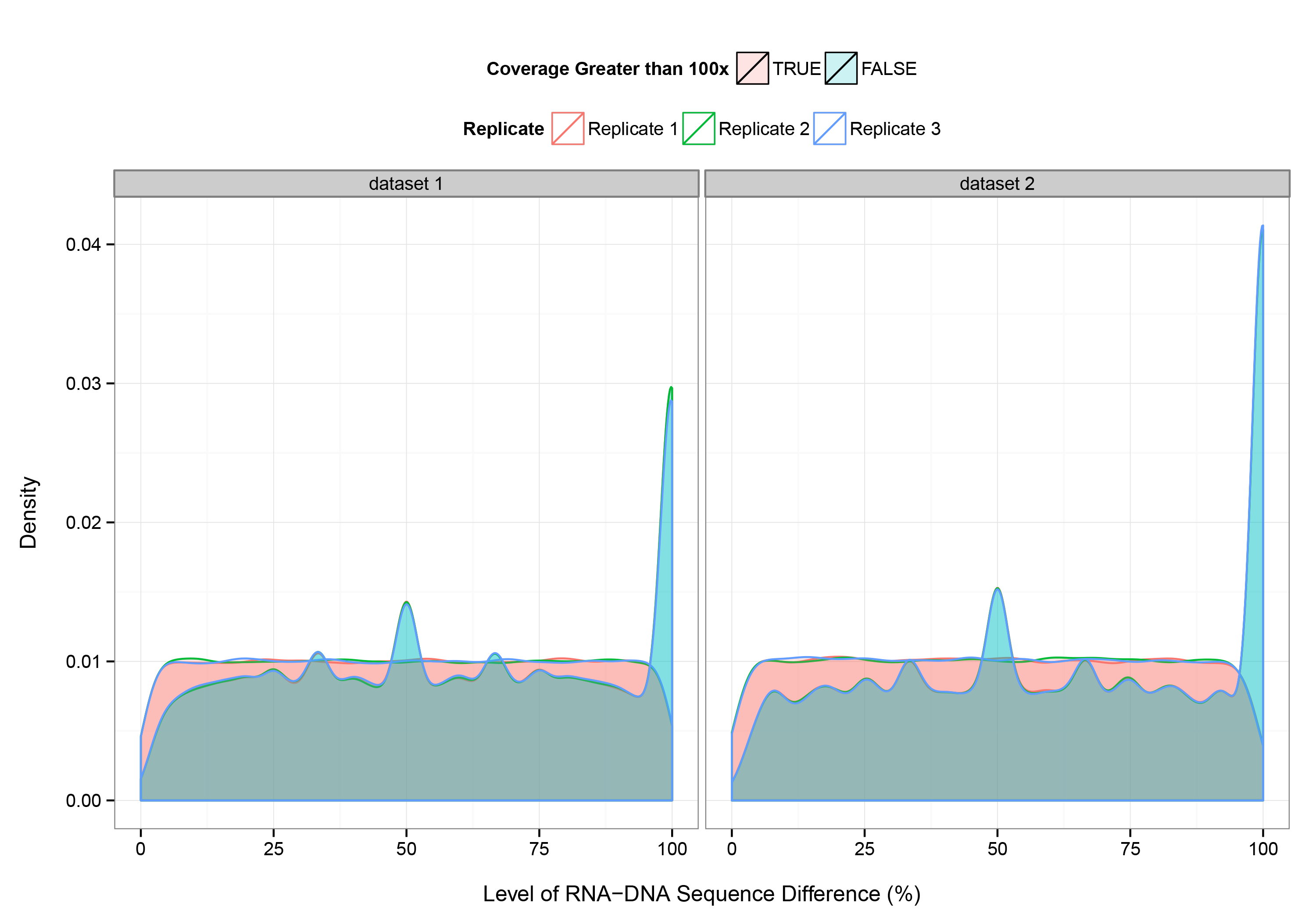

Supplement: Figure S3 — Levels of simulated RNA-DNA sequence differences. Here we depict the distribution of RDD levels, or the percentage of reads at the sequence difference site that bear the RNA-DNA sequence difference. Because of the discrete nature of RNA-Seq data, the levels of RDDs at sites with relatively low coverage is not uniform as shown by the blue area, which represents sites with coverage less than 10x. For sites with coverage greater than 100x (red area), the density curve of sequence difference levels is fairly uniform except at boundary conditions. (TIF) [file pone.0112040.s003.tif]

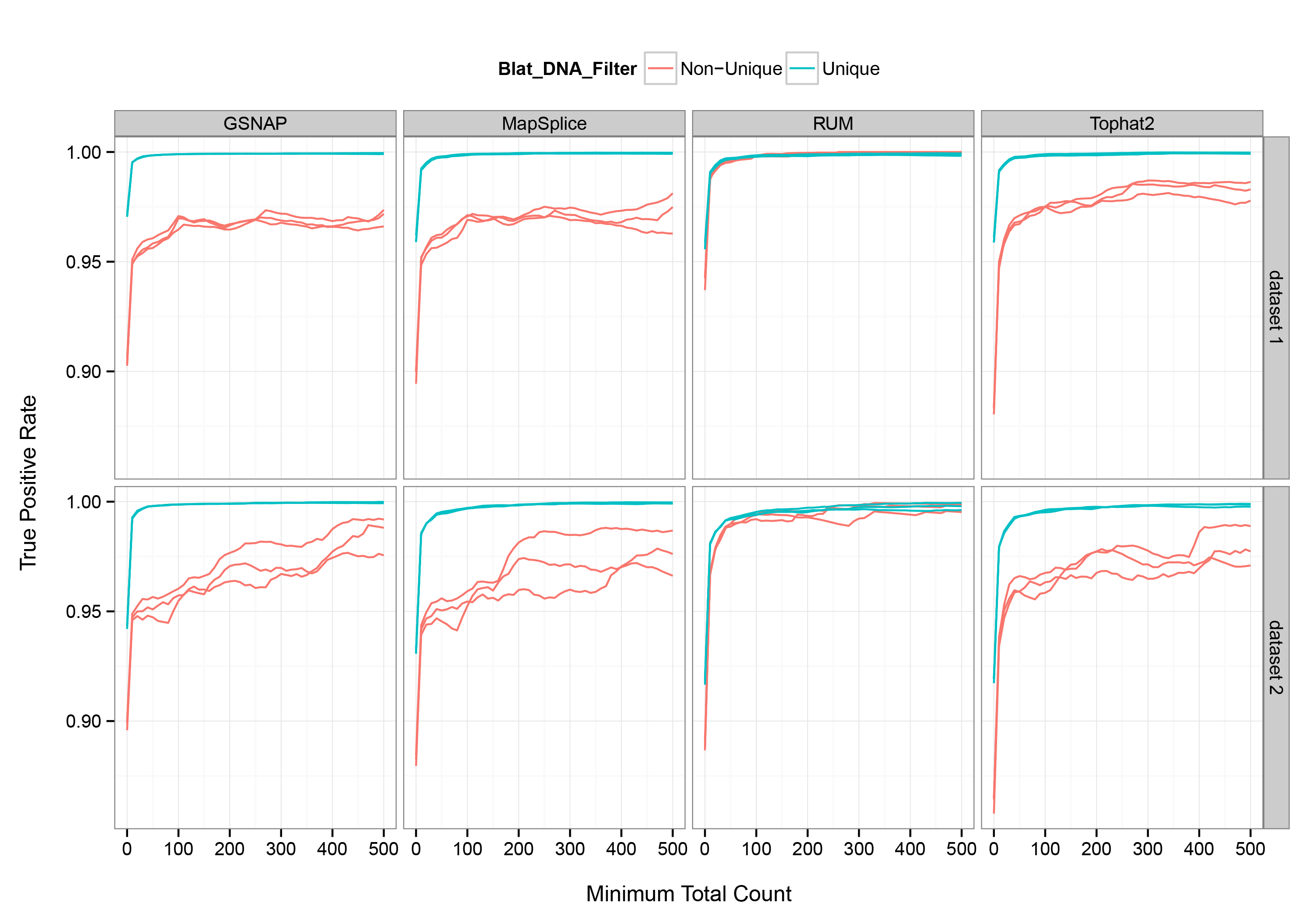

Supplement: Figure S4 — Sensitivity of RDD detection versus uniqueness of flanking genomic sequence. Here we show the sensitivity or true positive rate of RDD detection for regions in the genome that are unique (in blue) versus not unique (in red) as determined by BLAT (see Materials and Methods). Sites with fewer than 10 total reads per the simulated RNA-Seq dataset or a RDD level less than 10% per the simulated dataset are removed from consideration. (TIF) [file pone.0112040.s004.tif]

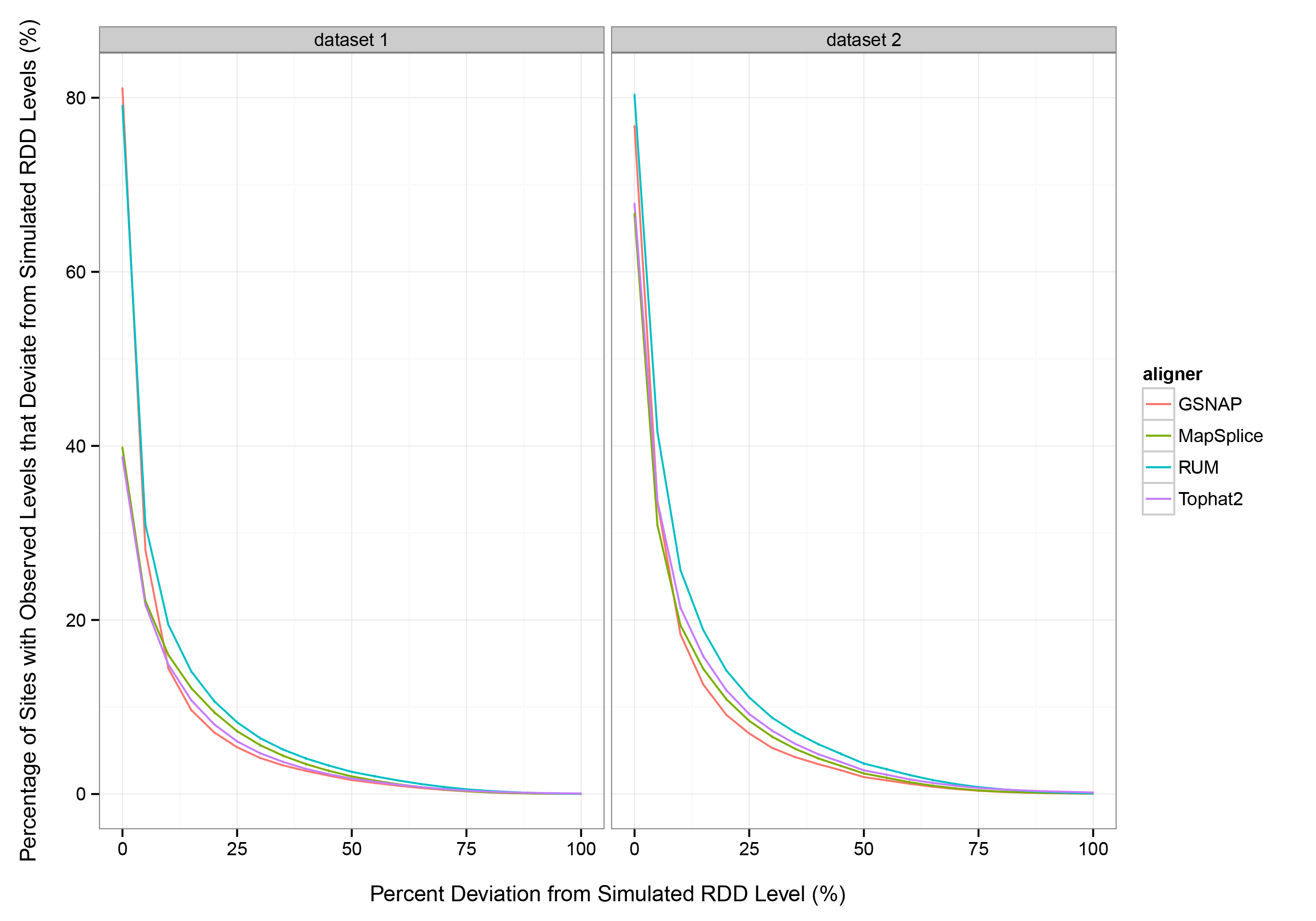

Supplement: Figure S5 — Percentage of sites with observed levels that deviate from simulated RDD levels. Here we calculate the percentage of total sites in each dataset (y-axis) with observed levels that deviate from the simulated RDD level by various degrees (x-axis). (TIF) [file pone.0112040.s005.tif]

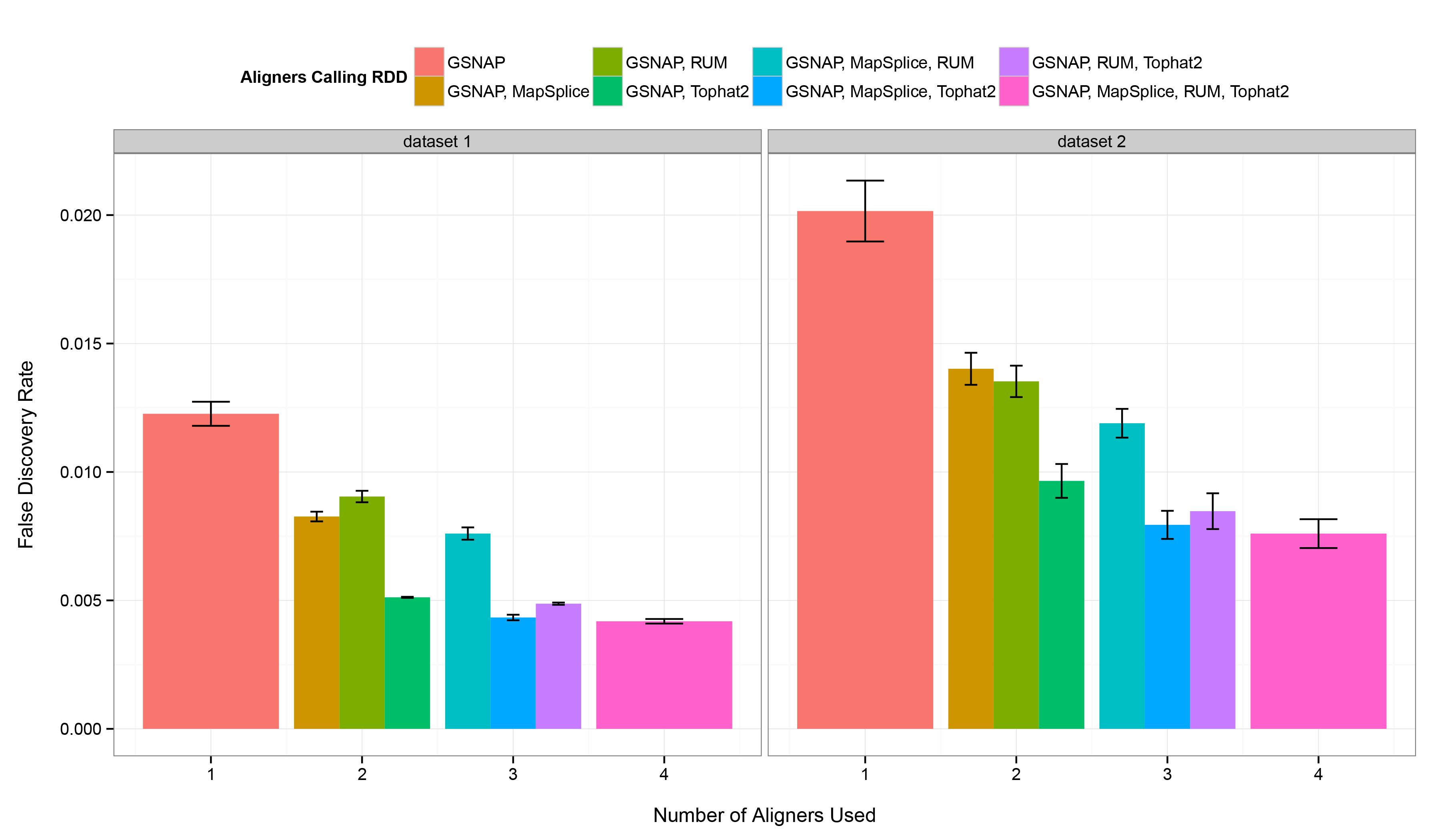

Supplement: Figure S6 — Effect of requiring RDDs to be identified by multiple aligners on FDR of RDD detection for GSNAP. Here we depict the false discovery rate of RDD detection using GSNAP under a ‘minimum coverage of 20x, minimum level of 20%, and a minimum of 4 reads bearing the sequence difference’ threshold after requiring various numbers of other aligners to concordantly identify the RDD. (TIF) [file pone.0112040.s006.tif]

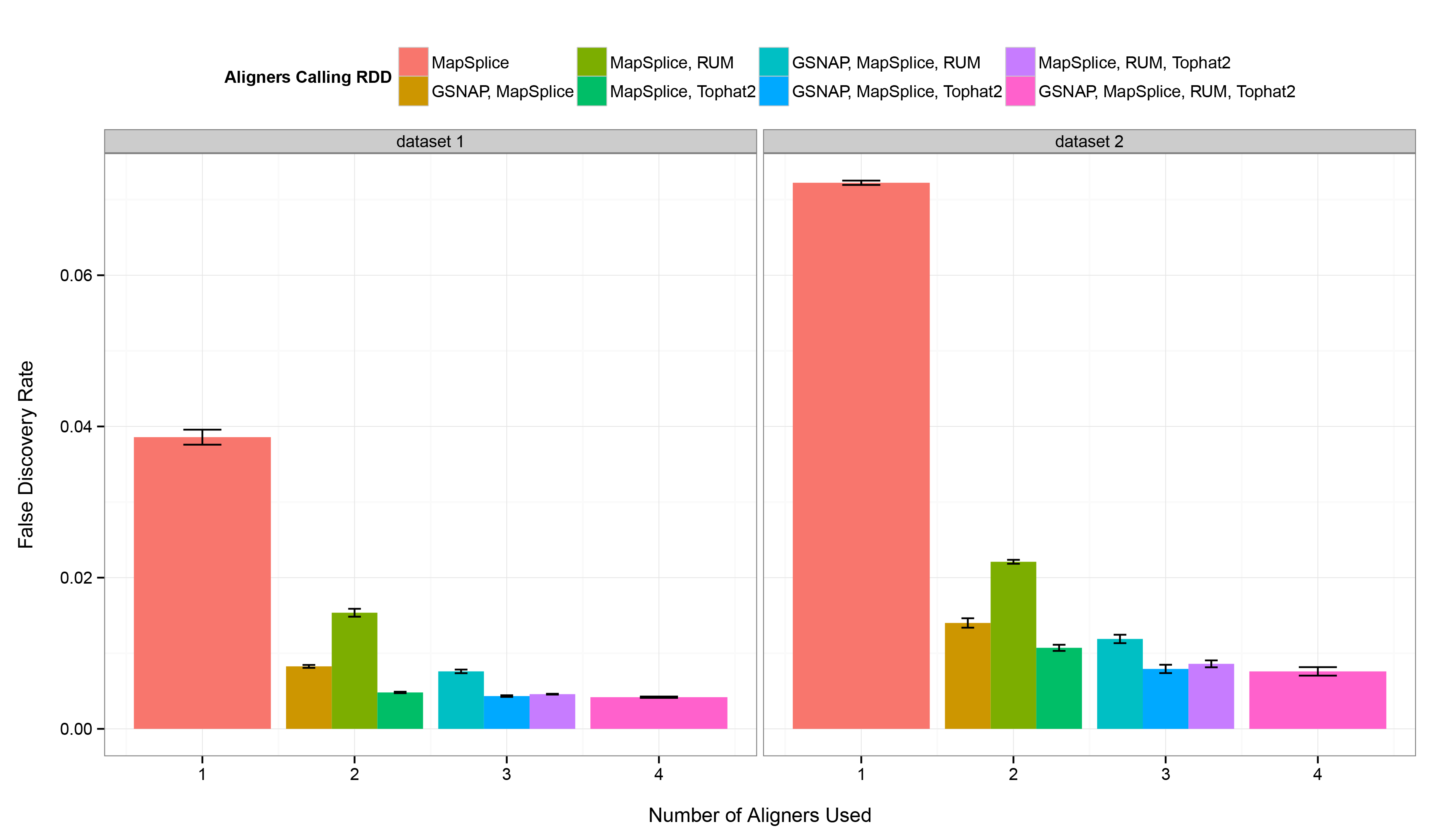

Supplement: Figure S7 — Effect of requiring RDDs to be identified by multiple aligners on FDR of RDD detection for MapSplice. Here we depict the false discovery rate of RDD detection using MapSplice under a ‘minimum coverage of 20x, minimum level of 20%, and a minimum of 4 reads bearing the sequence difference’ threshold after requiring various numbers of other aligners to concordantly identify the RDD. (TIF) [file pone.0112040.s007.tif]

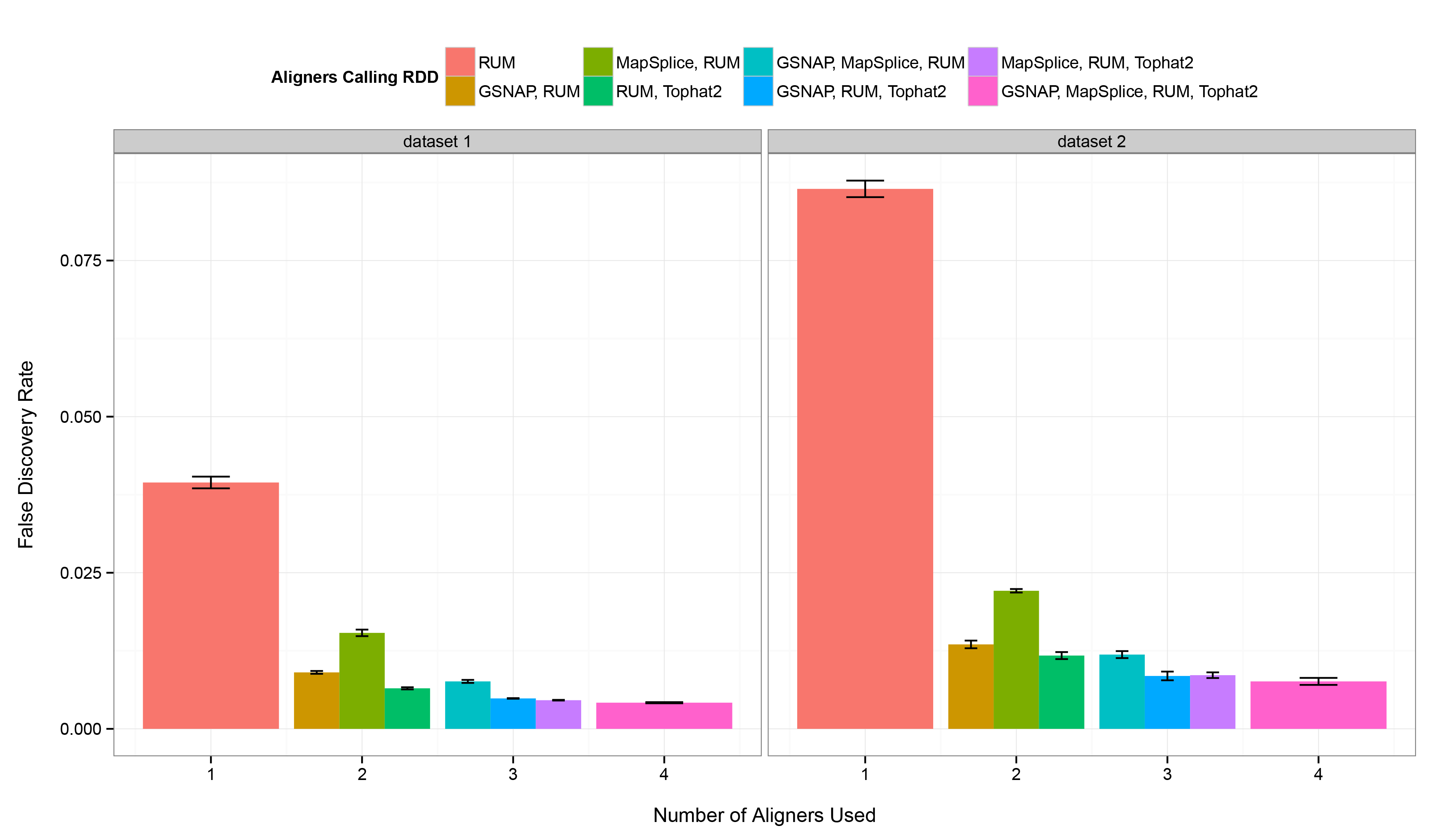

Supplement: Figure S8 — Effect of requiring RDDs to be identified by multiple aligners on FDR of RDD detection for RUM. Here we depict the false discovery rate of RDD detection using RUM under a ‘minimum coverage of 20x, minimum level of 20%, and a minimum of 4 reads bearing the sequence difference’ threshold after requiring various numbers of other aligners to concordantly identify the RDD. (TIF) [file pone.0112040.s008.tif]

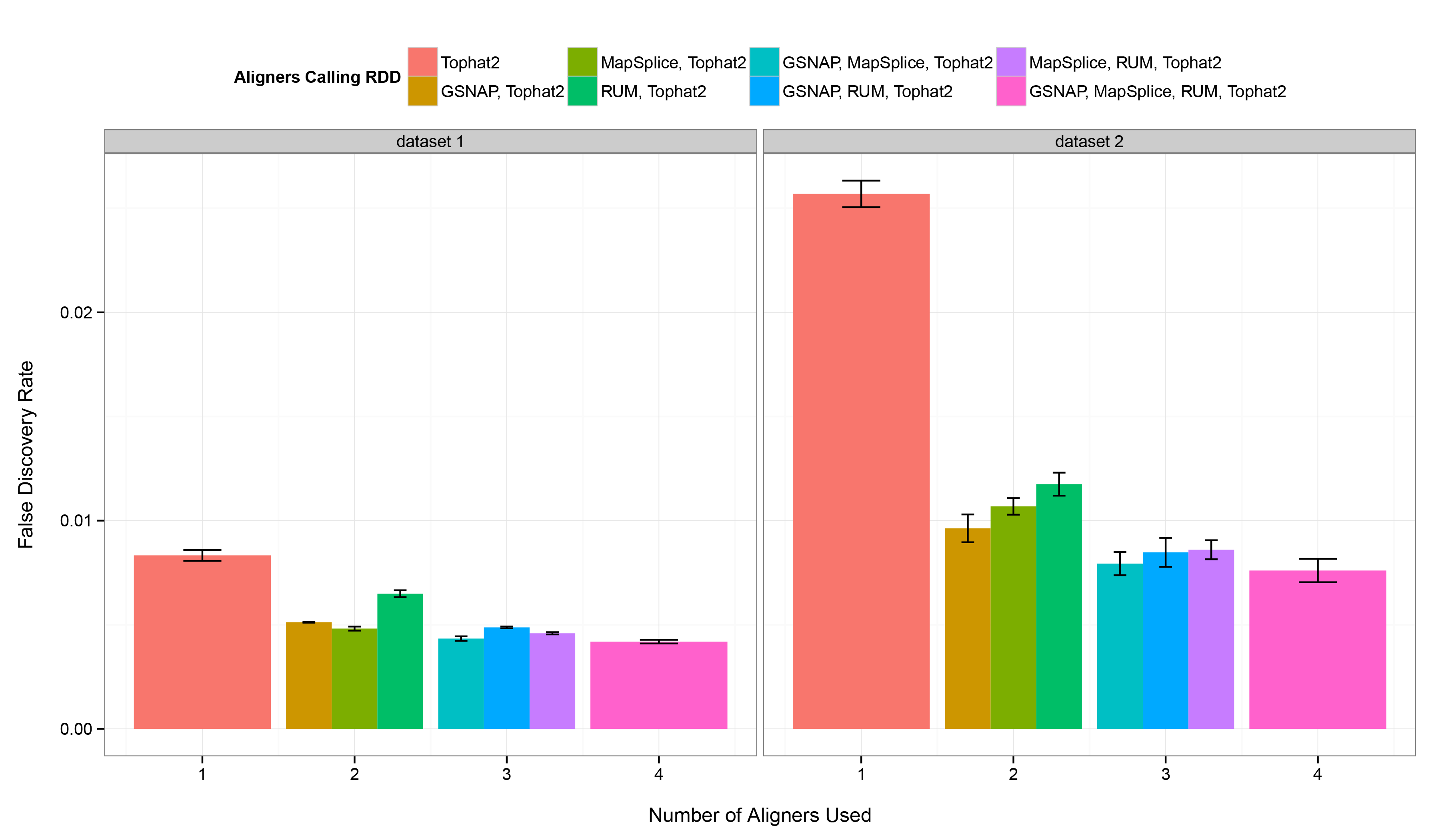

Supplement: Figure S9 — Effect of requiring RDDs to be identified by multiple aligners on FDR of RDD detection for Tophat2. Here we depict the false discovery rate of RDD detection using Tophat2 under a ‘minimum coverage of 20x, minimum level of 20%, and a minimum of 4 reads bearing the sequence difference’ threshold after requiring various numbers of other aligners to concordantly identify the RDD. (TIF) [file pone.0112040.s009.tif]

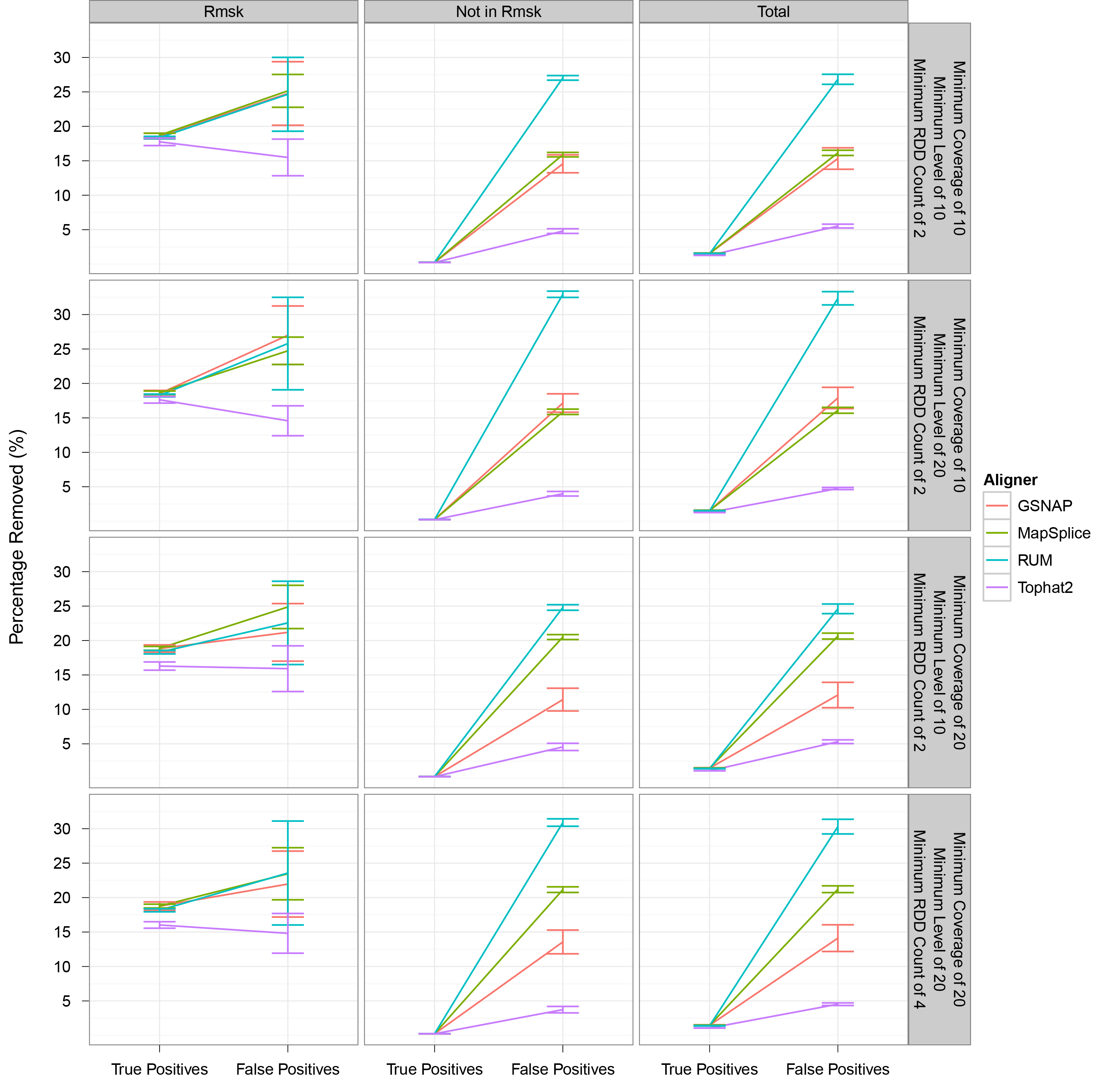

Supplement: Figure S10 — Percentage of false versus true positives removed using BLAT filter for dataset 1. Here we depict the percentage of false positives versus true positives that are removed when using the BLAT filter for dataset 1. (TIF) [file pone.0112040.s010.tif]

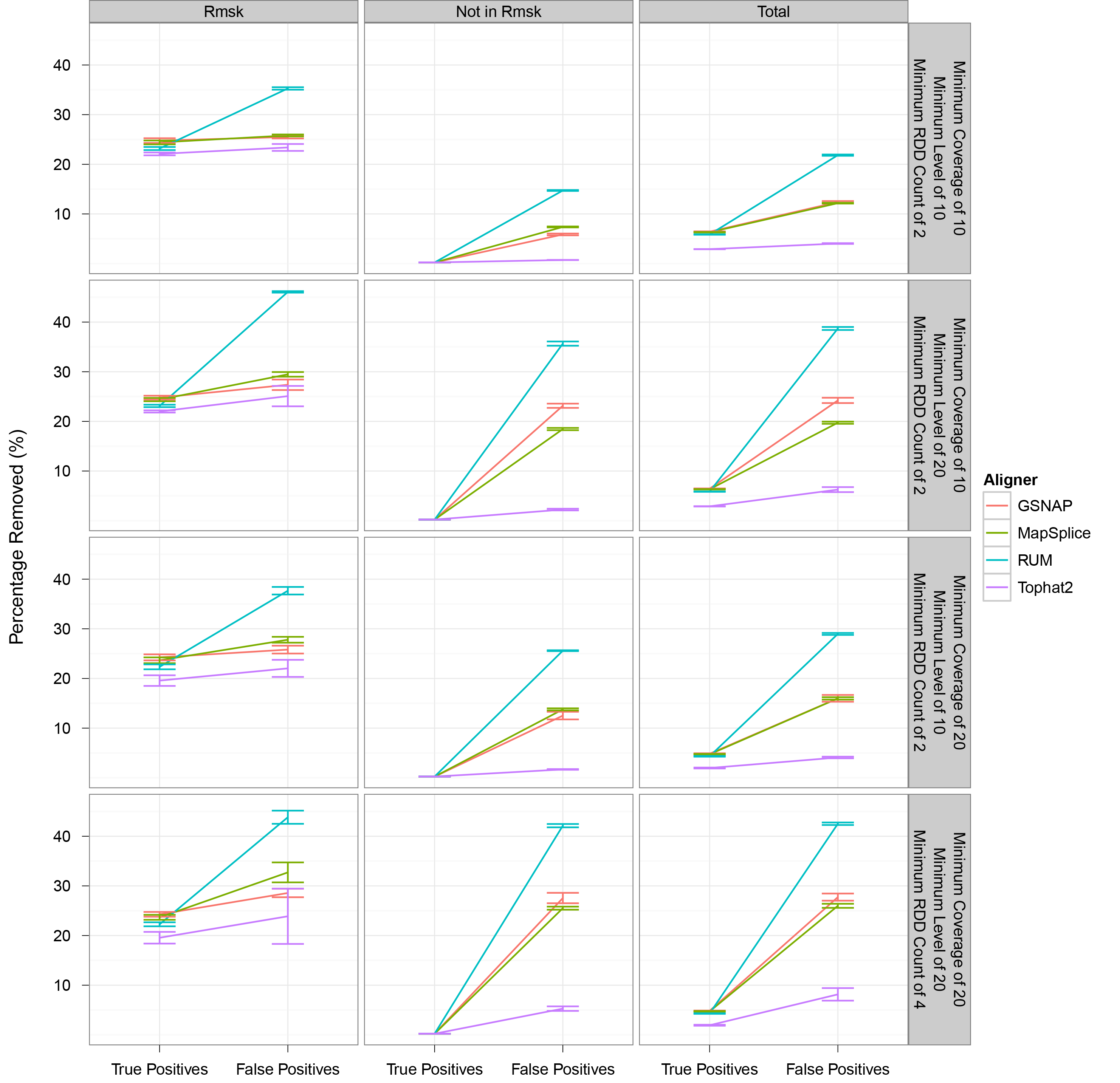

Supplement: Figure S11 — Percentage of false versus true positives removed using BLAT filter for dataset 2. Here we depict the percentage of false positives versus true positives that are removed when using the BLAT filter for dataset 2. (TIF) [file pone.0112040.s011.tif]

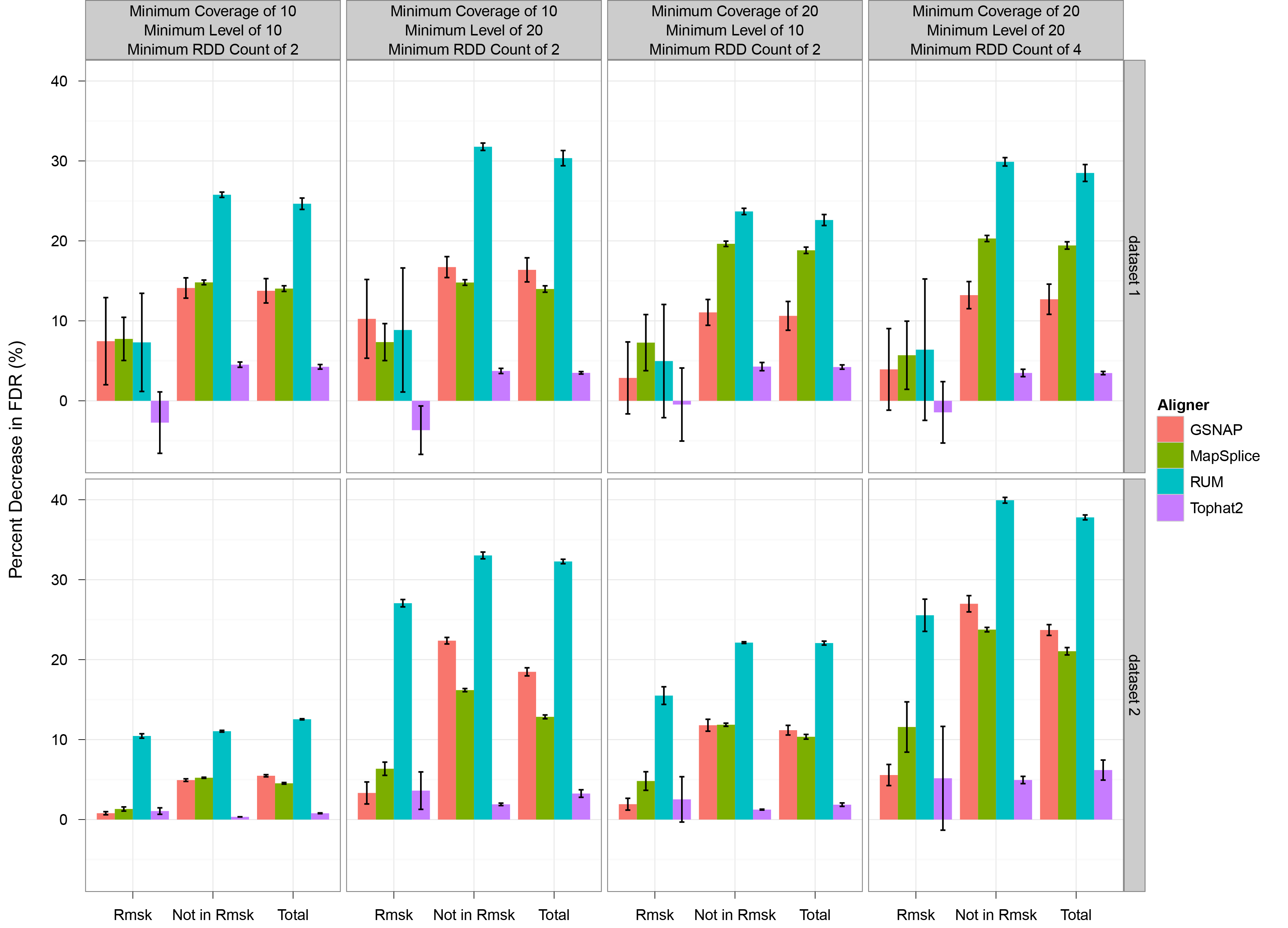

Supplement: Figure S12 — Effect of BLAT filter on false discovery rate of RNA-DNA sequence difference detection. Here we depict the effect of the BLAT filter on the FDR for various aligners and thresholds for identification of sequence differences. (TIF) [file pone.0112040.s012.tif]

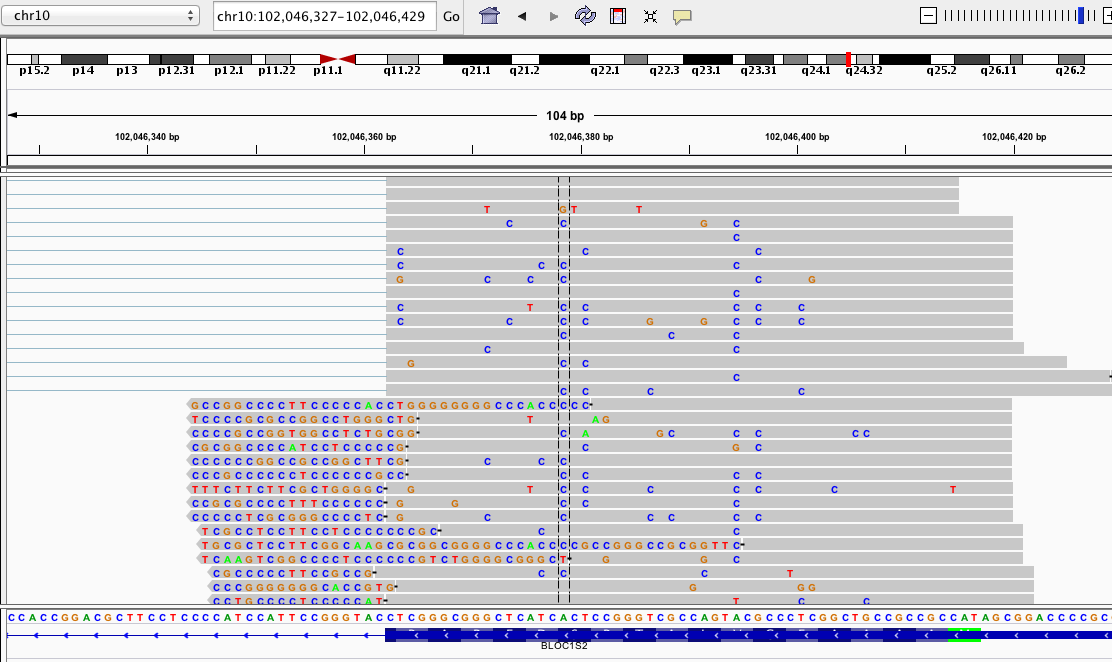

Supplement: Figure S13 — T-to-G RNA-DNA sequence difference at chr10:102046378. Here we show an image in the IGV browser [1] of a T-to-G sequence differences at chr10:102046378 in the first replicate of the GM12878 dataset. Each grey bar represents an RNA-Seq read. Mismatches are depicted by colored letters. Black dashes within a read represent a clipped sequence; for reads in the bottom half, the string of colored bases depict clipped portions of the sequence. Clipped portions of alignments represent bases that are not aligned within a local alignment. (TIF) [file pone.0112040.s013.tif]

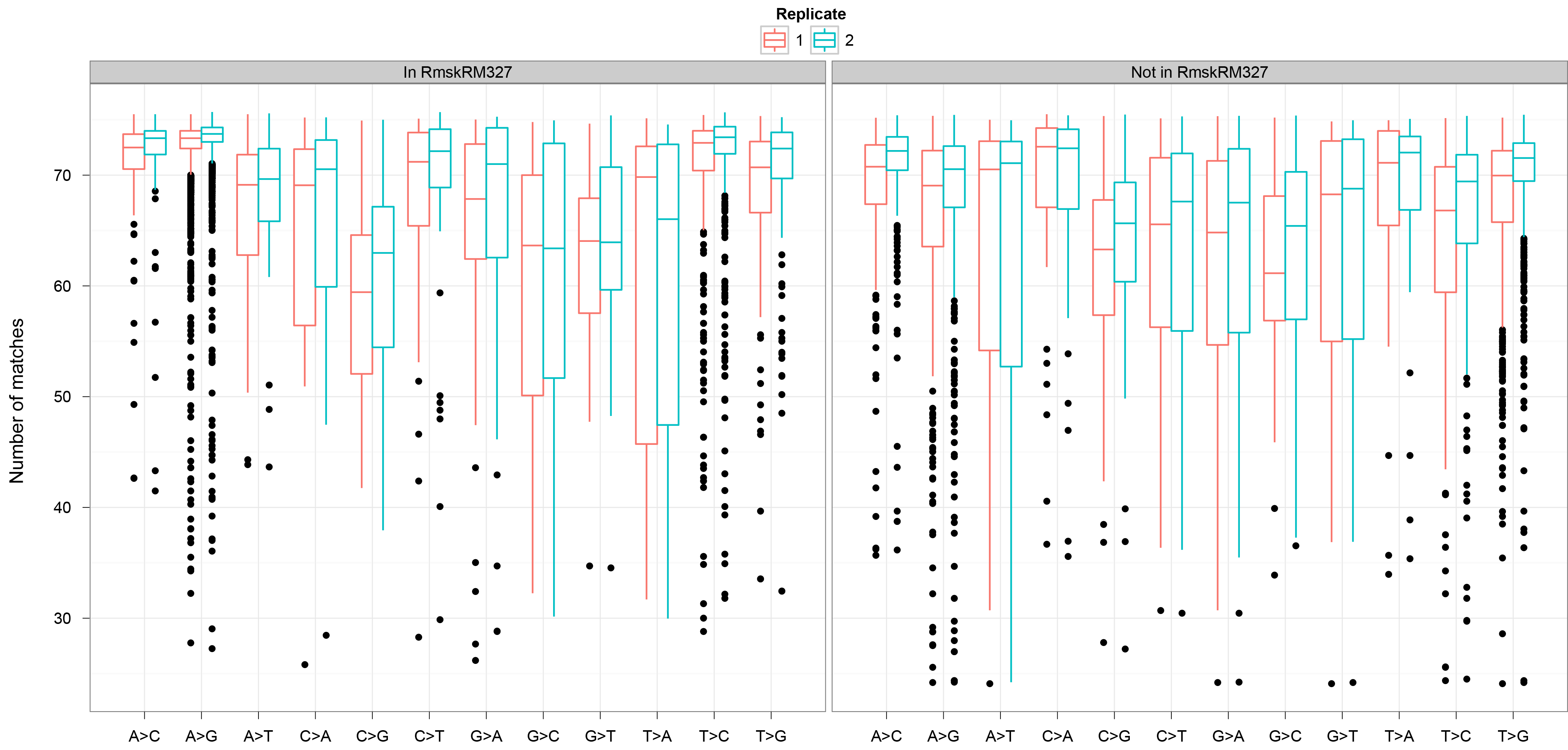

Supplement: Figure S14 — Number of properly aligned bases in reads that overlap RNA-DNA sequences. Here we depict the number of bases within each read that overlaps an RNA-DNA sequence difference site that are aligned properly. This number excludes bases that contain mismatches or those that are clipped or part of an insertion deletion (indel). (TIF) [file pone.0112040.s014.tif]
