# Supplementary material for: Detection Theory in Identification of RNA-DNA Sequence Differences Using RNA-Sequencing
Source: PLoS One. 2014 Nov 14;9(11):e112040. doi: 10.1371/journal.pone.0112040 (PMC4232354; doi:10.1371/journal.pone.0112040)
Supplement: File S1 — Table S1, Alignment statistics of simulated RNA-Seq datasets. Table S2, Summary statistics on distance between neighboring RNA-DNA sequence differences. Table S3, Sensitivity of RNA-DNA sequence difference detection versus coverage threshold. Table S4, Sensitivity of RDD detection versus the level of sequence difference. Table S5, Sensitivity of RNA-DNA sequence difference detection in unique versus non-unique regions as determined by BLAT. Table S6, Sensitivity of RDD detection within RepeatMasker regions. Table S7, Sensitivity of RDD detection versus proximity to nearby RDDs. Table S8, Correlation between observed and simulated levels of RDDs. Table S9, Percent of sites with levels where the observed and simulated levels deviate by more than 30% versus the uniqueness of the underlying site as determined by BLAT. Table S10, Receiver operating characteristic analysis of RNA-DNA sequence difference detection. Table S11, Effect of requiring RDDs to be concordantly identified by multiple aligners on FDR of RDD detection. Table S12, Percentage of true versus false positives removed by BLAT filter. Table S13, Effect of BLAT filter on false discovery rate of RDD detection. Table S14, Effect of removing RNA-DNA sequence differences in pseudogenes on the false discovery rate of sequence difference detection. Table S15, Effect of removing RDDs near exon junctions on the false discovery rate of sequence difference detection. Table S16, Percentage of true versus false positives removed by requiring concordance with at least one other aligner, BLAT filter, pseudogene filter, and removal of intronic sites within 6 bp of exon junctions used in conjunction. Table S17, Combined effect of requiring concordance with at least one other aligner, BLAT filter, pseudogene filter, and removal of intronic sites within 6 bp of exon junctions on the false discovery rate of sequence difference detection. Table S18, Alignment statistics for GM12878 RNA-Seq dataset. Table S19, RNA-DNA sequence d [file pone.0112040.s015.docx]

**SUPPLEMENTARY INFORMATION**

**Detection theory in identification of RNA-DNA sequence differences using RNA-Sequencing**

Jonathan M. Toung^1^, Nicholas Lahens^1^, John Hogenesch^2,3,5^ and Gregory Grant^2,3,4^

^1^Genomics and Computational Biology Graduate Program, ^2^Institute for Biomedical Informatics, ^3^Institute for Translational Medicine and Therapeutics, ^4^Department of Genetics, ^5^Department of Pharmacology, University of Pennsylvania School of Medicine, Philadelphia, PA 19104, USA.

# Table S1. Alignment statistics of simulated RNA-Seq datasets.

| **Dataset** | **Aligner** | **Statistic** | **Value** |
| --- | --- | --- | --- |
| dataset 1 | GSNAP | Number of Read Pairs Aligned | 49706613 ± 5861 |
|  |  | Percentage of Read Pairs Aligned | 99.41 ± 1.17E-2% |
|  |  | Number of Read Pairs Aligned Uniquely | 49057838 ± 5548 |
|  |  | Percentage of Read Pairs Aligned Uniquely | 98.69 ± 1.19E-3% |
| dataset 1 | MapSplice | Number of Read Pairs Aligned | 49614873 ± 3479 |
|  |  | Percentage of Read Pairs Aligned | 99.23 ± 6.96E-3% |
|  |  | Number of Read Pairs Aligned Uniquely | 49272568 ± 2205 |
|  |  | Percentage of Read Pairs Aligned Uniquely | 99.31 ± 9.35E-3% |
| dataset 1 | RUM | Number of Read Pairs Aligned | 49338154 ± 5464 |
|  |  | Percentage of Read Pairs Aligned | 98.68 ± 1.09E-2% |
|  |  | Number of Read Pairs Aligned Uniquely | 48192283 ± 5415 |
|  |  | Percentage of Read Pairs Aligned Uniquely | 97.68 ± 3.92E-3% |
| dataset 1 | Tophat2 | Number of Read Pairs Aligned | 49144986 ± 6939 |
|  |  | Percentage of Read Pairs Aligned | 98.3 ± 1.32E-2% |
|  |  | Number of Read Pairs Aligned Uniquely | 43744298 ± 5756 |
|  |  | Percentage of Read Pairs Aligned Uniquely | 89.01 ± 8.45E-3% |
| dataset 2 | GSNAP | Number of Read Pairs Aligned | 49790494 ± 3376 |
|  |  | Percentage of Read Pairs Aligned | 99.58 ± 6.75E-3% |
|  |  | Number of Read Pairs Aligned Uniquely | 49095402 ± 4246 |
|  |  | Percentage of Read Pairs Aligned Uniquely | 98.6 ± 3.39E-3% |
| dataset 2 | MapSplice | Number of Read Pairs Aligned | 49626996 ± 1541 |
|  |  | Percentage of Read Pairs Aligned | 99.25 ± 3.08E-3% |
|  |  | Number of Read Pairs Aligned Uniquely | 49047103 ± 1172 |
|  |  | Percentage of Read Pairs Aligned Uniquely | 98.83 ± 7.43E-4% |
| dataset 2 | RUM | Number of Read Pairs Aligned | 47975774 ± 34932 |
|  |  | Percentage of Read Pairs Aligned | 95.95 ± 6.99E-2% |
|  |  | Number of Read Pairs Aligned Uniquely | 46820152 ± 35128 |
|  |  | Percentage of Read Pairs Aligned Uniquely | 97.59 ± 6.21E-3% |
| dataset 2 | Tophat2 | Number of Read Pairs Aligned | 47763421 ± 77673 |
|  |  | Percentage of Read Pairs Aligned | 95.61 ± 1.60E-1% |
|  |  | Number of Read Pairs Aligned Uniquely | 43072870 ± 73773 |
|  |  | Percentage of Read Pairs Aligned Uniquely | 90.18 ± 1.49E-2% |

# Table S2. Summary statistics on distance between neighboring RNA-DNA sequence differences.

| **Dataset** | **In Cluster** | **Number of Sites** | **Distance to nearest RNA-DNA sequence difference*** | | | | | |
| --- | --- | --- | --- | --- | --- | --- | --- | --- |
|  |  |  | **Minimum** | **1st Quartile** | **Median** | **Mean** | **3rd Quartile** | **Maximum** |
| dataset 1 | TRUE | 219239 | 1 | 1 | 3 | 10 | 5 | 155900 |
| dataset 1 | FALSE | 369416 | 1 | 23 | 58 | 815 | 146 | 1322700 |
| dataset 1 | TOTAL | 588654 | 1 | 4 | 19 | 515 | 82 | 1322700 |
| dataset 2 | TRUE | 222509 | 1 | 1 | 3 | 10 | 5 | 112347 |
| dataset 2 | FALSE | 376504 | 1 | 75 | 225 | 1565 | 869 | 2317667 |
| dataset 2 | TOTAL | 599013 | 1 | 4 | 59 | 987 | 353 | 2317667 |

*Note: Each statistic is averaged across the 3 replicates.

# Table S3. Sensitivity of RNA-DNA sequence difference detection versus coverage threshold.

| **Dataset** | **Aligner** | **Minimum Coverage or Total Count§** | **Sensitivity (%)** |
| --- | --- | --- | --- |
| dataset 1 | GSNAP | 0 | 96.32 ± 6.19E-2 |
| dataset 1 | MapSplice | 0 | 95.30 ± 1.61E-1 |
| dataset 1 | RUM | 0 | 95.36 ± 1.63E-1 |
| dataset 1 | Tophat2 | 0 | 95.04 ± 1.38E-1 |
| dataset 1 | GSNAP | 10 | 98.73 ± 1.23E-2 |
| dataset 1 | MapSplice | 10 | 98.41 ± 3.22E-2 |
| dataset 1 | RUM | 10 | 98.52 ± 3.40E-2 |
| dataset 1 | Tophat2 | 10 | 98.24 ± 3.00E-2 |
| dataset 1 | GSNAP | 50 | 99.27 ± 2.57E-2 |
| dataset 1 | MapSplice | 50 | 99.16 ± 3.84E-2 |
| dataset 1 | RUM | 50 | 99.34 ± 3.62E-2 |
| dataset 1 | Tophat2 | 50 | 99.13 ± 5.09E-2 |
| dataset 1 | GSNAP | 100 | 99.49 ± 4.42E-2 |
| dataset 1 | MapSplice | 100 | 99.44 ± 2.99E-2 |
| dataset 1 | RUM | 100 | 99.57 ± 4.65E-2 |
| dataset 1 | Tophat2 | 100 | 99.40 ± 5.56E-2 |
| dataset 2 | GSNAP | 0 | 93.54 ± 1.04E-1 |
| dataset 2 | MapSplice | 0 | 92.34 ± 1.16E-1 |
| dataset 2 | RUM | 0 | 91.12 ± 1.33E-1 |
| dataset 2 | Tophat2 | 0 | 90.84 ± 1.54E-1 |
| dataset 2 | GSNAP | 10 | 98.03 ± 4.48E-2 |
| dataset 2 | MapSplice | 10 | 97.30 ± 1.43E-2 |
| dataset 2 | RUM | 10 | 96.99 ± 2.61E-2 |
| dataset 2 | Tophat2 | 10 | 96.49 ± 5.26E-2 |
| dataset 2 | GSNAP | 50 | 98.97 ± 4.44E-2 |
| dataset 2 | MapSplice | 50 | 98.56 ± 3.39E-2 |
| dataset 2 | RUM | 50 | 98.60 ± 2.80E-2 |
| dataset 2 | Tophat2 | 50 | 98.35 ± 4.16E-2 |
| dataset 2 | GSNAP | 100 | 99.21 ± 2.18E-2 |
| dataset 2 | MapSplice | 100 | 98.93 ± 7.61E-3 |
| dataset 2 | RUM | 100 | 98.99 ± 5.08E-2 |
| dataset 2 | Tophat2 | 100 | 98.78 ± 8.05E-2 |

*Note: An RNA-DNA sequence difference is considered properly identified if a minimum of 1 read bearing the sequence difference is present per the aligner.

§ The threshold on the minimum coverage applies to the true or simulated coverage at the site of the underlying RNA-DNA sequence difference per the simulated RNA-Seq dataset, not the observed coverage per the aligner.

# Table S4. Sensitivity of RDD detection versus the level of sequence difference.

| **Dataset** | **Aligner** | **RDD Level** | **Sensitivity (%)** |
| --- | --- | --- | --- |
| dataset 1 | GSNAP | 0-10% | 94.87 ± 4.53E-2 |
| dataset 1 | MapSplice | 0-10% | 94.15 ± 1.07E-1 |
| dataset 1 | RUM | 0-10% | 93.26 ± 8.22E-2 |
| dataset 1 | Tophat2 | 0-10% | 93.05 ± 1.54E-1 |
| dataset 1 | GSNAP | 10-20% | 98.12 ± 4.47E-2 |
| dataset 1 | MapSplice | 10-20% | 97.40 ± 1.23E-1 |
| dataset 1 | RUM | 10-20% | 97.41 ± 6.71E-2 |
| dataset 1 | Tophat2 | 10-20% | 96.91 ± 9.03E-2 |
| dataset 1 | GSNAP | 20-30% | 98.84 ± 5.89E-2 |
| dataset 1 | MapSplice | 20-30% | 98.29 ± 4.58E-2 |
| dataset 1 | RUM | 20-30% | 98.50 ± 3.42E-2 |
| dataset 1 | Tophat2 | 20-30% | 98.07 ± 3.54E-2 |
| dataset 1 | GSNAP | 30-40% | 99.13 ± 2.37E-2 |
| dataset 1 | MapSplice | 30-40% | 98.72 ± 3.47E-2 |
| dataset 1 | RUM | 30-40% | 98.90 ± 3.85E-2 |
| dataset 1 | Tophat2 | 30-40% | 98.61 ± 5.32E-2 |
| dataset 1 | GSNAP | 40-50% | 99.21 ± 5.60E-2 |
| dataset 1 | MapSplice | 40-50% | 98.94 ± 4.55E-2 |
| dataset 1 | RUM | 40-50% | 99.14 ± 5.90E-2 |
| dataset 1 | Tophat2 | 40-50% | 98.85 ± 2.70E-2 |
| dataset 1 | GSNAP | 50-60% | 99.24 ± 3.32E-2 |
| dataset 1 | MapSplice | 50-60% | 99.02 ± 4.26E-2 |
| dataset 1 | RUM | 50-60% | 99.27 ± 2.38E-2 |
| dataset 1 | Tophat2 | 50-60% | 99.01 ± 3.78E-2 |
| dataset 1 | GSNAP | 60-70% | 99.34 ± 2.82E-2 |
| dataset 1 | MapSplice | 60-70% | 99.21 ± 7.53E-3 |
| dataset 1 | RUM | 60-70% | 99.45 ± 5.92E-3 |
| dataset 1 | Tophat2 | 60-70% | 99.23 ± 1.23E-2 |
| dataset 1 | GSNAP | 70-80% | 99.35 ± 3.65E-2 |
| dataset 1 | MapSplice | 70-80% | 99.23 ± 1.46E-2 |
| dataset 1 | RUM | 70-80% | 99.50 ± 6.68E-2 |
| dataset 1 | Tophat2 | 70-80% | 99.25 ± 3.85E-2 |
| dataset 1 | GSNAP | 80-90% | 99.36 ± 2.56E-2 |
| dataset 1 | MapSplice | 80-90% | 99.29 ± 5.75E-2 |
| dataset 1 | RUM | 80-90% | 99.52 ± 2.19E-2 |
| dataset 1 | Tophat2 | 80-90% | 99.34 ± 1.38E-2 |
| dataset 1 | GSNAP | 90-100% | 99.32 ± 7.04E-2 |
| dataset 1 | MapSplice | 90-100% | 99.24 ± 4.39E-2 |
| dataset 1 | RUM | 90-100% | 99.48 ± 3.76E-2 |
| dataset 1 | Tophat2 | 90-100% | 99.29 ± 3.84E-2 |
| dataset 2 | GSNAP | 0-10% | 91.03 ± 6.16E-2 |
| dataset 2 | MapSplice | 0-10% | 89.88 ± 1.78E-1 |
| dataset 2 | RUM | 0-10% | 86.87 ± 8.28E-2 |
| dataset 2 | Tophat2 | 0-10% | 86.67 ± 1.43E-1 |
| dataset 2 | GSNAP | 10-20% | 97.00 ± 8.32E-2 |
| dataset 2 | MapSplice | 10-20% | 95.46 ± 1.29E-1 |
| dataset 2 | RUM | 10-20% | 94.72 ± 6.53E-2 |
| dataset 2 | Tophat2 | 10-20% | 93.68 ± 7.05E-2 |
| dataset 2 | GSNAP | 20-30% | 98.16 ± 1.02E-1 |
| dataset 2 | MapSplice | 20-30% | 96.98 ± 1.41E-1 |
| dataset 2 | RUM | 20-30% | 96.65 ± 6.89E-2 |
| dataset 2 | Tophat2 | 20-30% | 95.79 ± 7.86E-2 |
| dataset 2 | GSNAP | 30-40% | 98.56 ± 1.11E-1 |
| dataset 2 | MapSplice | 30-40% | 97.70 ± 1.07E-1 |
| dataset 2 | RUM | 30-40% | 97.66 ± 4.07E-2 |
| dataset 2 | Tophat2 | 30-40% | 96.94 ± 1.40E-1 |
| dataset 2 | GSNAP | 40-50% | 98.79 ± 4.69E-2 |
| dataset 2 | MapSplice | 40-50% | 98.18 ± 5.35E-2 |
| dataset 2 | RUM | 40-50% | 98.21 ± 6.94E-2 |
| dataset 2 | Tophat2 | 40-50% | 97.66 ± 6.78E-2 |
| dataset 2 | GSNAP | 50-60% | 98.93 ± 4.07E-2 |
| dataset 2 | MapSplice | 50-60% | 98.38 ± 2.32E-2 |
| dataset 2 | RUM | 50-60% | 98.36 ± 4.39E-2 |
| dataset 2 | Tophat2 | 50-60% | 97.95 ± 7.21E-2 |
| dataset 2 | GSNAP | 60-70% | 99.01 ± 5.97E-2 |
| dataset 2 | MapSplice | 60-70% | 98.56 ± 5.67E-2 |
| dataset 2 | RUM | 60-70% | 98.61 ± 6.24E-2 |
| dataset 2 | Tophat2 | 60-70% | 98.21 ± 5.95E-2 |
| dataset 2 | GSNAP | 70-80% | 99.14 ± 2.48E-2 |
| dataset 2 | MapSplice | 70-80% | 98.74 ± 5.27E-2 |
| dataset 2 | RUM | 70-80% | 98.85 ± 1.96E-2 |
| dataset 2 | Tophat2 | 70-80% | 98.53 ± 6.27E-2 |
| dataset 2 | GSNAP | 80-90% | 99.16 ± 5.28E-2 |
| dataset 2 | MapSplice | 80-90% | 98.79 ± 2.59E-2 |
| dataset 2 | RUM | 80-90% | 98.93 ± 5.28E-2 |
| dataset 2 | Tophat2 | 80-90% | 98.69 ± 3.25E-2 |
| dataset 2 | GSNAP | 90-100% | 99.18 ± 4.68E-2 |
| dataset 2 | MapSplice | 90-100% | 98.82 ± 2.05E-2 |
| dataset 2 | RUM | 90-100% | 98.97 ± 2.59E-2 |
| dataset 2 | Tophat2 | 90-100% | 98.72 ± 9.72E-2 |

* Note: An RNA-DNA sequence difference is considered properly identified if a minimum of 1 read bearing the sequence difference is present per the aligner. No threshold on the coverage or total number of reads as observed per the aligner is imposed.

* Note: Sites with a simulated coverage value less than 10x per the simulated RNA-Seq dataset are removed from consideration.

# Table S5. Sensitivity of RNA-DNA sequence difference detection in unique versus non-unique regions as determined by BLAT.

| **Dataset** | **Aligner** | **Uniqueness** | **Sensitivity (%)** |
| --- | --- | --- | --- |
| dataset 1 | GSNAP | Non-Unique | 94.98 ± 1.07E-1% |
| dataset 1 | GSNAP | Unique | 99.53 ± 2.84E-3% |
| dataset 1 | MapSplice | Non-Unique | 95.07 ± 1.99E-1% |
| dataset 1 | MapSplice | Unique | 99.21 ± 4.36E-2% |
| dataset 1 | RUM | Non-Unique | 98.82 ± 7.34E-2% |
| dataset 1 | RUM | Unique | 99.05 ± 3.71E-2% |
| dataset 1 | Tophat2 | Non-Unique | 94.90 ± 1.75E-1% |
| dataset 1 | Tophat2 | Unique | 99.13 ± 3.54E-2% |
| dataset 2 | GSNAP | Non-Unique | 94.74 ± 1.44E-1% |
| dataset 2 | GSNAP | Unique | 99.25 ± 2.58E-2% |
| dataset 2 | MapSplice | Non-Unique | 94.16 ± 2.18E-1% |
| dataset 2 | MapSplice | Unique | 98.54 ± 3.45E-2% |
| dataset 2 | RUM | Non-Unique | 96.73 ± 9.75E-2% |
| dataset 2 | RUM | Unique | 98.08 ± 3.42E-2% |
| dataset 2 | Tophat2 | Non-Unique | 93.63 ± 2.15E-1% |
| dataset 2 | Tophat2 | Unique | 97.94 ± 2.19E-2% |

*Note: Sites with a simulated coverage value less than 10x and simulated RNA-DNA sequence difference level less than 10% per the simulated RNA-Seq dataset are not considered in this analysis.

# Table S6. Sensitivity of RDD detection within RepeatMasker regions.

| **Dataset** | **Aligner** | **Repeat Masker** | **Sensitivity (%)** |
| --- | --- | --- | --- |
| dataset 1 | GSNAP | In Repeat Masker Region | 98.48 ± 1.11E-1 |
| dataset 1 | GSNAP | Not In Repeat Masker Region | 99.16 ± 2.04E-2 |
| dataset 1 | MapSplice | In Repeat Masker Region | 97.91 ± 1.32E-1 |
| dataset 1 | MapSplice | Not In Repeat Masker Region | 98.90 ± 3.02E-2 |
| dataset 1 | RUM | In Repeat Masker Region | 97.59 ± 1.60E-1 |
| dataset 1 | RUM | Not In Repeat Masker Region | 99.14 ± 2.04E-2 |
| dataset 1 | Tophat2 | In Repeat Masker Region | 97.55 ± 1.29E-1 |
| dataset 1 | Tophat2 | Not In Repeat Masker Region | 98.83 ± 9.89E-3 |
| dataset 2 | GSNAP | In Repeat Masker Region | 98.01 ± 1.15E-1 |
| dataset 2 | GSNAP | Not In Repeat Masker Region | 98.91 ± 2.18E-2 |
| dataset 2 | MapSplice | In Repeat Masker Region | 97.15 ± 1.53E-1 |
| dataset 2 | MapSplice | Not In Repeat Masker Region | 98.27 ± 5.74E-2 |
| dataset 2 | RUM | Not In Repeat Masker Region | 96.70 ± 1.80E-1 |
| dataset 2 | RUM | Not In Repeat Masker Region | 98.34 ± 8.01E-2 |
| dataset 2 | Tophat2 | Not In Repeat Masker Region | 96.48 ± 2.74E-1 |
| dataset 2 | Tophat2 | Not In Repeat Masker Region | 97.71 ± 3.81E-2 |

*Note: Sites with a simulated coverage value less than 10x and simulated RNA-DNA sequence difference level less than 10% per the simulated RNA-Seq dataset are not considered in this analysis.

# Table S7. Sensitivity of RDD detection versus proximity to nearby RDDs.

| **Dataset** | **Aligner** | **Distance to Nearest RDD** | **Sensitivity (%)** |
| --- | --- | --- | --- |
| dataset 1 | GSNAP | RDD not within 10bp of another RDD | 99.29 ± 1.22E-2 |
| dataset 1 | GSNAP | RDD within 10bp of another RDD | 97.79 ± 4.35E-2 |
| dataset 1 | MapSplice | RDD not within 10bp of another RDD | 99.15 ± 5.37E-3 |
| dataset 1 | MapSplice | RDD within 10bp of another RDD | 96.56 ± 1.64E-1 |
| dataset 1 | RUM | RDD not within 10bp of another RDD | 99.35 ± 1.50E-2 |
| dataset 1 | RUM | RDD within 10bp of another RDD | 96.75 ± 1.71E-1 |
| dataset 1 | Tophat2 | RDD not within 10bp of another RDD | 99.06 ± 7.09E-3 |
| dataset 1 | Tophat2 | RDD within 10bp of another RDD | 96.51 ± 1.09E-1 |
| dataset 2 | GSNAP | RDD not within 10bp of another RDD | 99.06 ± 2.35E-2 |
| dataset 2 | GSNAP | RDD within 10bp of another RDD | 95.66 ± 2.21E-1 |
| dataset 2 | MapSplice | RDD not within 10bp of another RDD | 98.61 ± 1.05E-2 |
| dataset 2 | MapSplice | RDD within 10bp of another RDD | 93.13 ± 5.91E-2 |
| dataset 2 | RUM | RDD not within 10bp of another RDD | 98.62 ± 6.81E-3 |
| dataset 2 | RUM | RDD within 10bp of another RDD | 92.51 ± 1.17E-1 |
| dataset 2 | Tophat2 | RDD not within 10bp of another RDD | 98.12 ± 2.48E-2 |
| dataset 2 | Tophat2 | RDD within 10bp of another RDD | 91.75 ± 2.49E-1 |

*Note: Sites with a simulated coverage less than 10x and a simulated RNA-DNA sequence difference level less than 10% in the simulated dataset are not considered in this analysis.

# Table S8. Correlation between observed and simulated levels of RDDs.

| **Dataset** | **Aligner** | **Correlation (%)** |
| --- | --- | --- |
| dataset 1 | GSNAP | 98.02 ± 2.86E-2 |
| dataset 1 | MapSplice | 98.02 ± 2.88E-2 |
| dataset 1 | RUM | 98.02 ± 2.85E-2 |
| dataset 1 | Tophat2 | 98.03 ± 2.88E-2 |
| dataset 2 | GSNAP | 98.08 ± 2.69E-2 |
| dataset 2 | MapSplice | 98.08 ± 2.68E-2 |
| dataset 2 | RUM | 98.08 ± 2.69E-2 |
| dataset 2 | Tophat2 | 98.09 ± 2.54E-2 |

*Note: Sites with simulated coverage less than 10x and a simulated RNA-DNA sequence difference level less than 10% in the simulated dataset are not considered. Furthermore, sites must be identified as having at least 1 read bearing the RNA-DNA sequence difference in the aligned dataset per GSNAP, MapSplice, RUM, or Tophat2 to be included in this analysis.

# Table S9. Percent of sites with levels where the observed and simulated levels deviate by more than 30% versus the uniqueness of the underlying site as determined by BLAT.

| **Dataset** | **Aligner** | **Levels differ by more than 30%** | **Percent of sites that are not unique as determined by BLAT(%)** |
| --- | --- | --- | --- |
| dataset 1 | GSNAP | TRUE | 27.03 ± 5.57E-1 |
| dataset 1 | GSNAP | FALSE | 8.09 ± 2.57E-2 |
| dataset 1 | MapSplice | TRUE | 22.36 ± 3.56E-1 |
| dataset 1 | MapSplice | FALSE | 8.11 ± 3.43E-2 |
| dataset 1 | RUM | TRUE | 30.98 ± 5.20E-1 |
| dataset 1 | RUM | FALSE | 7.75 ± 1.44E-2 |
| dataset 1 | Tophat2 | TRUE | 31.55 ± 4.40E-1 |
| dataset 1 | Tophat2 | FALSE | 7.78 ± 1.72E-2 |
| dataset 2 | GSNAP | TRUE | 29.20 ± 5.69E-1 |
| dataset 2 | GSNAP | FALSE | 11.38 ± 4.92E-2 |
| dataset 2 | MapSplice | TRUE | 26.02 ± 3.34E-1 |
| dataset 2 | MapSplice | FALSE | 11.38 ± 6.06E-2 |
| dataset 2 | RUM | TRUE | 33.83 ± 4.19E-1 |
| dataset 2 | RUM | FALSE | 10.65 ± 6.50E-2 |
| dataset 2 | Tophat2 | TRUE | 29.17 ± 4.11E-1 |
| dataset 2 | Tophat2 | FALSE | 11.02 ± 5.53E-2 |

# Table S10. Receiver operating characteristic analysis of RNA-DNA sequence difference detection.

| **Dataset** | **Aligner** | **Min. Cov.*** | **Min. Level**‡ | **Min. RDD Count**∆ | **True Negatives** | **True Positives** | **False Negatives** | **False Positives** | **Accuracy (%)** | **Sensitivity or True Positive Rate (%)** | **1 – Specificity or False Positive Rate (%)** | **False Discovery Rate (%)** | **Negative Predictive Value (%)** |
| --- | --- | --- | --- | --- | --- | --- | --- | --- | --- | --- | --- | --- | --- |
| dataset 1 | GSNAP | 0 | 0 | 1 | 5.91E7 ± 2.76E4 | 5.67E5 ± 7.05E2 | 9.46E3 ± 1.55E2 | 5.06E4 ± 1.91E3 | 99.90 ± 3.42E-3 | 98.36 ± 2.78E-2 | 0.09 ± 3.19E-3 | 8.20 ± 2.89E-1 | 99.98 ± 2.58E-4 |
| dataset 1 | GSNAP | 10 | 10 | 1 | 4.71E7 ± 1.91E4 | 4.14E5 ± 2.81E3 | 7.40E3 ± 1.06E2 | 8.22E3 ± 2.58E2 | 99.97 ± 6.97E-4 | 98.24 ± 1.66E-2 | 0.02 ± 5.41E-4 | 1.95 ± 6.00E-2 | 99.98 ± 2.20E-4 |
| dataset 1 | GSNAP | 10 | 10 | 2 | 4.71E7 ± 1.91E4 | 4.13E5 ± 2.77E3 | 7.58E3 ± 1.31E2 | 8.11E3 ± 2.47E2 | 99.97 ± 6.93E-4 | 98.20 ± 2.10E-2 | 0.02 ± 5.19E-4 | 1.92 ± 5.86E-2 | 99.98 ± 2.72E-4 |
| dataset 1 | GSNAP | 10 | 20 | 2 | 4.08E7 ± 4.96E3 | 3.56E5 ± 2.52E3 | 5.88E3 ± 1.19E2 | 5.61E3 ± 1.81E2 | 99.97 ± 7.30E-4 | 98.38 ± 3.13E-2 | 0.01 ± 4.44E-4 | 1.55 ± 4.67E-2 | 99.99 ± 2.93E-4 |
| dataset 1 | GSNAP | 20 | 10 | 2 | 4.71E7 ± 1.92E4 | 3.64E5 ± 2.26E3 | 1.12E4 ± 1.87E2 | 5.98E3 ± 2.11E2 | 99.96 ± 4.82E-4 | 97.02 ± 3.07E-2 | 0.01 ± 4.45E-4 | 1.62 ± 6.14E-2 | 99.98 ± 3.91E-4 |
| dataset 1 | GSNAP | 20 | 20 | 4 | 4.08E7 ± 5.00E3 | 3.13E5 ± 2.02E3 | 9.15E3 ± 1.73E2 | 3.89E3 ± 1.42E2 | 99.97 ± 5.13E-4 | 97.16 ± 3.45E-2 | 0.01 ± 3.50E-4 | 1.23 ± 4.69E-2 | 99.98 ± 4.23E-4 |
| dataset 1 | MapSplice | 0 | 0 | 1 | 5.91E7 ± 1.79E4 | 5.61E5 ± 1.24E3 | 1.09E4 ± 1.99E2 | 1.98E5 ± 5.67E2 | 99.65 ± 1.28E-3 | 98.09 ± 3.79E-2 | 0.33 ± 1.04E-3 | 26.10 ± 8.11E-2 | 99.98 ± 3.37E-4 |
| dataset 1 | MapSplice | 10 | 10 | 1 | 4.71E7 ± 1.27E4 | 4.10E5 ± 3.08E3 | 6.71E3 ± 8.78E1 | 2.73E4 ± 2.10E2 | 99.93 ± 5.06E-4 | 98.39 ± 1.82E-2 | 0.06 ± 4.40E-4 | 6.24 ± 8.26E-2 | 99.99 ± 1.83E-4 |
| dataset 1 | MapSplice | 10 | 10 | 2 | 4.71E7 ± 1.27E4 | 4.10E5 ± 3.05E3 | 6.90E3 ± 1.09E2 | 2.72E4 ± 1.97E2 | 99.93 ± 4.87E-4 | 98.34 ± 2.17E-2 | 0.06 ± 4.13E-4 | 6.22 ± 8.01E-2 | 99.99 ± 2.28E-4 |
| dataset 1 | MapSplice | 10 | 20 | 2 | 4.07E7 ± 6.38E3 | 3.52E5 ± 2.65E3 | 5.21E3 ± 9.95E1 | 1.47E4 ± 2.01E2 | 99.95 ± 4.87E-4 | 98.54 ± 2.47E-2 | 0.04 ± 4.92E-4 | 4.02 ± 8.09E-2 | 99.99 ± 2.46E-4 |
| dataset 1 | MapSplice | 20 | 10 | 2 | 4.71E7 ± 1.27E4 | 3.62E5 ± 2.59E3 | 1.00E4 ± 8.96E1 | 2.43E4 ± 2.30E2 | 99.93 ± 3.70E-4 | 97.31 ± 2.84E-2 | 0.05 ± 4.84E-4 | 6.29 ± 9.14E-2 | 99.98 ± 1.95E-4 |
| dataset 1 | MapSplice | 20 | 20 | 4 | 4.07E7 ± 6.40E3 | 3.10E5 ± 2.27E3 | 7.99E3 ± 7.60E1 | 1.25E4 ± 2.45E2 | 99.95 ± 4.51E-4 | 97.49 ± 1.70E-2 | 0.03 ± 6.00E-4 | 3.87 ± 9.92E-2 | 99.98 ± 1.85E-4 |
| dataset 1 | RUM | 0 | 0 | 1 | 6.01E7 ± 4.47E4 | 5.61E5 ± 1.28E3 | 1.29E4 ± 3.11E2 | 6.56E4 ± 1.42E3 | 99.87 ± 1.75E-3 | 97.76 ± 5.76E-2 | 0.11 ± 2.30E-3 | 10.46 ± 1.83E-1 | 99.98 ± 5.28E-4 |
| dataset 1 | RUM | 10 | 10 | 1 | 4.80E7 ± 1.66E4 | 4.08E5 ± 2.85E3 | 9.45E3 ± 1.74E2 | 2.36E4 ± 3.16E2 | 99.93 ± 4.30E-4 | 97.73 ± 2.52E-2 | 0.05 ± 6.72E-4 | 5.48 ± 1.01E-1 | 99.98 ± 3.60E-4 |
| dataset 1 | RUM | 10 | 10 | 2 | 4.80E7 ± 1.66E4 | 4.07E5 ± 2.85E3 | 9.65E3 ± 1.81E2 | 2.35E4 ± 3.11E2 | 99.93 ± 4.29E-4 | 97.68 ± 2.66E-2 | 0.05 ± 6.61E-4 | 5.45 ± 9.96E-2 | 99.98 ± 3.75E-4 |
| dataset 1 | RUM | 10 | 20 | 2 | 4.15E7 ± 8.02E3 | 3.48E5 ± 2.49E3 | 7.40E3 ± 1.59E2 | 1.83E4 ± 3.47E2 | 99.94 ± 6.64E-4 | 97.92 ± 2.94E-2 | 0.04 ± 8.25E-4 | 5.00 ± 1.09E-1 | 99.98 ± 3.85E-4 |
| dataset 1 | RUM | 20 | 10 | 2 | 4.80E7 ± 1.65E4 | 3.58E5 ± 2.37E3 | 1.41E4 ± 1.95E2 | 1.69E4 ± 2.50E2 | 99.94 ± 4.77E-4 | 96.21 ± 3.37E-2 | 0.04 ± 5.29E-4 | 4.51 ± 9.04E-2 | 99.97 ± 4.09E-4 |
| dataset 1 | RUM | 20 | 20 | 4 | 4.15E7 ± 8.13E3 | 3.05E5 ± 2.05E3 | 1.13E4 ± 2.19E2 | 1.25E4 ± 2.41E2 | 99.94 ± 5.48E-4 | 96.41 ± 4.60E-2 | 0.03 ± 5.74E-4 | 3.95 ± 9.40E-2 | 99.97 ± 5.28E-4 |
| dataset 1 | Tophat2 | 0 | 0 | 1 | 5.27E7 ± 2.20E4 | 4.56E5 ± 2.11E3 | 5.92E4 ± 1.68E2 | 1.50E4 ± 2.50E2 | 99.86 ± 4.94E-4 | 88.50 ± 6.09E-2 | 0.03 ± 4.70E-4 | 3.18 ± 3.72E-2 | 99.89 ± 3.64E-4 |
| dataset 1 | Tophat2 | 10 | 10 | 1 | 3.07E7 ± 6.17E3 | 2.51E5 ± 7.79E2 | 9.81E3 ± 9.57E1 | 3.32E3 ± 1.07E2 | 99.96 ± 4.09E-5 | 96.23 ± 4.05E-2 | 0.01 ± 3.51E-4 | 1.31 ± 4.06E-2 | 99.97 ± 3.11E-4 |
| dataset 1 | Tophat2 | 10 | 10 | 2 | 3.07E7 ± 6.17E3 | 2.50E5 ± 7.50E2 | 1.08E4 ± 7.40E1 | 3.17E3 ± 1.18E2 | 99.95 ± 1.78E-4 | 95.86 ± 2.98E-2 | 0.01 ± 3.84E-4 | 1.26 ± 4.50E-2 | 99.96 ± 2.43E-4 |
| dataset 1 | Tophat2 | 10 | 20 | 2 | 2.02E7 ± 1.77E4 | 1.63E5 ± 5.97E2 | 4.83E3 ± 1.28E2 | 2.02E3 ± 5.63E1 | 99.97 ± 4.50E-4 | 97.12 ± 6.48E-2 | 0.01 ± 2.72E-4 | 1.23 ± 3.59E-2 | 99.98 ± 6.38E-4 |
| dataset 1 | Tophat2 | 20 | 10 | 2 | 3.07E7 ± 6.16E3 | 2.20E5 ± 3.92E2 | 1.16E4 ± 2.07E2 | 1.95E3 ± 7.42E1 | 99.96 ± 7.57E-4 | 95.01 ± 7.64E-2 | 0.01 ± 2.42E-4 | 0.88 ± 3.27E-2 | 99.96 ± 6.81E-4 |
| dataset 1 | Tophat2 | 20 | 20 | 4 | 2.02E7 ± 1.78E4 | 1.43E5 ± 3.90E2 | 6.11E3 ± 1.45E2 | 1.20E3 ± 3.56E1 | 99.96 ± 6.68E-4 | 95.90 ± 8.61E-2 | 0.01 ± 1.73E-4 | 0.83 ± 2.64E-2 | 99.97 ± 7.01E-4 |
| dataset 2 | GSNAP | 0 | 0 | 1 | 5.44E8 ± 1.27E5 | 5.60E5 ± 8.28E2 | 1.33E4 ± 1.86E2 | 3.40E7 ± 3.34E3 | 94.12 ± 7.50E-4 | 97.69 ± 3.44E-2 | 5.89 ± 7.49E-4 | 98.38 ± 2.27E-3 | 100.00 ± 3.42E-5 |
| dataset 2 | GSNAP | 10 | 10 | 1 | 1.06E8 ± 3.49E4 | 3.79E5 ± 7.87E2 | 7.28E3 ± 5.03E1 | 5.03E5 ± 7.59E2 | 99.52 ± 8.19E-4 | 98.11 ± 1.48E-2 | 0.47 ± 8.59E-4 | 57.05 ± 6.61E-2 | 99.99 ± 4.57E-5 |
| dataset 2 | GSNAP | 10 | 10 | 2 | 1.07E8 ± 3.46E4 | 3.78E5 ± 8.00E2 | 7.86E3 ± 5.80E1 | 6.64E4 ± 4.72E2 | 99.93 ± 4.32E-4 | 97.96 ± 1.71E-2 | 0.06 ± 4.61E-4 | 14.95 ± 1.04E-1 | 99.99 ± 5.27E-5 |
| dataset 2 | GSNAP | 10 | 20 | 2 | 6.42E7 ± 1.14E4 | 2.46E5 ± 1.07E3 | 4.38E3 ± 4.94E1 | 1.36E4 ± 3.10E2 | 99.97 ± 4.12E-4 | 98.25 ± 2.70E-2 | 0.02 ± 4.86E-4 | 5.26 ± 9.22E-2 | 99.99 ± 7.58E-5 |
| dataset 2 | GSNAP | 20 | 10 | 2 | 1.07E8 ± 3.43E4 | 3.34E5 ± 7.57E2 | 1.04E4 ± 8.01E1 | 1.20E4 ± 2.33E2 | 99.98 ± 1.97E-4 | 96.98 ± 1.60E-2 | 0.01 ± 2.22E-4 | 3.46 ± 7.01E-2 | 99.99 ± 7.41E-5 |
| dataset 2 | GSNAP | 20 | 20 | 4 | 6.42E7 ± 1.14E4 | 2.15E5 ± 8.11E2 | 6.46E3 ± 8.98E1 | 4.43E3 ± 2.84E2 | 99.98 ± 4.87E-4 | 97.09 ± 3.57E-2 | 0.01 ± 4.43E-4 | 2.02 ± 1.19E-1 | 99.99 ± 1.41E-4 |
| dataset 2 | MapSplice | 0 | 0 | 1 | 5.37E8 ± 1.13E5 | 5.53E5 ± 4.75E2 | 1.53E4 ± 1.11E2 | 4.15E7 ± 5.81E3 | 92.83 ± 1.02E-3 | 97.31 ± 1.68E-2 | 7.17 ± 1.03E-3 | 98.69 ± 1.25E-3 | 100.00 ± 2.06E-5 |
| dataset 2 | MapSplice | 10 | 10 | 1 | 1.06E8 ± 3.63E4 | 3.71E5 ± 5.22E2 | 6.90E3 ± 4.25E1 | 7.30E5 ± 3.81E2 | 99.31 ± 2.96E-4 | 98.17 ± 9.10E-3 | 0.68 ± 2.70E-4 | 66.33 ± 2.27E-2 | 99.99 ± 3.86E-5 |
| dataset 2 | MapSplice | 10 | 10 | 2 | 1.07E8 ± 3.68E4 | 3.70E5 ± 5.26E2 | 7.42E3 ± 6.75E1 | 1.50E5 ± 2.40E2 | 99.85 ± 2.10E-4 | 98.03 ± 1.61E-2 | 0.14 ± 2.70E-4 | 28.89 ± 5.28E-2 | 99.99 ± 6.13E-5 |
| dataset 2 | MapSplice | 10 | 20 | 2 | 6.40E7 ± 1.64E4 | 2.40E5 ± 7.49E2 | 4.17E3 ± 5.36E1 | 3.85E4 ± 2.54E2 | 99.93 ± 4.21E-4 | 98.29 ± 2.61E-2 | 0.06 ± 4.00E-4 | 13.85 ± 7.34E-2 | 99.99 ± 8.22E-5 |
| dataset 2 | MapSplice | 20 | 10 | 2 | 1.07E8 ± 3.67E4 | 3.27E5 ± 3.76E2 | 9.78E3 ± 3.82E2 | 4.39E4 ± 2.18E2 | 99.95 ± 3.21E-4 | 97.10 ± 1.09E-1 | 0.04 ± 2.14E-4 | 11.84 ± 6.37E-2 | 99.99 ± 3.59E-4 |
| dataset 2 | MapSplice | 20 | 20 | 4 | 6.40E7 ± 1.63E4 | 2.10E5 ± 4.05E2 | 6.17E3 ± 2.19E2 | 1.64E4 ± 5.04E1 | 99.96 ± 2.74E-4 | 97.15 ± 9.65E-2 | 0.03 ± 7.27E-5 | 7.25 ± 3.07E-2 | 99.99 ± 3.44E-4 |
| dataset 2 | RUM | 0 | 0 | 1 | 5.50E8 ± 1.27E5 | 5.45E5 ± 4.97E2 | 1.97E4 ± 1.31E2 | 3.67E7 ± 1.36E4 | 93.74 ± 1.83E-3 | 96.52 ± 1.93E-2 | 6.26 ± 1.84E-3 | 98.54 ± 1.84E-3 | 100.00 ± 2.44E-5 |
| dataset 2 | RUM | 10 | 10 | 1 | 1.10E8 ± 2.56E4 | 3.61E5 ± 3.37E2 | 1.03E4 ± 1.83E2 | 6.42E5 ± 2.32E3 | 99.41 ± 2.26E-3 | 97.22 ± 5.03E-2 | 0.58 ± 2.11E-3 | 64.05 ± 1.00E-1 | 99.99 ± 1.66E-4 |
| dataset 2 | RUM | 10 | 10 | 2 | 1.10E8 ± 2.52E4 | 3.60E5 ± 3.18E2 | 1.10E4 ± 1.55E2 | 1.53E5 ± 1.63E3 | 99.85 ± 1.61E-3 | 97.03 ± 4.28E-2 | 0.14 ± 1.48E-3 | 29.87 ± 2.39E-1 | 99.99 ± 1.40E-4 |
| dataset 2 | RUM | 10 | 20 | 2 | 6.62E7 ± 1.26E4 | 2.28E5 ± 7.86E2 | 6.19E3 ± 1.64E2 | 5.14E4 ± 7.82E2 | 99.91 ± 1.41E-3 | 97.36 ± 6.64E-2 | 0.08 ± 1.17E-3 | 18.38 ± 2.07E-1 | 99.99 ± 2.47E-4 |
| dataset 2 | RUM | 20 | 10 | 2 | 1.10E8 ± 2.55E4 | 3.16E5 ± 2.60E2 | 1.47E4 ± 1.04E2 | 3.95E4 ± 4.69E2 | 99.95 ± 4.84E-4 | 95.55 ± 3.07E-2 | 0.04 ± 4.28E-4 | 11.12 ± 1.25E-1 | 99.99 ± 9.71E-5 |
| dataset 2 | RUM | 20 | 20 | 4 | 6.62E7 ± 1.29E4 | 1.99E5 ± 5.67E2 | 9.24E3 ± 1.12E2 | 1.88E4 ± 3.59E2 | 99.96 ± 6.51E-4 | 95.56 ± 3.96E-2 | 0.03 ± 5.40E-4 | 8.67 ± 1.34E-1 | 99.99 ± 1.70E-4 |
| dataset 2 | Tophat2 | 0 | 0 | 1 | 2.65E8 ± 7.51E4 | 3.77E5 ± 4.22E2 | 8.48E4 ± 6.28E2 | 1.35E7 ± 3.91E2 | 95.13 ± 1.57E-3 | 81.63 ± 1.06E-1 | 4.84 ± 1.36E-3 | 97.29 ± 2.88E-3 | 99.97 ± 2.45E-4 |
| dataset 2 | Tophat2 | 10 | 10 | 1 | 3.38E7 ± 1.76E4 | 1.16E5 ± 8.60E2 | 5.49E3 ± 1.08E2 | 2.90E5 ± 1.30E3 | 99.14 ± 3.84E-3 | 95.47 ± 5.23E-2 | 0.85 ± 3.57E-3 | 71.44 ± 5.99E-2 | 99.98 ± 3.14E-4 |
| dataset 2 | Tophat2 | 10 | 10 | 2 | 3.40E7 ± 1.81E4 | 1.15E5 ± 8.74E2 | 6.27E3 ± 1.08E2 | 5.65E4 ± 1.71E2 | 99.82 ± 5.50E-4 | 94.83 ± 5.67E-2 | 0.17 ± 4.14E-4 | 32.94 ± 1.32E-1 | 99.98 ± 3.17E-4 |
| dataset 2 | Tophat2 | 10 | 20 | 2 | 1.82E7 ± 7.78E3 | 6.07E4 ± 6.88E2 | 2.10E3 ± 8.19E1 | 1.00E4 ± 1.36E2 | 99.93 ± 3.87E-4 | 96.66 ± 1.04E-1 | 0.06 ± 7.42E-4 | 14.18 ± 2.98E-1 | 99.99 ± 4.50E-4 |
| dataset 2 | Tophat2 | 20 | 10 | 2 | 3.41E7 ± 1.83E4 | 1.01E5 ± 8.04E2 | 6.48E3 ± 9.92E1 | 6.93E3 ± 2.67E1 | 99.96 ± 2.06E-4 | 93.97 ± 5.72E-2 | 0.02 ± 8.90E-5 | 6.43 ± 6.10E-2 | 99.98 ± 2.81E-4 |
| dataset 2 | Tophat2 | 20 | 20 | 4 | 1.82E7 ± 7.80E3 | 5.30E4 ± 5.90E2 | 2.71E3 ± 7.35E1 | 1.41E3 ± 3.14E1 | 99.98 ± 4.98E-4 | 95.14 ± 7.79E-2 | 0.01 ± 1.70E-4 | 2.60 ± 6.07E-2 | 99.99 ± 4.07E-4 |

* Minimum coverage; ‡ Minimum RDD level; ∆ Minimum RDD count

# Table S11. Effect of requiring RDDs to be concordantly identified by multiple aligners on FDR of RDD detection*.

| **Dataset** | **Aligner** | **Concordant Aligners** | **Number of Concordant Aligners** | **False Discovery Rate** | **True Positives** | **False Positives** |
| --- | --- | --- | --- | --- | --- | --- |
| dataset 1 | GSNAP | GSNAP | 1 | 1.23E-1 ± 4.68E-1 (-0%) | 312,759 ± 2,019 (-0%) | 3,883 ± 142 (-0%) |
| dataset 1 | GSNAP | GSNAP,MapSplice | 2 | 8.27E-1 ± 1.89E-1 (-33%) | 301,030 ± 2,037 (-4%) | 2,509 ± 45 (-35%) |
| dataset 1 | GSNAP | GSNAP,RUM | 2 | 9.04E-1 ± 2.24E-1 (-26%) | 298,507 ± 1,960 (-5%) | 2,724 ± 56 (-30%) |
| dataset 1 | GSNAP | GSNAP,Tophat2 | 2 | 5.12E-1 ± 2.05E-1 (-58%) | 140,746 ± 379 (-55%) | 724 ± 4 (-81%) |
| dataset 1 | GSNAP | GSNAP,MapSplice,RUM | 3 | 7.60E-1 ± 2.39E-1 (-38%) | 294,385 ± 1,963 (-6%) | 2,255 ± 58 (-42%) |
| dataset 1 | GSNAP | GSNAP,MapSplice,Tophat2 | 3 | 4.33E-1 ± 1.09E-1 (-65%) | 139,666 ± 410 (-55%) | 608 ± 15 (-84%) |
| dataset 1 | GSNAP | GSNAP,RUM,Tophat2 | 3 | 4.87E-1 ± 4.40E-1 (-60%) | 139,766 ± 374 (-55%) | 684 ± 5 (-82%) |
| dataset 1 | GSNAP | GSNAP,MapSplice,RUM,Tophat2 | 4 | 4.19E-1 ± 8.90E-1 (-66%) | 139,128 ± 391 (-56%) | 585 ± 11 (-85%) |
| dataset 1 | MapSplice | MapSplice | 1 | 3.86E-1 ± 9.92E-1 (-0%) | 309,968 ± 2,265 (-0%) | 12,438 ± 245 (-0%) |
| dataset 1 | MapSplice | GSNAP,MapSplice | 2 | 8.26E-1 ± 1.91E-1 (-79%) | 301,028 ± 2,036 (-3%) | 2,508 ± 45 (-80%) |
| dataset 1 | MapSplice | MapSplice,RUM | 2 | 1.54E-1 ± 5.22E-1 (-60%) | 298,031 ± 2,046 (-4%) | 4,649 ± 130 (-63%) |
| dataset 1 | MapSplice | MapSplice,Tophat2 | 2 | 4.81E-1 ± 9.70E-1 (-88%) | 141,164 ± 384 (-54%) | 683 ± 15 (-95%) |
| dataset 1 | MapSplice | GSNAP,MapSplice,RUM | 3 | 7.60E-1 ± 2.41E-1 (-80%) | 294,383 ± 1,964 (-5%) | 2,255 ± 58 (-82%) |
| dataset 1 | MapSplice | GSNAP,MapSplice,Tophat2 | 3 | 4.33E-1 ± 1.09E-1 (-89%) | 139,665 ± 409 (-55%) | 608 ± 15 (-95%) |
| dataset 1 | MapSplice | MapSplice,RUM,Tophat2 | 3 | 4.59E-1 ± 5.28E-1 (-88%) | 139,854 ± 384 (-55%) | 645 ± 9 (-95%) |
| dataset 1 | MapSplice | GSNAP,MapSplice,RUM,Tophat2 | 4 | 4.19E-1 ± 8.91E-1 (-89%) | 139,128 ± 391 (-55%) | 585 ± 11 (-95%) |
| dataset 1 | RUM | RUM | 1 | 3.95E-1 ± 9.39E-1 (-0%) | 304,532 ± 2,052 (-0%) | 12,508 ± 241 (-0%) |
| dataset 1 | RUM | GSNAP,RUM | 2 | 9.04E-1 ± 2.26E-1 (-77%) | 298,502 ± 1,960 (-2%) | 2,723 ± 57 (-78%) |
| dataset 1 | RUM | MapSplice,RUM | 2 | 1.54E-1 ± 5.24E-1 (-61%) | 298,028 ± 2,045 (-2%) | 4,652 ± 131 (-63%) |
| dataset 1 | RUM | RUM,Tophat2 | 2 | 6.49E-1 ± 1.68E-1 (-84%) | 140,796 ± 361 (-54%) | 920 ± 24 (-93%) |
| dataset 1 | RUM | GSNAP,MapSplice,RUM | 3 | 7.60E-1 ± 2.41E-1 (-81%) | 294,380 ± 1,963 (-3%) | 2,254 ± 58 (-82%) |
| dataset 1 | RUM | GSNAP,RUM,Tophat2 | 3 | 4.87E-1 ± 4.40E-1 (-88%) | 139,765 ± 373 (-54%) | 684 ± 5 (-95%) |
| dataset 1 | RUM | MapSplice,RUM,Tophat2 | 3 | 4.59E-1 ± 5.28E-1 (-88%) | 139,853 ± 384 (-54%) | 645 ± 9 (-95%) |
| dataset 1 | RUM | GSNAP,MapSplice,RUM,Tophat2 | 4 | 4.19E-1 ± 8.90E-1 (-89%) | 139,127 ± 391 (-54%) | 585 ± 11 (-95%) |
| dataset 1 | Tophat2 | Tophat2 | 1 | 8.33E-1 ± 2.63E-1 (-0%) | 143,022 ± 387 (-0%) | 1,201 ± 35 (-0%) |
| dataset 1 | Tophat2 | GSNAP,Tophat2 | 2 | 5.12E-1 ± 2.05E-1 (-39%) | 140,745 ± 379 (-2%) | 724 ± 4 (-40%) |
| dataset 1 | Tophat2 | MapSplice,Tophat2 | 2 | 4.81E-1 ± 9.70E-1 (-42%) | 141,165 ± 384 (-1%) | 683 ± 15 (-43%) |
| dataset 1 | Tophat2 | RUM,Tophat2 | 2 | 6.49E-1 ± 1.65E-1 (-22%) | 140,797 ± 361 (-2%) | 920 ± 23 (-23%) |
| dataset 1 | Tophat2 | GSNAP,MapSplice,Tophat2 | 3 | 4.33E-1 ± 1.09E-1 (-48%) | 139,665 ± 409 (-2%) | 608 ± 15 (-49%) |
| dataset 1 | Tophat2 | GSNAP,RUM,Tophat2 | 3 | 4.87E-1 ± 4.40E-1 (-42%) | 139,766 ± 373 (-2%) | 684 ± 5 (-43%) |
| dataset 1 | Tophat2 | MapSplice,RUM,Tophat2 | 3 | 4.59E-1 ± 5.27E-1 (-45%) | 139,855 ± 384 (-2%) | 645 ± 9 (-46%) |
| dataset 1 | Tophat2 | GSNAP,MapSplice,RUM,Tophat2 | 4 | 4.19E-1 ± 8.90E-1 (-50%) | 139,128 ± 391 (-3%) | 585 ± 11 (-51%) |
| dataset 2 | GSNAP | GSNAP | 1 | 2.02E-1 ± 1.19E-1 (-0%) | 215,325 ± 813 (-0%) | 4,430 ± 282 (-0%) |
| dataset 2 | GSNAP | GSNAP,MapSplice | 2 | 1.40E-1 ± 6.24E-1 (-30%) | 201,403 ± 540 (-6%) | 2,863 ± 137 (-35%) |
| dataset 2 | GSNAP | GSNAP,RUM | 2 | 1.35E-1 ± 6.12E-1 (-33%) | 193,509 ± 573 (-10%) | 2,654 ± 130 (-40%) |
| dataset 2 | GSNAP | GSNAP,Tophat2 | 2 | 9.65E-1 ± 6.59E-1 (-52%) | 52,159 ± 577 (-76%) | 508 ± 36 (-89%) |
| dataset 2 | GSNAP | GSNAP,MapSplice,RUM | 3 | 1.19E-1 ± 5.60E-1 (-41%) | 190,160 ± 529 (-12%) | 2,290 ± 115 (-48%) |
| dataset 2 | GSNAP | GSNAP,MapSplice,Tophat2 | 3 | 7.94E-1 ± 5.47E-1 (-61%) | 51,715 ± 546 (-76%) | 414 ± 30 (-91%) |
| dataset 2 | GSNAP | GSNAP,RUM,Tophat2 | 3 | 8.47E-1 ± 6.97E-1 (-58%) | 51,523 ± 576 (-76%) | 440 ± 37 (-90%) |
| dataset 2 | GSNAP | GSNAP,MapSplice,RUM,Tophat2 | 4 | 7.60E-1 ± 5.62E-1 (-62%) | 51,314 ± 557 (-76%) | 393 ± 30 (-91%) |
| dataset 2 | MapSplice | MapSplice | 1 | 7.22E-1 ± 2.86E-1 (-0%) | 210,138 ± 413 (-0%) | 16,361 ± 46 (-0%) |
| dataset 2 | MapSplice | GSNAP,MapSplice | 2 | 1.40E-1 ± 6.21E-1 (-81%) | 201,397 ± 540 (-4%) | 2,861 ± 136 (-83%) |
| dataset 2 | MapSplice | MapSplice,RUM | 2 | 2.21E-1 ± 2.59E-1 (-69%) | 192,979 ± 486 (-8%) | 4,361 ± 63 (-73%) |
| dataset 2 | MapSplice | MapSplice,Tophat2 | 2 | 1.07E-1 ± 4.07E-1 (-85%) | 52,189 ± 546 (-75%) | 565 ± 19 (-97%) |
| dataset 2 | MapSplice | GSNAP,MapSplice,RUM | 3 | 1.19E-1 ± 5.57E-1 (-84%) | 190,153 ± 531 (-10%) | 2,289 ± 115 (-86%) |
| dataset 2 | MapSplice | GSNAP,MapSplice,Tophat2 | 3 | 7.93E-1 ± 5.51E-1 (-89%) | 51,714 ± 547 (-75%) | 413 ± 30 (-97%) |
| dataset 2 | MapSplice | MapSplice,RUM,Tophat2 | 3 | 8.60E-1 ± 4.57E-1 (-88%) | 51,528 ± 557 (-75%) | 447 ± 26 (-97%) |
| dataset 2 | MapSplice | GSNAP,MapSplice,RUM,Tophat2 | 4 | 7.60E-1 ± 5.62E-1 (-89%) | 51,312 ± 558 (-76%) | 393 ± 30 (-98%) |
| dataset 2 | RUM | RUM | 1 | 8.65E-1 ± 1.33E-1 (-0%) | 198,603 ± 567 (-0%) | 18,802 ± 355 (-0%) |
| dataset 2 | RUM | GSNAP,RUM | 2 | 1.35E-1 ± 6.14E-1 (-84%) | 193,504 ± 572 (-3%) | 2,653 ± 130 (-86%) |
| dataset 2 | RUM | MapSplice,RUM | 2 | 2.21E-1 ± 2.80E-1 (-74%) | 192,980 ± 483 (-3%) | 4,364 ± 67 (-77%) |
| dataset 2 | RUM | RUM,Tophat2 | 2 | 1.17E-1 ± 5.61E-1 (-86%) | 51,837 ± 579 (-74%) | 616 ± 32 (-97%) |
| dataset 2 | RUM | GSNAP,MapSplice,RUM | 3 | 1.19E-1 ± 5.60E-1 (-86%) | 190,154 ± 528 (-4%) | 2,289 ± 115 (-88%) |
| dataset 2 | RUM | GSNAP,RUM,Tophat2 | 3 | 8.47E-1 ± 6.97E-1 (-90%) | 51,522 ± 577 (-74%) | 440 ± 37 (-98%) |
| dataset 2 | RUM | MapSplice,RUM,Tophat2 | 3 | 8.59E-1 ± 4.54E-1 (-90%) | 51,528 ± 557 (-74%) | 447 ± 26 (-98%) |
| dataset 2 | RUM | GSNAP,MapSplice,RUM,Tophat2 | 4 | 7.60E-1 ± 5.62E-1 (-91%) | 51,313 ± 558 (-74%) | 393 ± 30 (-98%) |
| dataset 2 | Tophat2 | Tophat2 | 1 | 2.57E-1 ± 6.38E-1 (-0%) | 52,951 ± 592 (-0%) | 1,396 ± 35 (-0%) |
| dataset 2 | Tophat2 | GSNAP,Tophat2 | 2 | 9.63E-1 ± 6.69E-1 (-63%) | 52,152 ± 575 (-2%) | 507 ± 36 (-64%) |
| dataset 2 | Tophat2 | MapSplice,Tophat2 | 2 | 1.07E-1 ± 3.96E-1 (-58%) | 52,183 ± 544 (-1%) | 563 ± 18 (-60%) |
| dataset 2 | Tophat2 | RUM,Tophat2 | 2 | 1.18E-1 ± 5.54E-1 (-54%) | 51,831 ± 577 (-2%) | 616 ± 31 (-56%) |
| dataset 2 | Tophat2 | GSNAP,MapSplice,Tophat2 | 3 | 7.94E-1 ± 5.58E-1 (-69%) | 51,708 ± 544 (-2%) | 414 ± 30 (-70%) |
| dataset 2 | Tophat2 | GSNAP,RUM,Tophat2 | 3 | 8.47E-1 ± 6.97E-1 (-67%) | 51,516 ± 575 (-3%) | 440 ± 37 (-68%) |
| dataset 2 | Tophat2 | MapSplice,RUM,Tophat2 | 3 | 8.60E-1 ± 4.57E-1 (-67%) | 51,523 ± 555 (-3%) | 447 ± 26 (-68%) |
| dataset 2 | Tophat2 | GSNAP,MapSplice,RUM,Tophat2 | 4 | 7.60E-1 ± 5.62E-1 (-70%) | 51,307 ± 556 (-3%) | 393 ± 30 (-72%) |

*Note: A ‘minimum coverage of 20x, minimum RDD level of 20%, and minimum of 4 RDD bases’ threshold was required in this analysis.

# Table S12. Percentage of true versus false positives removed by BLAT filter.

| **Dataset** | **Aligner** | **Minimum Coverage** | **Minimum Level** | **Minimum RDD Count** | **Region** | **Percent of True Positives Removed (%)** | **Percent of False Positives Removed (%)** |
| --- | --- | --- | --- | --- | --- | --- | --- |
| dataset 1 | GSNAP | 10 | 10 | 2 | Rmsk | 18.59 + 4.13E-1 | 24.77 ± 4.61 |
| dataset 1 | GSNAP | 10 | 10 | 2 | Not in Rmsk | 0.25 ± 1.66E-2 | 14.56 ± 1.30 |
| dataset 1 | GSNAP | 10 | 10 | 2 | Total | 1.55 + 5.85E-2 | 15.33 ± 1.56 |
| dataset 1 | MapSplice | 10 | 10 | 2 | Rmsk | 18.71 + 2.94E-1 | 24.65 ± 5.36 |
| dataset 1 | MapSplice | 10 | 10 | 2 | Not in Rmsk | 0.25 + 1.72E-2 | 27.04 ± 0.33 |
| dataset 1 | MapSplice | 10 | 10 | 2 | Total | 1.56 + 5.00E-2 | 26.83 ± 0.73 |
| dataset 1 | RUM | 10 | 10 | 2 | Rmsk | 18.37 + 1.74E-1 | 15.49 ± 2.66 |
| dataset 1 | RUM | 10 | 10 | 2 | Not in Rmsk | 0.26 + 1.65E-2 | 4.80 ± 0.34 |
| dataset 1 | RUM | 10 | 10 | 2 | Total | 1.52 + 4.19E-2 | 5.52 ± 0.28 |
| dataset 1 | Tophat2 | 10 | 10 | 2 | Rmsk | 17.75 + 5.44E-1 | 27.00 ± 4.25 |
| dataset 1 | Tophat2 | 10 | 10 | 2 | Not in Rmsk | 0.23 + 1.02E-2 | 17.16 ± 1.34 |
| dataset 1 | Tophat2 | 10 | 10 | 2 | Total | 1.27 + 9.01E-3 | 17.89 ± 1.54 |
| dataset 1 | GSNAP | 10 | 20 | 2 | Rmsk | 18.53 + 4.64E-1 | 25.79 ± 6.71 |
| dataset 1 | GSNAP | 10 | 20 | 2 | Not in Rmsk | 0.25 + 1.51E-2 | 32.94 ± 0.45 |
| dataset 1 | GSNAP | 10 | 20 | 2 | Total | 1.54 + 6.34E-2 | 32.36 ± 0.96 |
| dataset 1 | MapSplice | 10 | 20 | 2 | Rmsk | 18.64 + 2.65E-1 | 14.58 ± 2.17 |
| dataset 1 | MapSplice | 10 | 20 | 2 | Not in Rmsk | 0.25 + 1.75E-2 | 3.99 ± 0.33 |
| dataset 1 | MapSplice | 10 | 20 | 2 | Total | 1.55 + 5.00E-2 | 4.75 ± 0.15 |
| dataset 1 | RUM | 10 | 20 | 2 | Rmsk | 18.25 + 2.02E-1 | 21.18 ± 4.18 |
| dataset 1 | RUM | 10 | 20 | 2 | Not in Rmsk | 0.25 + 1.42E-2 | 11.42 ± 1.65 |
| dataset 1 | RUM | 10 | 20 | 2 | Total | 1.50 + 4.27E-2 | 12.09 ± 1.84 |
| dataset 1 | Tophat2 | 10 | 20 | 2 | Rmsk | 17.63 + 4.85E-1 | 22.56 ± 6.05 |
| dataset 1 | Tophat2 | 10 | 20 | 2 | Not in Rmsk | 0.23 + 1.38E-2 | 24.80 ± 0.41 |
| dataset 1 | Tophat2 | 10 | 20 | 2 | Total | 1.26 + 7.85E-3 | 24.60 ± 0.70 |
| dataset 1 | GSNAP | 20 | 10 | 2 | Rmsk | 18.84 + 5.29E-1 | 15.91 ± 3.32 |
| dataset 1 | GSNAP | 20 | 10 | 2 | Not in Rmsk | 0.24 + 1.64E-2 | 4.55 ± 0.52 |
| dataset 1 | GSNAP | 20 | 10 | 2 | Total | 1.47 + 6.57E-2 | 5.31 ± 0.27 |
| dataset 1 | MapSplice | 20 | 10 | 2 | Rmsk | 18.87 + 2.75E-1 | 21.96 ± 4.78 |
| dataset 1 | MapSplice | 20 | 10 | 2 | Not in Rmsk | 0.24 + 1.34E-2 | 13.56 ± 1.72 |
| dataset 1 | MapSplice | 20 | 10 | 2 | Total | 1.47 + 4.14E-2 | 14.11 ± 1.94 |
| dataset 1 | RUM | 20 | 10 | 2 | Rmsk | 18.30 + 2.49E-1 | 23.57 ± 7.54 |
| dataset 1 | RUM | 20 | 10 | 2 | Not in Rmsk | 0.25 + 1.37E-2 | 30.90 ± 0.54 |
| dataset 1 | RUM | 20 | 10 | 2 | Total | 1.41 + 2.95E-2 | 30.31 ± 1.07 |
| dataset 1 | Tophat2 | 20 | 10 | 2 | Rmsk | 16.29 + 5.97E-1 | 14.81 ± 2.89 |
| dataset 1 | Tophat2 | 20 | 10 | 2 | Not in Rmsk | 0.23 + 1.07E-2 | 3.73 ± 0.47 |
| dataset 1 | Tophat2 | 20 | 10 | 2 | Total | 1.08 + 2.24E-3 | 4.52 ± 0.19 |
| dataset 1 | GSNAP | 20 | 20 | 4 | Rmsk | 18.75 + 6.19E-1 | 25.51 ± 0.30 |
| dataset 1 | GSNAP | 20 | 20 | 4 | Not in Rmsk | 0.23 + 1.44E-2 | 5.87 ± 0.18 |
| dataset 1 | GSNAP | 20 | 20 | 4 | Total | 1.46 + 7.30E-2 | 12.40 ± 0.20 |
| dataset 1 | MapSplice | 20 | 20 | 4 | Rmsk | 18.77 + 2.76E-1 | 35.28 ± 0.25 |
| dataset 1 | MapSplice | 20 | 20 | 4 | Not in Rmsk | 0.23 + 1.47E-2 | 14.73 ± 0.09 |
| dataset 1 | MapSplice | 20 | 20 | 4 | Total | 1.46 + 4.43E-2 | 21.84 ± 0.13 |
| dataset 1 | RUM | 20 | 20 | 4 | Rmsk | 18.16 + 2.24E-1 | 23.39 ± 0.70 |
| dataset 1 | RUM | 20 | 20 | 4 | Not in Rmsk | 0.24 + 1.18E-2 | 0.73 ± 0.02 |
| dataset 1 | RUM | 20 | 20 | 4 | Total | 1.39 + 3.15E-2 | 4.04 ± 0.08 |
| dataset 1 | Tophat2 | 20 | 20 | 4 | Rmsk | 16.03 + 4.71E-1 | 27.37 ± 1.06 |
| dataset 1 | Tophat2 | 20 | 20 | 4 | Not in Rmsk | 0.23 + 9.98E-3 | 23.15 ± 0.43 |
| dataset 1 | Tophat2 | 20 | 20 | 4 | Total | 1.06 + 9.81E-3 | 24.77 + 4.61E0 |
| dataset 2 | GSNAP | 10 | 10 | 2 | Rmsk | 24.77 + 4.75E-1 | 14.56 + 1.30E0 |
| dataset 2 | GSNAP | 10 | 10 | 2 | Not in Rmsk | 0.22 + 1.62E-2 | 15.33 + 1.56E0 |
| dataset 2 | GSNAP | 10 | 10 | 2 | Total | 6.43 + 7.00E-2 | 25.15 + 2.38E0 |
| dataset 2 | MapSplice | 10 | 10 | 2 | Rmsk | 24.41 + 3.88E-1 | 15.89 + 3.27E-1 |
| dataset 2 | MapSplice | 10 | 10 | 2 | Not in Rmsk | 0.22 + 1.70E-2 | 16.15 + 3.80E-1 |
| dataset 2 | MapSplice | 10 | 10 | 2 | Total | 6.31 + 5.42E-2 | 24.65 + 5.36E0 |
| dataset 2 | RUM | 10 | 10 | 2 | Rmsk | 23.18 + 3.12E-1 | 27.04 + 3.33E-1 |
| dataset 2 | RUM | 10 | 10 | 2 | Not in Rmsk | 0.23 + 1.94E-2 | 26.83 + 7.30E-1 |
| dataset 2 | RUM | 10 | 10 | 2 | Total | 5.86 + 4.79E-2 | 15.49 + 2.66E0 |
| dataset 2 | Tophat2 | 10 | 10 | 2 | Rmsk | 22.09 + 2.85E-1 | 4.80 + 3.40E-1 |
| dataset 2 | Tophat2 | 10 | 10 | 2 | Not in Rmsk | 0.23 + 2.33E-2 | 5.52 + 2.77E-1 |
| dataset 2 | Tophat2 | 10 | 10 | 2 | Total | 2.91 + 1.92E-2 | 27.00 + 4.25E0 |
| dataset 2 | GSNAP | 10 | 20 | 2 | Rmsk | 24.78 + 3.90E-1 | 17.16 + 1.34E0 |
| dataset 2 | GSNAP | 10 | 20 | 2 | Not in Rmsk | 0.21 + 1.42E-2 | 17.89 + 1.54E0 |
| dataset 2 | GSNAP | 10 | 20 | 2 | Total | 6.44 + 5.33E-2 | 24.74 + 1.99E0 |
| dataset 2 | MapSplice | 10 | 20 | 2 | Rmsk | 24.39 + 3.36E-1 | 15.88 + 3.88E-1 |
| dataset 2 | MapSplice | 10 | 20 | 2 | Not in Rmsk | 0.21 + 1.83E-2 | 16.10 + 4.30E-1 |
| dataset 2 | MapSplice | 10 | 20 | 2 | Total | 6.32 + 4.33E-2 | 25.79 + 6.71E0 |
| dataset 2 | RUM | 10 | 20 | 2 | Rmsk | 23.11 + 2.46E-1 | 32.94 + 4.54E-1 |
| dataset 2 | RUM | 10 | 20 | 2 | Not in Rmsk | 0.22 + 2.07E-2 | 32.36 + 9.62E-1 |
| dataset 2 | RUM | 10 | 20 | 2 | Total | 5.86 + 4.28E-2 | 14.58 + 2.17E0 |
| dataset 2 | Tophat2 | 10 | 20 | 2 | Rmsk | 22.00 + 2.22E-1 | 3.99 + 3.31E-1 |
| dataset 2 | Tophat2 | 10 | 20 | 2 | Not in Rmsk | 0.22 + 2.23E-2 | 4.75 + 1.54E-1 |
| dataset 2 | Tophat2 | 10 | 20 | 2 | Total | 2.89 + 3.23E-2 | 21.18 + 4.18E0 |
| dataset 2 | GSNAP | 20 | 10 | 2 | Not in Rmsk | 24.25 + 6.09E-1 | 11.42 + 1.65E0 |
| dataset 2 | GSNAP | 20 | 10 | 2 | Total | 0.23 + 1.69E-2 | 12.09 + 1.84E0 |
| dataset 2 | GSNAP | 20 | 10 | 2 | Rmsk | 4.84 + 9.03E-2 | 24.87 + 3.14E0 |
| dataset 2 | MapSplice | 20 | 10 | 2 | Not in Rmsk | 23.66 + 5.83E-1 | 20.50 + 3.57E-1 |
| dataset 2 | MapSplice | 20 | 10 | 2 | Total | 0.23 + 1.96E-2 | 20.64 + 4.30E-1 |
| dataset 2 | MapSplice | 20 | 10 | 2 | Rmsk | 4.72 + 9.43E-2 | 22.56 + 6.05E0 |
| dataset 2 | RUM | 20 | 10 | 2 | Not in Rmsk | 22.34 + 4.96E-1 | 24.80 + 4.06E-1 |
| dataset 2 | RUM | 20 | 10 | 2 | Total | 0.24 + 2.28E-2 | 24.60 + 7.00E-1 |
| dataset 2 | RUM | 20 | 10 | 2 | Not in Rmsk | 4.32 + 7.34E-2 | 15.91 + 3.32E0 |
| dataset 2 | Tophat2 | 20 | 10 | 2 | Total | 19.57 + 1.07E0 | 4.55 + 5.23E-1 |
| dataset 2 | Tophat2 | 20 | 10 | 2 | Rmsk | 0.25 + 2.06E-2 | 5.31 + 2.71E-1 |
| dataset 2 | Tophat2 | 20 | 10 | 2 | Not in Rmsk | 1.96 + 9.66E-2 | 21.96 + 4.78E0 |
| dataset 2 | GSNAP | 20 | 20 | 4 | Total | 24.27 + 4.80E-1 | 13.56 + 1.72E0 |
| dataset 2 | GSNAP | 20 | 20 | 4 | Rmsk | 0.22 + 1.48E-2 | 14.11 + 1.94E0 |
| dataset 2 | GSNAP | 20 | 20 | 4 | Not in Rmsk | 4.83 + 6.37E-2 | 23.46 + 3.78E0 |
| dataset 2 | MapSplice | 20 | 20 | 4 | Total | 23.67 + 5.00E-1 | 21.16 + 4.10E-1 |
| dataset 2 | MapSplice | 20 | 20 | 4 | Not in Rmsk | 0.22 + 1.99E-2 | 21.22 + 4.90E-1 |
| dataset 2 | MapSplice | 20 | 20 | 4 | Total | 4.72 + 7.90E-2 | 23.57 + 7.54E0 |
| dataset 2 | RUM | 20 | 20 | 4 | Rmsk | 22.26 + 4.05E-1 | 30.90 + 5.38E-1 |
| dataset 2 | RUM | 20 | 20 | 4 | Not in Rmsk | 0.23 + 2.26E-2 | 30.31 + 1.07E0 |
| dataset 2 | RUM | 20 | 20 | 4 | Total | 4.29 + 5.56E-2 | 14.81 + 2.89E0 |
| dataset 2 | Tophat2 | 20 | 20 | 4 | Rmsk | 19.56 + 1.18E0 | 3.73 + 4.68E-1 |
| dataset 2 | Tophat2 | 20 | 20 | 4 | Not in Rmsk | 0.23 + 1.27E-2 | 4.52 + 1.90E-1 |
| dataset 2 | Tophat2 | 20 | 20 | 4 | Total | 1.94 + 9.82E-2 | 25.51 + 3.04E-1 |

# Table S13. Effect of BLAT filter on false discovery rate of RDD detection.

| **Dataset** | **Aligner** | **Minimum Coverage** | **Minimum Level** | **Minimum RDD Count** | **Region** | **FDR Before BLAT Filter (%)** | **FDR After BLAT Filter (%)** | **Percent Decrease in FDR (%)** |
| --- | --- | --- | --- | --- | --- | --- | --- | --- |
| dataset 1 | GSNAP | 10 | 10 | 2 | Rmsk | 2.02 ± 1.82E-1 | 1.86 ± 1.02E-1 | 7.46 ± 5.45E1 |
| dataset 1 | GSNAP | 10 | 10 | 2 | Not in Rmsk | 1.92 ± 5.28E-1 | 1.65 ± 2.30E-1 | 14.11 ± 1.27E1 |
| dataset 1 | GSNAP | 10 | 10 | 2 | Total | 1.92 ± 5.86E-1 | 1.66 ± 2.40E-1 | 13.76 ± 1.52E1 |
| dataset 1 | MapSplice | 10 | 10 | 2 | Rmsk | 2.61 ± 1.82E-1 | 2.41 ± 1.94E-1 | 7.74 ± 2.70E1 |
| dataset 1 | MapSplice | 10 | 10 | 2 | Not in Rmsk | 6.48 ± 7.01E-1 | 5.52 ± 4.80E-1 | 14.81 ± 2.94E-1 |
| dataset 1 | MapSplice | 10 | 10 | 2 | Total | 6.22 ± 8.01E-1 | 5.35 ± 5.66E-1 | 14.03 ± 3.55E-1 |
| dataset 1 | RUM | 10 | 10 | 2 | Rmsk | 6.06 ± 7.05E-1 | 5.64 ± 9.96E-1 | 7.31 ± 6.14E1 |
| dataset 1 | RUM | 10 | 10 | 2 | Not in Rmsk | 5.41 ± 5.79E-1 | 4.01 ± 6.08E-1 | 25.77 ± 3.31E-1 |
| dataset 1 | RUM | 10 | 10 | 2 | Total | 5.45 ± 9.96E-1 | 4.11 ± 1.13E-1 | 24.65 ± 7.12E-1 |
| dataset 1 | Tophat2 | 10 | 10 | 2 | Rmsk | 1.43 ± 1.72E-1 | 1.48 ± 2.07E-1 | -2.73 ± 3.84E1 |
| dataset 1 | Tophat2 | 10 | 10 | 2 | Not in Rmsk | 1.24 ± 3.79E-1 | 1.19 ± 4.02E-1 | 4.52 ± 3.29E-1 |
| dataset 1 | Tophat2 | 10 | 10 | 2 | Total | 1.26 ± 4.50E-1 | 1.20 ± 4.63E-1 | 4.25 ± 2.87E-1 |
| dataset 1 | GSNAP | 10 | 20 | 2 | Rmsk | 1.68 ± 1.97E-1 | 1.50 ± 1.31E-1 | 10.25 ± 4.92E1 |
| dataset 1 | GSNAP | 10 | 20 | 2 | Not in Rmsk | 1.61 ± 5.18E-1 | 1.34 ± 3.70E-1 | 16.72 ± 1.31E1 |
| dataset 1 | GSNAP | 10 | 20 | 2 | Total | 1.61 ± 6.15E-1 | 1.35 ± 4.21E-1 | 16.38 ± 1.51E1 |
| dataset 1 | MapSplice | 10 | 20 | 2 | Rmsk | 2.28 ± 1.26E-1 | 2.11 ± 1.04E-1 | 7.34 ± 2.31E1 |
| dataset 1 | MapSplice | 10 | 20 | 2 | Not in Rmsk | 6.56 ± 8.40E-1 | 5.59 ± 5.56E-1 | 14.79 ± 3.49E-1 |
| dataset 1 | MapSplice | 10 | 20 | 2 | Total | 6.27 ± 9.16E-1 | 5.40 ± 6.11E-1 | 13.98 ± 4.03E-1 |
| dataset 1 | RUM | 10 | 20 | 2 | Rmsk | 4.97 ± 7.25E-1 | 4.56 ± 1.01E1 | 8.86 ± 7.76E1 |
| dataset 1 | RUM | 10 | 20 | 2 | Not in Rmsk | 4.47 ± 5.90E-1 | 3.05 ± 6.05E-1 | 31.78 ± 4.61E-1 |
| dataset 1 | RUM | 10 | 20 | 2 | Total | 4.50 ± 9.07E-1 | 3.14 ± 1.06E-1 | 30.35 ± 9.52E-1 |
| dataset 1 | Tophat2 | 10 | 20 | 2 | Rmsk | 1.05 ± 1.29E-1 | 1.09 ± 1.31E-1 | -3.67 ± 3.03E1 |
| dataset 1 | Tophat2 | 10 | 20 | 2 | Not in Rmsk | 0.87 ± 2.64E-1 | 0.83 ± 2.65E-1 | 3.74 ± 3.23E-1 |
| dataset 1 | Tophat2 | 10 | 20 | 2 | Total | 0.88 ± 3.24E-1 | 0.85 ± 3.10E-1 | 3.50 ± 1.51E-1 |
| dataset 1 | GSNAP | 20 | 10 | 2 | Rmsk | 1.58 ± 1.22E-1 | 1.53 ± 6.73E-1 | 2.86 ± 4.50E1 |
| dataset 1 | GSNAP | 20 | 10 | 2 | Not in Rmsk | 1.55 ± 4.32E-1 | 1.38 ± 1.52E-1 | 11.06 ± 1.61E1 |
| dataset 1 | GSNAP | 20 | 10 | 2 | Total | 1.55 ± 4.67E-1 | 1.39 ± 1.46E-1 | 10.62 ± 1.80E1 |
| dataset 1 | MapSplice | 20 | 10 | 2 | Rmsk | 1.95 ± 1.08E-1 | 1.81 ± 1.49E-1 | 7.28 ± 3.51E1 |
| dataset 1 | MapSplice | 20 | 10 | 2 | Not in Rmsk | 4.16 ± 7.61E-1 | 3.35 ± 6.00E-1 | 19.63 ± 3.36E-1 |
| dataset 1 | MapSplice | 20 | 10 | 2 | Total | 4.02 ± 8.09E-1 | 3.26 ± 6.59E-1 | 18.82 ± 3.99E-1 |
| dataset 1 | RUM | 20 | 10 | 2 | Rmsk | 5.82 ± 8.40E-1 | 5.56 ± 1.17E1 | 4.97 ± 7.08E1 |
| dataset 1 | RUM | 20 | 10 | 2 | Not in Rmsk | 4.95 ± 5.83E-1 | 3.77 ± 5.93E-1 | 23.68 ± 3.94E-1 |
| dataset 1 | RUM | 20 | 10 | 2 | Total | 5.00 ± 1.09E-1 | 3.87 ± 1.19E-1 | 22.61 ± 6.89E-1 |
| dataset 1 | Tophat2 | 20 | 10 | 2 | Rmsk | 1.52 ± 2.09E-1 | 1.52 ± 2.12E-1 | -0.47 ± 4.57E1 |
| dataset 1 | Tophat2 | 20 | 10 | 2 | Not in Rmsk | 1.21 ± 2.66E-1 | 1.16 ± 3.13E-1 | 4.28 ± 5.12E-1 |
| dataset 1 | Tophat2 | 20 | 10 | 2 | Total | 1.23 ± 3.59E-1 | 1.18 ± 3.76E-1 | 4.22 ± 2.70E-1 |
| dataset 1 | GSNAP | 20 | 20 | 4 | Rmsk | 1.20 ± 9.77E-1 | 1.15 ± 6.29E-1 | 3.93 ± 5.10E1 |
| dataset 1 | GSNAP | 20 | 20 | 4 | Not in Rmsk | 1.23 ± 4.32E-1 | 1.07 ± 2.45E-1 | 13.22 ± 1.70E1 |
| dataset 1 | GSNAP | 20 | 20 | 4 | Total | 1.23 ± 4.68E-1 | 1.07 ± 2.65E-1 | 12.70 ± 1.89E1 |
| dataset 1 | MapSplice | 20 | 20 | 4 | Rmsk | 1.58 ± 6.63E-1 | 1.49 ± 7.08E-1 | 5.70 ± 4.26E1 |
| dataset 1 | MapSplice | 20 | 20 | 4 | Not in Rmsk | 4.02 ± 9.82E-1 | 3.20 ± 7.47E-1 | 20.30 ± 3.84E-1 |
| dataset 1 | MapSplice | 20 | 20 | 4 | Total | 3.86 ± 9.92E-1 | 3.11 ± 7.64E-1 | 19.43 ± 4.56E-1 |
| dataset 1 | RUM | 20 | 20 | 4 | Rmsk | 4.54 ± 8.60E-1 | 4.29 ± 1.19E1 | 6.40 ± 8.84E1 |
| dataset 1 | RUM | 20 | 20 | 4 | Not in Rmsk | 3.90 ± 5.60E-1 | 2.74 ± 4.58E-1 | 29.89 ± 5.24E-1 |
| dataset 1 | RUM | 20 | 20 | 4 | Total | 3.95 ± 9.39E-1 | 2.82 ± 1.06E-1 | 28.49 ± 1.06E1 |
| dataset 1 | Tophat2 | 20 | 20 | 4 | Rmsk | 1.11 ± 1.28E-1 | 1.13 ± 1.29E-1 | -1.44 ± 3.84E1 |
| dataset 1 | Tophat2 | 20 | 20 | 4 | Not in Rmsk | 0.82 ± 2.07E-1 | 0.79 ± 2.17E-1 | 3.48 ± 4.61E-1 |
| dataset 1 | Tophat2 | 20 | 20 | 4 | Total | 0.83 ± 2.63E-1 | 0.80 ± 2.57E-1 | 3.47 ± 2.00E-1 |
| dataset 2 | GSNAP | 10 | 10 | 2 | Rmsk | 18.77 ± 1.69E-1 | 18.62 ± 2.01E-1 | 0.79 ± 1.95E-1 |
| dataset 2 | GSNAP | 10 | 10 | 2 | Not in Rmsk | 13.57 ± 1.33E-1 | 12.90 ± 1.17E-1 | 4.93 ± 1.64E-1 |
| dataset 2 | GSNAP | 10 | 10 | 2 | Total | 14.94 ± 1.04E-1 | 14.13 ± 9.57E-1 | 5.48 ± 1.35E-1 |
| dataset 2 | MapSplice | 10 | 10 | 2 | Rmsk | 29.53 ± 1.65E-1 | 29.14 ± 2.11E-1 | 1.32 ± 2.59E-1 |
| dataset 2 | MapSplice | 10 | 10 | 2 | Not in Rmsk | 28.68 ± 1.15E-1 | 27.18 ± 1.13E-1 | 5.23 ± 6.60E-1 |
| dataset 2 | MapSplice | 10 | 10 | 2 | Total | 28.89 ± 5.26E-1 | 27.58 ± 7.46E-1 | 4.53 ± 1.02E-1 |
| dataset 2 | RUM | 10 | 10 | 2 | Rmsk | 37.54 ± 2.71E-1 | 33.62 ± 2.85E-1 | 10.45 ± 2.75E-1 |
| dataset 2 | RUM | 10 | 10 | 2 | Not in Rmsk | 26.95 ± 2.64E-1 | 23.98 ± 2.58E-1 | 11.05 ± 1.08E-1 |
| dataset 2 | RUM | 10 | 10 | 2 | Total | 29.87 ± 2.39E-1 | 26.13 ± 2.12E-1 | 12.54 ± 6.86E-1 |
| dataset 2 | Tophat2 | 10 | 10 | 2 | Rmsk | 36.97 ± 7.13E-1 | 36.58 ± 1.01E-1 | 1.06 ± 4.05E-1 |
| dataset 2 | Tophat2 | 10 | 10 | 2 | Not in Rmsk | 32.34 ± 1.48E-1 | 32.23 ± 1.46E-1 | 0.34 ± 4.90E-1 |
| dataset 2 | Tophat2 | 10 | 10 | 2 | Total | 32.94 ± 1.33E-1 | 32.68 ± 1.17E-1 | 0.78 ± 4.65E-1 |
| dataset 2 | GSNAP | 10 | 20 | 2 | Rmsk | 3.48 ± 5.32E-1 | 3.36 ± 1.29E-1 | 3.33 ± 1.38E1 |
| dataset 2 | GSNAP | 10 | 20 | 2 | Not in Rmsk | 3.45 ± 8.49E-1 | 2.68 ± 5.23E-1 | 22.37 ± 4.07E-1 |
| dataset 2 | GSNAP | 10 | 20 | 2 | Total | 3.46 ± 7.06E-1 | 2.82 ± 3.98E-1 | 18.48 ± 5.07E-1 |
| dataset 2 | MapSplice | 10 | 20 | 2 | Rmsk | 5.79 ± 6.57E-1 | 5.43 ± 6.04E-1 | 6.35 ± 8.27E-1 |
| dataset 2 | MapSplice | 10 | 20 | 2 | Not in Rmsk | 13.65 ± 7.96E-1 | 11.44 ± 7.39E-1 | 16.19 ± 1.98E-1 |
| dataset 2 | MapSplice | 10 | 20 | 2 | Total | 11.79 ± 6.41E-1 | 10.28 ± 6.52E-1 | 12.84 ± 2.39E-1 |
| dataset 2 | RUM | 10 | 20 | 2 | Rmsk | 12.91 ± 3.28E-1 | 9.42 ± 2.94E-1 | 27.06 ± 4.60E-1 |
| dataset 2 | RUM | 10 | 20 | 2 | Not in Rmsk | 10.48 ± 8.67E-1 | 7.02 ± 7.71E-1 | 33.03 ± 4.22E-1 |
| dataset 2 | RUM | 10 | 20 | 2 | Total | 11.09 ± 1.26E-1 | 7.51 ± 7.60E-1 | 32.28 ± 2.77E-1 |
| dataset 2 | Tophat2 | 10 | 20 | 2 | Rmsk | 8.94 ± 9.29E-1 | 8.62 ± 1.41E-1 | 3.62 ± 2.34E1 |
| dataset 2 | Tophat2 | 10 | 20 | 2 | Not in Rmsk | 6.04 ± 5.54E-1 | 5.92 ± 4.52E-1 | 1.90 ± 1.52E-1 |
| dataset 2 | Tophat2 | 10 | 20 | 2 | Total | 6.40 ± 5.88E-1 | 6.19 ± 2.69E-1 | 3.25 ± 4.72E-1 |
| dataset 2 | GSNAP | 20 | 10 | 2 | Rmsk | 7.04 ± 1.79E-1 | 6.90 ± 1.93E-1 | 1.92 ± 7.37E-1 |
| dataset 2 | GSNAP | 20 | 10 | 2 | Not in Rmsk | 4.83 ± 8.37E-1 | 4.26 ± 4.87E-1 | 11.80 ± 7.44E-1 |
| dataset 2 | GSNAP | 20 | 10 | 2 | Total | 5.26 ± 9.20E-1 | 4.67 ± 6.06E-1 | 11.18 ± 6.04E-1 |
| dataset 2 | MapSplice | 20 | 10 | 2 | Rmsk | 11.68 ± 1.54E-1 | 11.11 ± 2.81E-1 | 4.82 ± 1.16E1 |
| dataset 2 | MapSplice | 20 | 10 | 2 | Not in Rmsk | 14.36 ± 8.40E-1 | 12.65 ± 8.96E-1 | 11.86 ± 1.96E-1 |
| dataset 2 | MapSplice | 20 | 10 | 2 | Total | 13.85 ± 7.34E-1 | 12.42 ± 9.50E-1 | 10.35 ± 2.94E-1 |
| dataset 2 | RUM | 20 | 10 | 2 | Rmsk | 25.42 ± 4.92E-1 | 21.48 ± 6.82E-1 | 15.50 ± 1.11E1 |
| dataset 2 | RUM | 20 | 10 | 2 | Not in Rmsk | 16.60 ± 2.10E-1 | 12.93 ± 1.75E-1 | 22.13 ± 1.07E-1 |
| dataset 2 | RUM | 20 | 10 | 2 | Total | 18.38 ± 2.06E-1 | 14.33 ± 2.05E-1 | 22.07 ± 2.44E-1 |
| dataset 2 | Tophat2 | 20 | 10 | 2 | Rmsk | 18.00 ± 7.40E-1 | 17.56 ± 1.17E1 | 2.52 ± 2.83E1 |
| dataset 2 | Tophat2 | 20 | 10 | 2 | Not in Rmsk | 13.79 ± 2.77E-1 | 13.62 ± 2.80E-1 | 1.24 ± 5.21E-1 |
| dataset 2 | Tophat2 | 20 | 10 | 2 | Total | 14.18 ± 2.99E-1 | 13.92 ± 3.06E-1 | 1.86 ± 2.15E-1 |
| dataset 2 | GSNAP | 20 | 20 | 4 | Rmsk | 1.98 ± 1.56E-1 | 1.88 ± 1.72E-1 | 5.57 ± 1.32E1 |
| dataset 2 | GSNAP | 20 | 20 | 4 | Not in Rmsk | 2.02 ± 1.17E-1 | 1.48 ± 6.51E-1 | 26.99 ± 1.02E1 |
| dataset 2 | GSNAP | 20 | 20 | 4 | Total | 2.02 ± 1.19E-1 | 1.54 ± 7.69E-1 | 23.70 ± 6.75E-1 |
| dataset 2 | MapSplice | 20 | 20 | 4 | Rmsk | 2.61 ± 1.37E-1 | 2.31 ± 1.80E-1 | 11.58 ± 3.14E1 |
| dataset 2 | MapSplice | 20 | 20 | 4 | Not in Rmsk | 8.25 ± 5.59E-1 | 6.29 ± 2.23E-1 | 23.76 ± 2.78E-1 |
| dataset 2 | MapSplice | 20 | 20 | 4 | Total | 7.22 ± 2.86E-1 | 5.70 ± 1.09E-1 | 21.05 ± 4.61E-1 |
| dataset 2 | RUM | 20 | 20 | 4 | Rmsk | 10.65 ± 5.39E-1 | 7.93 ± 6.18E-1 | 25.55 ± 2.02E1 |
| dataset 2 | RUM | 20 | 20 | 4 | Not in Rmsk | 8.18 ± 1.01E-1 | 4.92 ± 8.25E-1 | 39.93 ± 3.58E-1 |
| dataset 2 | RUM | 20 | 20 | 4 | Total | 8.65 ± 1.33E-1 | 5.38 ± 1.07E-1 | 37.79 ± 3.07E-1 |
| dataset 2 | Tophat2 | 20 | 20 | 4 | Rmsk | 4.40 ± 2.97E-1 | 4.17 ± 3.36E-1 | 5.16 ± 6.49E1 |
| dataset 2 | Tophat2 | 20 | 20 | 4 | Not in Rmsk | 2.39 ± 5.39E-1 | 2.27 ± 5.34E-1 | 4.95 ± 4.54E-1 |
| dataset 2 | Tophat2 | 20 | 20 | 4 | Total | 2.57 ± 6.38E-1 | 2.41 ± 7.25E-1 | 6.19 ± 1.25E1 |

# Table S14. Effect of removing RNA-DNA sequence differences in pseudogenes on the false discovery rate of sequence difference detection

| **Dataset** | **Aligner** | **Minimum Coverage** | **Minimum Level** | **Minimum RDD Count** | **FDR Before Pseudogene Filter (%)** | **FDR After Pseudogene Filter (%)** | **Percent Decrease in FDR (%)** |
| --- | --- | --- | --- | --- | --- | --- | --- |
| dataset 1 | GSNAP | 10 | 10 | 2 | 1.92 ± 5.86E-1 | 0.95 ± 5.15E-1 | 50.72 ± 1.41E1 |
| dataset 1 | MapSplice | 10 | 10 | 2 | 6.22 ± 8.01E-1 | 4.54 ± 3.92E-1 | 26.94 ± 3.16E-1 |
| dataset 1 | RUM | 10 | 10 | 2 | 5.45 ± 9.96E-1 | 2.35 ± 9.18E-1 | 56.92 ± 9.42E-1 |
| dataset 1 | Tophat2 | 10 | 10 | 2 | 1.26 ± 4.50E-1 | 0.85 ± 3.48E-1 | 32.50 ± 1.62E1 |
| dataset 1 | GSNAP | 10 | 20 | 2 | 1.61 ± 6.15E-1 | 0.74 ± 4.28E-1 | 53.89 ± 1.14E1 |
| dataset 1 | MapSplice | 10 | 20 | 2 | 6.27 ± 9.16E-1 | 4.65 ± 3.54E-1 | 25.93 ± 5.49E-1 |
| dataset 1 | RUM | 10 | 20 | 2 | 4.50 ± 9.07E-1 | 1.67 ± 6.58E-1 | 62.98 ± 8.05E-1 |
| dataset 1 | Tophat2 | 10 | 20 | 2 | 0.88 ± 3.24E-1 | 0.57 ± 2.16E-1 | 35.23 ± 1.07E1 |
| dataset 1 | GSNAP | 20 | 10 | 2 | 1.55 ± 4.67E-1 | 0.79 ± 4.74E-1 | 49.18 ± 1.79E1 |
| dataset 1 | MapSplice | 20 | 10 | 2 | 4.02 ± 8.09E-1 | 2.57 ± 3.26E-1 | 36.13 ± 4.79E-1 |
| dataset 1 | RUM | 20 | 10 | 2 | 5.00 ± 1.09E-1 | 2.24 ± 4.59E-1 | 55.27 ± 1.86E1 |
| dataset 1 | Tophat2 | 20 | 10 | 2 | 1.23 ± 3.59E-1 | 0.87 ± 4.24E-1 | 28.83 ± 1.74E1 |
| dataset 1 | GSNAP | 20 | 20 | 4 | 1.23 ± 4.68E-1 | 0.58 ± 3.57E-1 | 52.47 ± 1.73E1 |
| dataset 1 | MapSplice | 20 | 20 | 4 | 3.86 ± 9.92E-1 | 2.47 ± 3.21E-1 | 36.08 ± 8.77E-1 |
| dataset 1 | RUM | 20 | 20 | 4 | 3.95 ± 9.39E-1 | 1.50 ± 3.12E-1 | 62.01 ± 1.70E1 |
| dataset 1 | Tophat2 | 20 | 20 | 4 | 0.83 ± 2.63E-1 | 0.58 ± 2.94E-1 | 30.62 ± 1.48E1 |
| dataset 2 | GSNAP | 10 | 10 | 2 | 14.94 ± 1.04E-1 | 13.90 ± 1.09E-1 | 6.99 ± 1.61E-1 |
| dataset 2 | MapSplice | 10 | 10 | 2 | 28.89 ± 5.26E-1 | 27.26 ± 4.39E-1 | 5.65 ± 4.16E-1 |
| dataset 2 | RUM | 10 | 10 | 2 | 29.87 ± 2.39E-1 | 25.21 ± 2.79E-1 | 15.61 ± 2.59E-1 |
| dataset 2 | Tophat2 | 10 | 10 | 2 | 32.94 ± 1.33E-1 | 32.62 ± 1.41E-1 | 0.96 ± 3.40E-1 |
| dataset 2 | GSNAP | 10 | 20 | 2 | 3.46 ± 7.06E-1 | 2.39 ± 5.53E-1 | 30.91 ± 3.90E-1 |
| dataset 2 | MapSplice | 10 | 20 | 2 | 11.79 ± 6.41E-1 | 9.60 ± 4.67E-1 | 18.60 ± 1.28E-1 |
| dataset 2 | RUM | 10 | 20 | 2 | 11.09 ± 1.26E-1 | 7.39 ± 1.62E-1 | 33.40 ± 7.48E-1 |
| dataset 2 | Tophat2 | 10 | 20 | 2 | 6.40 ± 5.88E-1 | 5.98 ± 6.18E-1 | 6.58 ± 1.67E-1 |
| dataset 2 | GSNAP | 20 | 10 | 2 | 5.26 ± 9.20E-1 | 4.19 ± 4.68E-1 | 20.45 ± 9.56E-1 |
| dataset 2 | MapSplice | 20 | 10 | 2 | 13.85 ± 7.34E-1 | 11.92 ± 7.96E-1 | 13.93 ± 1.45E-1 |
| dataset 2 | RUM | 20 | 10 | 2 | 18.38 ± 2.06E-1 | 13.44 ± 2.09E-1 | 26.89 ± 4.11E-1 |
| dataset 2 | Tophat2 | 20 | 10 | 2 | 14.18 ± 2.99E-1 | 13.62 ± 3.60E-1 | 3.97 ± 5.28E-1 |
| dataset 2 | GSNAP | 20 | 20 | 4 | 2.02 ± 1.19E-1 | 1.10 ± 4.80E-1 | 45.32 ± 1.81E1 |
| dataset 2 | MapSplice | 20 | 20 | 4 | 7.22 ± 2.86E-1 | 5.16 ± 3.39E-1 | 28.60 ± 1.87E-1 |
| dataset 2 | RUM | 20 | 20 | 4 | 8.65 ± 1.33E-1 | 5.04 ± 1.35E-1 | 41.72 ± 7.01E-1 |
| dataset 2 | Tophat2 | 20 | 20 | 4 | 2.57 ± 6.38E-1 | 2.08 ± 6.15E-1 | 19.01 ± 1.00E1 |

# Table S15. Effect of removing RDDs near exon junctions on the false discovery rate of sequence difference detection.

| **Dataset** | **Aligner** | **Minimum Coverage** | **Minimum Level** | **Minimum RDD Count** | **% of False Positives near Junctions** | **FDR before filter (%)** | **FDR after filter (%)** | **Percent FDR decrease (%)** |
| --- | --- | --- | --- | --- | --- | --- | --- | --- |
| dataset 1 | GSNAP | 10 | 10 | 2 | 2.64 ± 1.92E-1 | 1.92 ± 5.86E-1 | 1.88 ± 6.03E-1 | 2.34 ± 1.91E-1 |
| dataset 1 | MapSplice | 10 | 10 | 2 | 54.72 ± 4.44E-1 | 6.22 ± 8.01E-1 | 2.92 ± 6.65E-1 | 53.00 ± 4.68E-1 |
| dataset 1 | RUM | 10 | 10 | 2 | 1.12 ± 3.04E-1 | 5.45 ± 9.96E-1 | 5.41 ± 9.97E-1 | 0.82 ± 2.19E-1 |
| dataset 1 | Tophat2 | 10 | 10 | 2 | 0.55 ± 1.45E-1 | 1.26 ± 4.50E-1 | 1.25 ± 4.32E-1 | 0.24 ± 1.46E-1 |
| dataset 1 | GSNAP | 10 | 20 | 2 | 3.20 ± 2.37E-1 | 1.61 ± 6.15E-1 | 1.57 ± 6.31E-1 | 2.90 ± 2.35E-1 |
| dataset 1 | MapSplice | 10 | 20 | 2 | 59.44 ± 6.70E-1 | 6.27 ± 9.16E-1 | 2.65 ± 8.27E-1 | 57.76 ± 7.03E-1 |
| dataset 1 | RUM | 10 | 20 | 2 | 1.35 ± 4.12E-1 | 4.50 ± 9.07E-1 | 4.46 ± 8.92E-1 | 1.05 ± 3.50E-1 |
| dataset 1 | Tophat2 | 10 | 20 | 2 | 0.55 ± 1.84E-1 | 0.88 ± 3.24E-1 | 0.87 ± 3.17E-1 | 0.24 ± 1.84E-1 |
| dataset 1 | GSNAP | 20 | 10 | 2 | 1.85 ± 1.89E-1 | 1.55 ± 4.67E-1 | 1.53 ± 4.75E-1 | 1.56 ± 1.91E-1 |
| dataset 1 | MapSplice | 20 | 10 | 2 | 39.13 ± 3.06E-1 | 4.02 ± 8.09E-1 | 2.49 ± 6.06E-1 | 37.99 ± 3.22E-1 |
| dataset 1 | RUM | 20 | 10 | 2 | 0.72 ± 9.42E-1 | 5.00 ± 1.09E-1 | 4.98 ± 1.13E-1 | 0.43 ± 7.95E-1 |
| dataset 1 | Tophat2 | 20 | 10 | 2 | 0.66 ± 6.24E-1 | 1.23 ± 3.59E-1 | 1.22 ± 3.53E-1 | 0.32 ± 7.06E-1 |
| dataset 1 | GSNAP | 20 | 20 | 4 | 2.29 ± 2.27E-1 | 1.23 ± 4.68E-1 | 1.20 ± 4.68E-1 | 2.01 ± 2.24E-1 |
| dataset 1 | MapSplice | 20 | 20 | 4 | 44.27 ± 6.59E-1 | 3.86 ± 9.92E-1 | 2.19 ± 8.16E-1 | 43.15 ± 6.84E-1 |
| dataset 1 | RUM | 20 | 20 | 4 | 0.83 ± 1.23E-1 | 3.95 ± 9.39E-1 | 3.92 ± 9.73E-1 | 0.54 ± 1.06E-1 |
| dataset 1 | Tophat2 | 20 | 20 | 4 | 0.61 ± 6.62E-1 | 0.83 ± 2.63E-1 | 0.83 ± 2.67E-1 | 0.28 ± 6.46E-1 |
| dataset 2 | GSNAP | 10 | 10 | 2 | 1.47 ± 6.10E-1 | 14.94 ± 1.04E-1 | 14.80 ± 1.08E-1 | 0.98 ± 5.64E-1 |
| dataset 2 | MapSplice | 10 | 10 | 2 | 17.66 ± 5.19E-1 | 28.89 ± 5.26E-1 | 25.13 ± 3.47E-1 | 13.02 ± 4.33E-1 |
| dataset 2 | RUM | 10 | 10 | 2 | 1.20 ± 5.56E-1 | 29.87 ± 2.39E-1 | 29.69 ± 2.28E-1 | 0.62 ± 4.03E-1 |
| dataset 2 | Tophat2 | 10 | 10 | 2 | 0.41 ± 2.97E-1 | 32.94 ± 1.33E-1 | 32.93 ± 1.42E-1 | 0.03 ± 3.41E-1 |
| dataset 2 | GSNAP | 10 | 20 | 2 | 4.50 ± 2.42E-1 | 3.46 ± 7.06E-1 | 3.32 ± 7.56E-1 | 4.06 ± 2.26E-1 |
| dataset 2 | MapSplice | 10 | 20 | 2 | 44.32 ± 2.71E-1 | 11.79 ± 6.41E-1 | 6.95 ± 6.81E-1 | 41.07 ± 2.73E-1 |
| dataset 2 | RUM | 10 | 20 | 2 | 2.53 ± 1.49E-1 | 11.09 ± 1.26E-1 | 10.87 ± 1.09E-1 | 1.97 ± 1.41E-1 |
| dataset 2 | Tophat2 | 10 | 20 | 2 | 0.57 ± 1.44E-1 | 6.40 ± 5.88E-1 | 6.39 ± 5.18E-1 | 0.19 ± 1.49E-1 |
| dataset 2 | GSNAP | 20 | 10 | 2 | 2.42 ± 1.05E-1 | 5.26 ± 9.20E-1 | 5.16 ± 9.15E-1 | 1.98 ± 8.61E-1 |
| dataset 2 | MapSplice | 20 | 10 | 2 | 27.15 ± 1.11E-1 | 13.85 ± 7.34E-1 | 10.52 ± 6.15E-1 | 24.08 ± 1.01E-1 |
| dataset 2 | RUM | 20 | 10 | 2 | 1.39 ± 6.86E-1 | 18.38 ± 2.06E-1 | 18.23 ± 2.04E-1 | 0.86 ± 5.62E-1 |
| dataset 2 | Tophat2 | 20 | 10 | 2 | 0.50 ± 3.89E-1 | 14.18 ± 2.99E-1 | 14.16 ± 2.95E-1 | 0.11 ± 2.65E-1 |
| dataset 2 | GSNAP | 20 | 20 | 4 | 4.47 ± 4.56E-1 | 2.02 ± 1.19E-1 | 1.93 ± 1.17E-1 | 4.08 ± 4.34E-1 |
| dataset 2 | MapSplice | 20 | 20 | 4 | 43.17 ± 4.43E-1 | 7.22 ± 2.86E-1 | 4.25 ± 4.01E-1 | 41.15 ± 4.29E-1 |
| dataset 2 | RUM | 20 | 20 | 4 | 2.02 ± 1.17E-1 | 8.65 ± 1.33E-1 | 8.51 ± 1.20E-1 | 1.54 ± 1.22E-1 |
| dataset 2 | Tophat2 | 20 | 20 | 4 | 0.84 ± 6.13E-1 | 2.57 ± 6.38E-1 | 2.56 ± 6.04E-1 | 0.46 ± 5.98E-1 |

# Table S16. Percentage of true versus false positives removed by requiring concordance with at least one other aligner, BLAT filter, pseudogene filter, and removal of intronic sites within 6bp of exon junctions used in conjunction.

| **Dataset** | **Aligner** | **Minimum Coverage** | **Minimum Level** | **Minimum RDD Count** | **Region** | **Percent of True Positives Removed** | **Percent of False Positives Removed** |
| --- | --- | --- | --- | --- | --- | --- | --- |
| dataset 1 | GSNAP | 10 | 10 | 2 | Rmsk | 23.80 ± 0.34 | 62.66 ± 3.58 |
| dataset 1 | GSNAP | 10 | 10 | 2 | Not in Rmsk | 3.53 ± 0.00 | 66.88 ± 0.77 |
| dataset 1 | GSNAP | 10 | 10 | 2 | Total | 4.97 ± 0.04 | 66.57 ± 0.83 |
| dataset 1 | MapSplice | 10 | 10 | 2 | Rmsk | 22.79 ± 0.31 | 69.82 ± 3.04 |
| dataset 1 | MapSplice | 10 | 10 | 2 | Not in Rmsk | 2.96 ± 0.06 | 90.28 ± 0.08 |
| dataset 1 | MapSplice | 10 | 10 | 2 | Total | 4.36 ± 0.10 | 89.70 ± 0.02 |
| dataset 1 | RUM | 10 | 10 | 2 | Rmsk | 22.44 ± 0.26 | 85.07 ± 2.17 |
| dataset 1 | RUM | 10 | 10 | 2 | Not in Rmsk | 2.73 ± 0.03 | 85.19 ± 0.26 |
| dataset 1 | RUM | 10 | 10 | 2 | Total | 4.10 ± 0.06 | 85.19 ± 0.36 |
| dataset 1 | Tophat2 | 10 | 10 | 2 | Rmsk | 20.89 ± 0.57 | 53.29 ± 2.21 |
| dataset 1 | Tophat2 | 10 | 10 | 2 | Not in Rmsk | 2.00 ± 0.01 | 49.56 ± 1.13 |
| dataset 1 | Tophat2 | 10 | 10 | 2 | Total | 3.12 ± 0.03 | 49.82 ± 1.06 |
| dataset 1 | GSNAP | 10 | 20 | 2 | Rmsk | 23.96 ± 0.42 | 64.32 ± 5.17 |
| dataset 1 | GSNAP | 10 | 20 | 2 | Not in Rmsk | 3.73 ± 0.04 | 71.39 ± 0.88 |
| dataset 1 | GSNAP | 10 | 20 | 2 | Total | 5.17 ± 0.04 | 70.89 ± 1.01 |
| dataset 1 | MapSplice | 10 | 20 | 2 | Rmsk | 22.94 ± 0.38 | 71.87 ± 3.32 |
| dataset 1 | MapSplice | 10 | 20 | 2 | Not in Rmsk | 3.16 ± 0.05 | 93.07 ± 0.17 |
| dataset 1 | MapSplice | 10 | 20 | 2 | Total | 4.56 ± 0.10 | 92.55 ± 0.17 |
| dataset 1 | RUM | 10 | 20 | 2 | Rmsk | 22.29 ± 0.25 | 85.19 ± 2.70 |
| dataset 1 | RUM | 10 | 20 | 2 | Not in Rmsk | 2.67 ± 0.03 | 87.57 ± 0.30 |
| dataset 1 | RUM | 10 | 20 | 2 | Total | 4.03 ± 0.06 | 87.40 ± 0.34 |
| dataset 1 | Tophat2 | 10 | 20 | 2 | Rmsk | 20.88 ± 0.70 | 55.43 ± 0.42 |
| dataset 1 | Tophat2 | 10 | 20 | 2 | Not in Rmsk | 2.12 ± 0.03 | 52.91 ± 0.49 |
| dataset 1 | Tophat2 | 10 | 20 | 2 | Total | 3.23 ± 0.03 | 53.09 ± 0.47 |
| dataset 1 | GSNAP | 20 | 10 | 2 | Rmsk | 24.43 ± 0.36 | 57.82 ± 1.15 |
| dataset 1 | GSNAP | 20 | 10 | 2 | Not in Rmsk | 3.83 ± 0.05 | 61.80 ± 0.76 |
| dataset 1 | GSNAP | 20 | 10 | 2 | Total | 5.20 ± 0.08 | 61.53 ± 0.67 |
| dataset 1 | MapSplice | 20 | 10 | 2 | Rmsk | 23.12 ± 0.28 | 66.46 ± 3.47 |
| dataset 1 | MapSplice | 20 | 10 | 2 | Not in Rmsk | 3.06 ± 0.07 | 85.53 ± 0.17 |
| dataset 1 | MapSplice | 20 | 10 | 2 | Total | 4.39 ± 0.11 | 84.94 ± 0.26 |
| dataset 1 | RUM | 20 | 10 | 2 | Rmsk | 22.18 ± 0.23 | 86.50 ± 2.74 |
| dataset 1 | RUM | 20 | 10 | 2 | Not in Rmsk | 2.60 ± 0.05 | 84.83 ± 0.31 |
| dataset 1 | RUM | 20 | 10 | 2 | Total | 3.87 ± 0.08 | 84.97 ± 0.48 |
| dataset 1 | Tophat2 | 20 | 10 | 2 | Rmsk | 19.00 ± 0.72 | 51.49 ± 3.06 |
| dataset 1 | Tophat2 | 20 | 10 | 2 | Not in Rmsk | 1.87 ± 0.03 | 45.14 ± 2.02 |
| dataset 1 | Tophat2 | 20 | 10 | 2 | Total | 2.77 ± 0.03 | 45.58 ± 1.66 |
| dataset 1 | GSNAP | 20 | 20 | 4 | Rmsk | 24.60 ± 0.39 | 57.72 ± 3.02 |
| dataset 1 | GSNAP | 20 | 20 | 4 | Not in Rmsk | 4.07 ± 0.04 | 66.04 ± 1.03 |
| dataset 1 | GSNAP | 20 | 20 | 4 | Total | 5.43 ± 0.07 | 65.50 ± 1.08 |
| dataset 1 | MapSplice | 20 | 20 | 4 | Rmsk | 23.34 ± 0.40 | 67.44 ± 4.19 |
| dataset 1 | MapSplice | 20 | 20 | 4 | Not in Rmsk | 3.31 ± 0.08 | 89.48 ± 0.18 |
| dataset 1 | MapSplice | 20 | 20 | 4 | Total | 4.63 ± 0.13 | 88.90 ± 0.25 |
| dataset 1 | RUM | 20 | 20 | 4 | Rmsk | 22.02 ± 0.27 | 86.32 ± 3.72 |
| dataset 1 | RUM | 20 | 20 | 4 | Not in Rmsk | 2.53 ± 0.05 | 86.96 ± 0.32 |
| dataset 1 | RUM | 20 | 20 | 4 | Total | 3.79 ± 0.09 | 86.94 ± 0.52 |
| dataset 1 | Tophat2 | 20 | 20 | 4 | Rmsk | 18.84 ± 0.66 | 54.81 ± 1.19 |
| dataset 1 | Tophat2 | 20 | 20 | 4 | Not in Rmsk | 1.96 ± 0.03 | 47.41 ± 0.40 |
| dataset 1 | Tophat2 | 20 | 20 | 4 | Total | 2.85 ± 0.04 | 47.92 ± 0.40 |
| dataset 2 | GSNAP | 10 | 10 | 2 | Rmsk | 29.91 ± 0.37 | 34.95 ± 0.20 |
| dataset 2 | GSNAP | 10 | 10 | 2 | Not in Rmsk | 5.11 ± 0.05 | 22.98 ± 0.32 |
| dataset 2 | GSNAP | 10 | 10 | 2 | Total | 11.38 ± 0.06 | 26.96 ± 0.28 |
| dataset 2 | MapSplice | 10 | 10 | 2 | Rmsk | 28.17 ± 0.42 | 53.20 ± 0.19 |
| dataset 2 | MapSplice | 10 | 10 | 2 | Not in Rmsk | 3.64 ± 0.03 | 61.19 ± 0.17 |
| dataset 2 | MapSplice | 10 | 10 | 2 | Total | 9.81 ± 0.05 | 59.12 ± 0.16 |
| dataset 2 | RUM | 10 | 10 | 2 | Rmsk | 26.73 ± 0.34 | 67.01 ± 0.40 |
| dataset 2 | RUM | 10 | 10 | 2 | Not in Rmsk | 3.33 ± 0.09 | 58.85 ± 0.14 |
| dataset 2 | RUM | 10 | 10 | 2 | Total | 9.07 ± 0.06 | 61.68 ± 0.17 |
| dataset 2 | Tophat2 | 10 | 10 | 2 | Rmsk | 25.90 ± 0.52 | 95.13 ± 0.19 |
| dataset 2 | Tophat2 | 10 | 10 | 2 | Not in Rmsk | 2.61 ± 0.09 | 94.91 ± 0.09 |
| dataset 2 | Tophat2 | 10 | 10 | 2 | Total | 5.46 ± 0.08 | 94.95 ± 0.05 |
| dataset 2 | GSNAP | 10 | 20 | 2 | Rmsk | 30.05 ± 0.31 | 47.74 ± 1.42 |
| dataset 2 | GSNAP | 10 | 20 | 2 | Not in Rmsk | 5.35 ± 0.08 | 58.29 ± 0.50 |
| dataset 2 | GSNAP | 10 | 20 | 2 | Total | 11.61 ± 0.08 | 55.60 ± 0.27 |
| dataset 2 | MapSplice | 10 | 20 | 2 | Rmsk | 28.30 ± 0.32 | 62.66 ± 0.53 |
| dataset 2 | MapSplice | 10 | 20 | 2 | Not in Rmsk | 3.81 ± 0.04 | 88.56 ± 0.11 |
| dataset 2 | MapSplice | 10 | 20 | 2 | Total | 10.00 ± 0.04 | 85.55 ± 0.07 |
| dataset 2 | RUM | 10 | 20 | 2 | Rmsk | 26.54 ± 0.27 | 83.96 ± 0.40 |
| dataset 2 | RUM | 10 | 20 | 2 | Not in Rmsk | 3.20 ± 0.06 | 84.82 ± 0.26 |
| dataset 2 | RUM | 10 | 20 | 2 | Total | 8.95 ± 0.04 | 84.57 ± 0.07 |
| dataset 2 | Tophat2 | 10 | 20 | 2 | Rmsk | 25.79 ± 0.45 | 86.42 ± 0.93 |
| dataset 2 | Tophat2 | 10 | 20 | 2 | Not in Rmsk | 2.68 ± 0.03 | 87.26 ± 0.57 |
| dataset 2 | Tophat2 | 10 | 20 | 2 | Total | 5.51 ± 0.06 | 87.11 ± 0.47 |
| dataset 2 | GSNAP | 20 | 10 | 2 | Rmsk | 30.96 ± 0.67 | 46.70 ± 0.61 |
| dataset 2 | GSNAP | 20 | 10 | 2 | Not in Rmsk | 6.24 ± 0.01 | 46.48 ± 1.49 |
| dataset 2 | GSNAP | 20 | 10 | 2 | Total | 10.99 ± 0.12 | 46.54 ± 1.13 |
| dataset 2 | MapSplice | 20 | 10 | 2 | Rmsk | 28.95 ± 0.59 | 64.06 ± 0.12 |
| dataset 2 | MapSplice | 20 | 10 | 2 | Not in Rmsk | 4.33 ± 0.07 | 80.45 ± 0.29 |
| dataset 2 | MapSplice | 20 | 10 | 2 | Total | 9.05 ± 0.07 | 77.87 ± 0.26 |
| dataset 2 | RUM | 20 | 10 | 2 | Rmsk | 26.57 ± 0.56 | 85.72 ± 0.24 |
| dataset 2 | RUM | 20 | 10 | 2 | Not in Rmsk | 3.31 ± 0.10 | 83.84 ± 0.24 |
| dataset 2 | RUM | 20 | 10 | 2 | Total | 7.61 ± 0.12 | 84.37 ± 0.11 |
| dataset 2 | Tophat2 | 20 | 10 | 2 | Rmsk | 23.98 ± 0.99 | 87.18 ± 0.13 |
| dataset 2 | Tophat2 | 20 | 10 | 2 | Not in Rmsk | 2.64 ± 0.11 | 89.84 ± 0.37 |
| dataset 2 | Tophat2 | 20 | 10 | 2 | Total | 4.54 ± 0.18 | 89.53 ± 0.33 |
| dataset 2 | GSNAP | 20 | 20 | 4 | Rmsk | 31.20 ± 0.58 | 61.86 ± 1.43 |
| dataset 2 | GSNAP | 20 | 20 | 4 | Not in Rmsk | 6.58 ± 0.05 | 71.82 ± 1.47 |
| dataset 2 | GSNAP | 20 | 20 | 4 | Total | 11.30 ± 0.13 | 69.96 ± 1.21 |
| dataset 2 | MapSplice | 20 | 20 | 4 | Rmsk | 29.30 ± 0.48 | 69.78 ± 1.09 |
| dataset 2 | MapSplice | 20 | 20 | 4 | Not in Rmsk | 4.60 ± 0.07 | 92.90 ± 0.14 |
| dataset 2 | MapSplice | 20 | 20 | 4 | Total | 9.34 ± 0.04 | 91.37 ± 0.15 |
| dataset 2 | RUM | 20 | 20 | 4 | Rmsk | 26.30 ± 0.44 | 92.87 ± 0.17 |
| dataset 2 | RUM | 20 | 20 | 4 | Not in Rmsk | 3.16 ± 0.09 | 92.58 ± 0.27 |
| dataset 2 | RUM | 20 | 20 | 4 | Total | 7.43 ± 0.09 | 92.65 ± 0.17 |
| dataset 2 | Tophat2 | 20 | 20 | 4 | Rmsk | 23.85 ± 1.18 | 64.97 ± 5.45 |
| dataset 2 | Tophat2 | 20 | 20 | 4 | Not in Rmsk | 2.68 ± 0.01 | 65.05 ± 1.84 |
| dataset 2 | Tophat2 | 20 | 20 | 4 | Total | 4.55 ± 0.11 | 65.07 ± 0.76 |

# Table S17. Combined effect of requiring concordance with at least one other aligner, BLAT filter, pseudogene filter, and removal of intronic sites within 6bp of exon junctions on the false discovery rate of sequence difference detection.

| **Dataset** | **Aligner** | **Minimum Coverage** | **Minimum Level** | **Minimum RDD Count** | **Region** | **FDR Before BLAT Filter (%)** | **FDR After BLAT Filter (%)** | **Percent Decrease in FDR (%)** |
| --- | --- | --- | --- | --- | --- | --- | --- | --- |
| dataset 1 | GSNAP | 10 | 10 | 2 | Rmsk | 2.02 ± 0.18 | 0.99 ± 0.05 | 50.47 ± 4.87 |
| dataset 1 | GSNAP | 10 | 10 | 2 | Not in Rmsk | 1.92 ± 0.05 | 0.67 ± 0.00 | 65.23 ± 0.79 |
| dataset 1 | GSNAP | 10 | 10 | 2 | Total | 1.92 ± 0.06 | 0.69 ± 0.01 | 64.38 ± 0.87 |
| dataset 1 | MapSplice | 10 | 10 | 2 | Rmsk | 2.61 ± 0.18 | 1.03 ± 0.03 | 60.27 ± 4.08 |
| dataset 1 | MapSplice | 10 | 10 | 2 | Not in Rmsk | 6.48 ± 0.07 | 0.69 ± 0.01 | 89.36 ± 0.09 |
| dataset 1 | MapSplice | 10 | 10 | 2 | Total | 6.22 ± 0.08 | 0.71 ± 0.01 | 88.60 ± 0.02 |
| dataset 1 | RUM | 10 | 10 | 2 | Rmsk | 6.06 ± 0.71 | 1.21 ± 0.04 | 79.76 ± 2.85 |
| dataset 1 | RUM | 10 | 10 | 2 | Not in Rmsk | 5.41 ± 0.06 | 0.86 ± 0.01 | 84.04 ± 0.27 |
| dataset 1 | RUM | 10 | 10 | 2 | Total | 5.45 ± 0.10 | 0.88 ± 0.01 | 83.81 ± 0.39 |
| dataset 1 | Tophat2 | 10 | 10 | 2 | Rmsk | 1.43 ± 0.17 | 0.85 ± 0.07 | 40.62 ± 2.51 |
| dataset 1 | Tophat2 | 10 | 10 | 2 | Not in Rmsk | 1.24 ± 0.04 | 0.64 ± 0.03 | 48.22 ± 1.16 |
| dataset 1 | Tophat2 | 10 | 10 | 2 | Total | 1.26 ± 0.04 | 0.65 ± 0.03 | 47.89 ± 1.10 |
| dataset 1 | GSNAP | 10 | 20 | 2 | Rmsk | 1.68 ± 0.20 | 0.79 ± 0.04 | 52.64 ± 7.00 |
| dataset 1 | GSNAP | 10 | 20 | 2 | Not in Rmsk | 1.61 ± 0.05 | 0.48 ± 0.01 | 69.94 ± 0.92 |
| dataset 1 | GSNAP | 10 | 20 | 2 | Total | 1.61 ± 0.06 | 0.50 ± 0.00 | 68.95 ± 1.07 |
| dataset 1 | MapSplice | 10 | 20 | 2 | Rmsk | 2.28 ± 0.13 | 0.84 ± 0.05 | 62.95 ± 4.48 |
| dataset 1 | MapSplice | 10 | 20 | 2 | Not in Rmsk | 6.56 ± 0.08 | 0.50 ± 0.01 | 92.38 ± 0.18 |
| dataset 1 | MapSplice | 10 | 20 | 2 | Total | 6.27 ± 0.09 | 0.52 ± 0.01 | 91.72 ± 0.18 |
| dataset 1 | RUM | 10 | 20 | 2 | Rmsk | 4.97 ± 0.73 | 0.97 ± 0.07 | 80.15 ± 3.54 |
| dataset 1 | RUM | 10 | 20 | 2 | Not in Rmsk | 4.47 ± 0.06 | 0.59 ± 0.01 | 86.71 ± 0.31 |
| dataset 1 | RUM | 10 | 20 | 2 | Total | 4.50 ± 0.09 | 0.62 ± 0.01 | 86.34 ± 0.37 |
| dataset 1 | Tophat2 | 10 | 20 | 2 | Rmsk | 1.05 ± 0.13 | 0.59 ± 0.08 | 43.41 ± 1.02 |
| dataset 1 | Tophat2 | 10 | 20 | 2 | Not in Rmsk | 0.87 ± 0.03 | 0.42 ± 0.01 | 51.68 ± 0.50 |
| dataset 1 | Tophat2 | 10 | 20 | 2 | Total | 0.88 ± 0.03 | 0.43 ± 0.01 | 51.30 ± 0.47 |
| dataset 1 | GSNAP | 20 | 10 | 2 | Rmsk | 1.58 ± 0.12 | 0.89 ± 0.08 | 43.79 ± 1.79 |
| dataset 1 | GSNAP | 20 | 10 | 2 | Not in Rmsk | 1.55 ± 0.04 | 0.62 ± 0.01 | 59.91 ± 0.80 |
| dataset 1 | GSNAP | 20 | 10 | 2 | Total | 1.55 ± 0.05 | 0.64 ± 0.01 | 59.05 ± 0.71 |
| dataset 1 | MapSplice | 20 | 10 | 2 | Rmsk | 1.95 ± 0.11 | 0.86 ± 0.06 | 55.88 ± 4.66 |
| dataset 1 | MapSplice | 20 | 10 | 2 | Not in Rmsk | 4.16 ± 0.08 | 0.64 ± 0.01 | 84.53 ± 0.18 |
| dataset 1 | MapSplice | 20 | 10 | 2 | Total | 4.02 ± 0.08 | 0.66 ± 0.01 | 83.69 ± 0.29 |
| dataset 1 | RUM | 20 | 10 | 2 | Rmsk | 5.82 ± 0.84 | 1.04 ± 0.07 | 81.78 ± 3.58 |
| dataset 1 | RUM | 20 | 10 | 2 | Not in Rmsk | 4.95 ± 0.06 | 0.80 ± 0.01 | 83.75 ± 0.33 |
| dataset 1 | RUM | 20 | 10 | 2 | Total | 5.00 ± 0.11 | 0.82 ± 0.01 | 83.68 ± 0.52 |
| dataset 1 | Tophat2 | 20 | 10 | 2 | Rmsk | 1.52 ± 0.21 | 0.91 ± 0.10 | 39.73 ± 3.95 |
| dataset 1 | Tophat2 | 20 | 10 | 2 | Not in Rmsk | 1.21 ± 0.03 | 0.68 ± 0.04 | 43.80 ± 2.07 |
| dataset 1 | Tophat2 | 20 | 10 | 2 | Total | 1.23 ± 0.04 | 0.69 ± 0.04 | 43.73 ± 1.73 |
| dataset 1 | GSNAP | 20 | 20 | 4 | Rmsk | 1.20 ± 0.10 | 0.68 ± 0.06 | 43.63 ± 4.14 |
| dataset 1 | GSNAP | 20 | 20 | 4 | Not in Rmsk | 1.23 ± 0.04 | 0.44 ± 0.00 | 64.32 ± 1.09 |
| dataset 1 | GSNAP | 20 | 20 | 4 | Total | 1.23 ± 0.05 | 0.45 ± 0.01 | 63.23 ± 1.16 |
| dataset 1 | MapSplice | 20 | 20 | 4 | Rmsk | 1.58 ± 0.07 | 0.68 ± 0.07 | 57.13 ± 5.66 |
| dataset 1 | MapSplice | 20 | 20 | 4 | Not in Rmsk | 4.02 ± 0.10 | 0.45 ± 0.01 | 88.72 ± 0.18 |
| dataset 1 | MapSplice | 20 | 20 | 4 | Total | 3.86 ± 0.10 | 0.46 ± 0.00 | 87.95 ± 0.27 |
| dataset 1 | RUM | 20 | 20 | 4 | Rmsk | 4.54 ± 0.86 | 0.80 ± 0.08 | 81.79 ± 4.86 |
| dataset 1 | RUM | 20 | 20 | 4 | Not in Rmsk | 3.90 ± 0.06 | 0.54 ± 0.01 | 86.15 ± 0.33 |
| dataset 1 | RUM | 20 | 20 | 4 | Total | 3.95 ± 0.09 | 0.55 ± 0.01 | 85.94 ± 0.55 |
| dataset 1 | Tophat2 | 20 | 20 | 4 | Rmsk | 1.11 ± 0.13 | 0.62 ± 0.09 | 44.05 ± 1.68 |
| dataset 1 | Tophat2 | 20 | 20 | 4 | Not in Rmsk | 0.82 ± 0.02 | 0.44 ± 0.01 | 46.15 ± 0.41 |
| dataset 1 | Tophat2 | 20 | 20 | 4 | Total | 0.83 ± 0.03 | 0.45 ± 0.02 | 46.19 ± 0.44 |
| dataset 2 | GSNAP | 10 | 10 | 2 | Rmsk | 18.77 ± 0.17 | 17.66 ± 0.19 | 5.92 ± 0.22 |
| dataset 2 | GSNAP | 10 | 10 | 2 | Not in Rmsk | 13.57 ± 0.13 | 11.30 ± 0.14 | 16.70 ± 0.29 |
| dataset 2 | GSNAP | 10 | 10 | 2 | Total | 14.94 ± 0.10 | 12.65 ± 0.10 | 15.36 ± 0.24 |
| dataset 2 | MapSplice | 10 | 10 | 2 | Rmsk | 29.53 ± 0.16 | 21.45 ± 0.17 | 27.38 ± 0.26 |
| dataset 2 | MapSplice | 10 | 10 | 2 | Not in Rmsk | 28.68 ± 0.11 | 13.94 ± 0.11 | 51.40 ± 0.22 |
| dataset 2 | MapSplice | 10 | 10 | 2 | Total | 28.89 ± 0.05 | 15.56 ± 0.09 | 46.16 ± 0.21 |
| dataset 2 | RUM | 10 | 10 | 2 | Rmsk | 37.54 ± 0.27 | 21.30 ± 0.15 | 43.26 ± 0.43 |
| dataset 2 | RUM | 10 | 10 | 2 | Not in Rmsk | 26.95 ± 0.26 | 13.58 ± 0.14 | 49.64 ± 0.08 |
| dataset 2 | RUM | 10 | 10 | 2 | Total | 29.87 ± 0.24 | 15.22 ± 0.10 | 49.05 ± 0.08 |
| dataset 2 | Tophat2 | 10 | 10 | 2 | Rmsk | 36.97 ± 0.07 | 3.71 ± 0.17 | 89.97 ± 0.46 |
| dataset 2 | Tophat2 | 10 | 10 | 2 | Not in Rmsk | 32.34 ± 0.15 | 2.44 ± 0.03 | 92.47 ± 0.12 |
| dataset 2 | Tophat2 | 10 | 10 | 2 | Total | 32.94 ± 0.13 | 2.56 ± 0.02 | 92.23 ± 0.07 |
| dataset 2 | GSNAP | 10 | 20 | 2 | Rmsk | 3.48 ± 0.05 | 2.62 ± 0.04 | 24.62 ± 2.14 |
| dataset 2 | GSNAP | 10 | 20 | 2 | Not in Rmsk | 3.45 ± 0.08 | 1.55 ± 0.05 | 55.07 ± 0.50 |
| dataset 2 | GSNAP | 10 | 20 | 2 | Total | 3.46 ± 0.07 | 1.77 ± 0.03 | 48.89 ± 0.27 |
| dataset 2 | MapSplice | 10 | 20 | 2 | Rmsk | 5.79 ± 0.07 | 3.10 ± 0.03 | 46.43 ± 0.78 |
| dataset 2 | MapSplice | 10 | 20 | 2 | Not in Rmsk | 13.65 ± 0.08 | 1.85 ± 0.03 | 86.48 ± 0.14 |
| dataset 2 | MapSplice | 10 | 20 | 2 | Total | 11.79 ± 0.06 | 2.10 ± 0.02 | 82.18 ± 0.10 |
| dataset 2 | RUM | 10 | 20 | 2 | Rmsk | 12.91 ± 0.33 | 3.13 ± 0.05 | 75.72 ± 0.51 |
| dataset 2 | RUM | 10 | 20 | 2 | Not in Rmsk | 10.48 ± 0.09 | 1.80 ± 0.04 | 82.80 ± 0.30 |
| dataset 2 | RUM | 10 | 20 | 2 | Total | 11.09 ± 0.13 | 2.07 ± 0.04 | 81.34 ± 0.11 |
| dataset 2 | Tophat2 | 10 | 20 | 2 | Rmsk | 8.94 ± 0.09 | 1.76 ± 0.11 | 80.26 ± 1.41 |
| dataset 2 | Tophat2 | 10 | 20 | 2 | Not in Rmsk | 6.04 ± 0.06 | 0.83 ± 0.04 | 86.18 ± 0.62 |
| dataset 2 | Tophat2 | 10 | 20 | 2 | Total | 6.40 ± 0.06 | 0.92 ± 0.03 | 85.56 ± 0.52 |
| dataset 2 | GSNAP | 20 | 10 | 2 | Rmsk | 7.04 ± 0.18 | 5.52 ± 0.14 | 21.54 ± 0.31 |
| dataset 2 | GSNAP | 20 | 10 | 2 | Not in Rmsk | 4.83 ± 0.08 | 2.82 ± 0.11 | 41.71 ± 1.58 |
| dataset 2 | GSNAP | 20 | 10 | 2 | Total | 5.26 ± 0.09 | 3.23 ± 0.09 | 38.65 ± 1.22 |
| dataset 2 | MapSplice | 20 | 10 | 2 | Rmsk | 11.68 ± 0.15 | 6.27 ± 0.10 | 46.32 ± 0.33 |
| dataset 2 | MapSplice | 20 | 10 | 2 | Not in Rmsk | 14.36 ± 0.08 | 3.31 ± 0.07 | 76.92 ± 0.35 |
| dataset 2 | MapSplice | 20 | 10 | 2 | Total | 13.85 ± 0.07 | 3.77 ± 0.07 | 72.81 ± 0.33 |
| dataset 2 | RUM | 20 | 10 | 2 | Rmsk | 25.42 ± 0.49 | 6.22 ± 0.13 | 75.55 ± 0.28 |
| dataset 2 | RUM | 20 | 10 | 2 | Not in Rmsk | 16.60 ± 0.21 | 3.22 ± 0.07 | 80.61 ± 0.29 |
| dataset 2 | RUM | 20 | 10 | 2 | Total | 18.38 ± 0.21 | 3.67 ± 0.07 | 80.03 ± 0.17 |
| dataset 2 | Tophat2 | 20 | 10 | 2 | Rmsk | 18.00 ± 0.74 | 3.57 ± 0.17 | 80.17 ± 0.19 |
| dataset 2 | Tophat2 | 20 | 10 | 2 | Not in Rmsk | 13.79 ± 0.28 | 1.64 ± 0.06 | 88.10 ± 0.43 |
| dataset 2 | Tophat2 | 20 | 10 | 2 | Total | 14.18 ± 0.30 | 1.78 ± 0.06 | 87.45 ± 0.40 |
| dataset 2 | GSNAP | 20 | 20 | 4 | Rmsk | 1.98 ± 0.16 | 1.11 ± 0.05 | 44.08 ± 1.92 |
| dataset 2 | GSNAP | 20 | 20 | 4 | Not in Rmsk | 2.02 ± 0.12 | 0.62 ± 0.04 | 69.41 ± 1.57 |
| dataset 2 | GSNAP | 20 | 20 | 4 | Total | 2.02 ± 0.12 | 0.69 ± 0.04 | 65.68 ± 1.33 |
| dataset 2 | MapSplice | 20 | 20 | 4 | Rmsk | 2.61 ± 0.14 | 1.13 ± 0.05 | 56.60 ± 1.66 |
| dataset 2 | MapSplice | 20 | 20 | 4 | Not in Rmsk | 8.25 ± 0.06 | 0.67 ± 0.01 | 91.94 ± 0.16 |
| dataset 2 | MapSplice | 20 | 20 | 4 | Total | 7.22 ± 0.03 | 0.74 ± 0.01 | 89.82 ± 0.17 |
| dataset 2 | RUM | 20 | 20 | 4 | Rmsk | 10.65 ± 0.54 | 1.14 ± 0.05 | 89.29 ± 0.21 |
| dataset 2 | RUM | 20 | 20 | 4 | Not in Rmsk | 8.18 ± 0.10 | 0.68 ± 0.03 | 91.71 ± 0.31 |
| dataset 2 | RUM | 20 | 20 | 4 | Total | 8.65 ± 0.13 | 0.75 ± 0.03 | 91.37 ± 0.21 |
| dataset 2 | Tophat2 | 20 | 20 | 4 | Rmsk | 4.40 ± 0.30 | 2.06 ± 0.23 | 52.92 ± 6.90 |
| dataset 2 | Tophat2 | 20 | 20 | 4 | Not in Rmsk | 2.39 ± 0.05 | 0.87 ± 0.05 | 63.53 ± 1.90 |
| dataset 2 | Tophat2 | 20 | 20 | 4 | Total | 2.57 ± 0.06 | 0.96 ± 0.04 | 62.80 ± 0.83 |

# Table S18. Alignment statistics for GM12878 RNA-Seq dataset

| **Replicate** | **Statistic** | **Value** |
| --- | --- | --- |
| 1 | Number of Read Pairs Sequenced | 117,876,320 |
| 1 | Number of Read Pairs Aligned | 106,890,066 |
| 1 | Percentage of Read Pairs Aligned | 90.68% |
| 1 | Number of Read Pairs Aligned Uniquely | 100,040,977 |
| 1 | Percentage of Read Pairs Aligned Uniquely | 93.59% |
| 2 | Number of Read Pairs Sequenced | 131,831,897 |
| 2 | Number of Read Pairs Aligned | 124,158,569 |
| 2 | Percentage of Read Pairs Aligned | 94.18% |
| 2 | Number of Read Pairs Aligned Uniquely | 115,844,755 |
| 2 | Percentage of Read Pairs Aligned Uniquely | 93.30% |

# Table S19. RNA-DNA sequence differences found in GM12878.

|  | **Both** | **Replicate 1 Only** | **Replicate 2 Only** | **Total** |
| --- | --- | --- | --- | --- |
| **A>C** | 646 | 2,536 | 1,580 | 4,762 |
| **A>G** | 7,036 | 5,739 | 10,775 | 23,550 |
| **A>T** | 131 | 354 | 128 | 613 |
| **C>A** | 166 | 360 | 166 | 692 |
| **C>G** | 286 | 654 | 505 | 1,445 |
| **C>T** | 492 | 843 | 414 | 1,749 |
| **G>A** | 563 | 856 | 321 | 1,740 |
| **G>C** | 200 | 546 | 330 | 1,076 |
| **G>T** | 169 | 488 | 197 | 854 |
| **T>A** | 132 | 362 | 137 | 631 |
| **T>C** | 1,300 | 2,713 | 1,843 | 5,856 |
| **T>G** | 1,359 | 3,488 | 2,636 | 7,483 |
| **Total** | 12,480 | 18,939 | 19,032 | 50,451 |

# Table S20. Overlap of RNA-DNA sequence differences found in GM12878 with other published studies.

|  | **RNA-DNA sequence differences found in this study** | | |
| --- | --- | --- | --- |
|  | **A-to-G** | **Noncanonical** | **Total** |
|  | 4,484 | 1,513 | 5,997 |
| Ramaswami et al., 2012 | 3,694 (82%) | 12 (0.79%) | 3,706 (62%) |
| Ramaswami et al., 2013 | 2,412 (54%) | 61 (4%) | 2,473 (41%) |
| Bazak et al., 2014 | 3685 (82%) | NA | NA |

**References**

1. Thorvaldsdottir H, Robinson JT, Mesirov JP (2012) Integrative Genomics Viewer (IGV): high-performance genomics data visualization and exploration. Brief Bioinform.
